# Effects of Foods Fortified with Zinc, Alone or Cofortified with Multiple Micronutrients, on Health and Functional Outcomes: A Systematic Review and Meta-Analysis

**DOI:** 10.1093/advances/nmab065

**Published:** 2021-06-24

**Authors:** Becky L Tsang, Erin Holsted, Christine M McDonald, Kenneth H Brown, Robert Black, Mduduzi N N Mbuya, Frederick Grant, Laura A Rowe, Mari S Manger

**Affiliations:** IZiNCG Fortification Task Force; Food Fortification Initiative, Atlanta, GA, USA; IZiNCG Fortification Task Force; Rollins School of Public Health, Emory University, Atlanta, GA, USA; IZiNCG Fortification Task Force; International Zinc Nutrition Consultative Group, Oakland, CA, USA; Department of Pediatrics, University of California San Francisco School of Medicine, San Francisco, CA, USA; IZiNCG Fortification Task Force; Department of Nutrition and Institute for Global Nutrition, University of California, Davis, CA, USA; IZiNCG Fortification Task Force; Johns Hopkins Bloomberg School of Public Health, Baltimore, MD, USA; IZiNCG Fortification Task Force; Global Alliance for Improved Nutrition, Washington, DC, USA; IZiNCG Fortification Task Force; Helen Keller International, Phnom Penh, Cambodia; IZiNCG Fortification Task Force; Food Fortification Initiative, Atlanta, GA, USA; IZiNCG Fortification Task Force; International Zinc Nutrition Consultative Group, Oakland, CA, USA

**Keywords:** zinc, fortification, biomarkers, anthropometry, morbidity, absorption, systematic review

## Abstract

Seventeen per cent of the world's population is estimated to be at risk of inadequate zinc intake, which could in part be addressed by zinc fortification of widely consumed foods. We conducted a review of efficacy and effectiveness studies to ascertain the effect of zinc fortification [postharvest fortification of an industrially produced food or beverage; alone or with multiple micronutrients (MMN)] on a range of health outcomes. Previous reviews have required that the effect of zinc be isolated; because zinc is always cofortified with MMN in existing fortification programs, we did not impose this condition. Outcomes assessed were zinc-related biomarkers (plasma or serum, hair or urine zinc concentrations, comet assay, plasma fatty acid concentrations, and the proportion of and total zinc absorbed in the intestine from the diet), child anthropometry, morbidity, mortality, cognition, plasma or serum iron and copper concentrations, and for observational studies, a change in consumption of the food vehicle. Fifty-nine studies were included in the review; 54 in meta-analyses, totaling 73 comparisons. Zinc fortification with and without MMN increased plasma zinc concentrations (efficacy, *n* = 27: 4.68 μg/dL; 95% CI: 2.62–6.75; effectiveness, *n* = 13: 6.28 μg/dL; 95% CI: 5.03–7.77 μg/dL) and reduced the prevalence of zinc deficiency (efficacy, *n* = 11: OR: 0.76, 95% CI: 0.60–0.96; effectiveness, *n* = 10: OR: 0.45, 95% CI: 0.31–0.64). There were statistically significant increases in child weight (efficacy, *n* = 11: 0.43 kg, 95% CI: 0.11–0.75 kg), improvements in short-term auditory memory (efficacy, *n* = 3: 0.32 point, 95% CI: 0.13–0.50 point), and decreased incidence of diarrhea (efficacy, *n* = 3: RR: 0.79, 95% CI: 0.68–0.92) and fever (efficacy, *n* = 2: RR: 0.85, 95% CI: 0.74–0.97). However, these effects cannot be solely attributed to zinc. Our review found that zinc fortification with or without MMN reduced the prevalence of zinc deficiency and may provide health and functional benefits, including a reduced incidence of diarrhea.

## Teaser

Zinc fortification increased plasma/serum zinc concentrations and reduced the prevalence of zinc deficiency; limited evidence indicated that MMN + zinc-fortified food increased body weight and reduced the incidence of fever and diarrhea.

## Introduction

Zinc is an essential trace element that is involved in numerous aspects of cell metabolism by functioning as a catalyst, structural element, and regulator of gene expression (1). Through these roles, zinc supports immune competence, normal physical growth and neurobehavioral development, and reproductive function (2). As the body can rapidly mobilize only a small amount of endogenous zinc for metabolism, a regular intake of adequate amounts of zinc is needed to maintain physiological functions (2).

The most bioavailable dietary sources of zinc are animal-source foods; the zinc content of plant-based foods is dependent on soil zinc concentrations, and zinc uptake may be affected by absorption inhibitors present in these foods (2). As a result of low accessibility to animal-based foods in many populations and the limitations of plant foods as sources of zinc, 17% of the global population is estimated to be at risk of inadequate zinc intake (3). Postharvest food fortification, the addition of essential micronutrients to staple foods during food processing (such as wheat flour milling), is considered a highly cost-effective intervention to improve the dietary intake of micronutrients (4). One hundred and forty-seven countries have mandated fortification of ≥1 staple food (i.e., wheat flour, maize flour, edible oil, rice, salt) (5). However, zinc is not a universally included micronutrient in many countries where fortification standards exist, despite indications of deficiency. As of June 2020, inclusion of zinc was mandatory in 21 of 39 low- and lower-middle-income countries with wheat flour fortification standards, 9 of 10 countries with maize flour fortification standards, and 2 of 4 countries with rice fortification standards (6).

Food fortification provides a potential opportunity to enhance zinc intake, but it remains unclear whether zinc fortification leads to improved health outcomes (1). Previous reviews of zinc fortification that included zinc fortification of infant formula or complementary foods (7) (foods not intended for the broader population) were nonsystematic (8), or were limited in scope (9). A 2016 Cochrane review and meta-analysis by Shah et al. (9) included a range of health outcomes, including biomarkers of zinc status, anthropometry, cognition, and adverse effects. It found a statistically significant increase in plasma/serum zinc concentration (PZC) after zinc fortification interventions from 3 eligible studies in the meta-analysis, and either limited or no evidence regarding the prevalence of zinc deficiency, anthropometry, cognition, and adverse effects. Shah et al. (9) limited the review to studies that could attribute outcomes to zinc fortification alone, i.e., nonfortified foods compared with zinc-only fortified foods, or multiple micronutrient (MMN)-fortified foods without zinc compared with MMN-fortified foods with zinc. However, zinc fortification is rarely implemented without other micronutrients; thus, including studies of MMN fortification including zinc versus nonfortified food could provide additional insights, even though it is not possible to attribute any functional responses specifically to zinc with this study design. Nevertheless, the absence of a functional benefit or adverse outcome under these study conditions would suggest that zinc is not contributing to such outcomes. The objective of this review was to conduct an updated systematic review and meta-analysis of the impact of fortifying foods with zinc (alone or in addition to multiple nutrients) on biomarkers of zinc status and multiple health outcomes, considering a variety of study designs.

## Methods

This systematic review followed the guidelines from the Cochrane Handbook for Systematic Reviews (10) and adhered to the Preferred Reporting Items for Systematic Reviews and Meta-Analyses (PRISMA) statement (11). The protocol was drafted at the start of the review and any amendments after that time were documented (**Supplemental Method**).

### Search strategy

With the assistance of a research librarian, we conducted a search of the following databases for English-language literature, with no time limitation: PubMed, Embase, Scopus, Agricultural & Environmental Science Collection, Agricola, CAB Abstracts, and Web of Science. The search strategy for PubMed included search terms and Medical Subject Headings (MeSH) in the following areas: fortification, enrichment (fortif*[tiab] OR enrich*[tiab]), zinc zinc[tiab], and human studies only [NOT (“animals”[MeSH Terms] NOT “humans”[MeSH Terms])]. This method was adapted for other databases as appropriate (for full search strings specific to respective databases see Supplemental Method). We also accepted studies published after the search was conducted, if identified by coauthors.

#### Inclusion and exclusion criteria

Eligible study designs were placebo- and nonplacebo-controlled trials, cohort studies, and cross-sectional studies with pre and postintervention measurements. Acceptable comparisons were: no food/intervention compared with fortified food [zinc only or MMN including zinc (MMN + zinc)], nonfortified food compared with fortified food (zinc only or MMN + zinc), fortified food without zinc compared with fortified food with zinc, and single pre and postfortification measurements. Population eligibility criteria were male or female, of any age, regardless of baseline zinc status. Studies were excluded if the participants were selected for pre-existing health conditions, except for anemia, zinc deficiency, and stunting. Zinc fortification was defined as the addition of zinc (alone or in combination with other micronutrients) at the postharvest, industrial food processing stage, to a food for human consumption. Study outcomes were zinc-related biomarkers (PZC expressed both as a continuous outcome and as prevalence of deficiency and as defined by authors, hair or urine zinc concentrations, comet assay, plasma fatty acid concentrations, and the proportion of and total zinc absorbed in the intestine from the diet), child anthropometry [height, weight, midupper arm circumference (MUAC), height-for-age z-score (HAZ), weight-for-age z-score (WAZ), weight-for-height z-score (WHZ), prevalence of stunting, wasting, and underweight], morbidity (as defined by the trial authors), mortality, cognition, effect on iron status (measured by plasma or serum ferritin), and effect on copper status (measured by copper biomarkers as defined by authors). The proportion of and total zinc absorbed in the intestine from the diet are referred to, respectively, as fractional zinc absorption (FAZ) and total absorbed zinc (TAZ), and include both zinc added through fortification (extrinsic zinc) as well as zinc naturally occurring in the food (intrinsic zinc). All methods of measuring FAZ were considered eligible (12).

Since effectiveness studies did not control adherence to the intervention, we also included the following outcome: change in consumption amount or coverage (proportion of population consuming the food) of the food after fortification. For the assessment of the effect on serum ferritin, acceptable comparisons were nonfortified food compared with fortified food with zinc, or iron-fortified food (with or without other MMN) compared with iron + zinc-fortified food (with or without other MMN).

Studies of therapeutic use of zinc-fortified food, biofortification with zinc, and zinc tablet/pill/syrup supplementation, including point-of-use fortification with micronutrient powders or lipid-based supplements, were not eligible for inclusion in the review. As the focus of this review was the effect of zinc fortification through large-scale food fortification for the general population, we did not include infant formula, toddler milk, or complementary foods unless all ingredients were clearly stated, contained a single cereal ingredient, and did not contain legumes.

We contacted study authors for additional information if it was missing or not presented in the format required for this review. Unpublished data from contacted authors was also eligible for inclusion. If authors did not respond, we followed-up once via e-mail. If there was no response from authors, the study was not included for the nonreported outcome.

### Selection of studies

Two review authors (EH, BLT) independently screened all titles and abstracts using *Covidence* software (13). When a title or abstract could not be rejected with certainty, the full text of the article was obtained for further evaluation. BLT and EH independently screened full-text records for final assessment of eligibility. Disagreements at any stage of the eligibility assessment process were resolved through discussion and consultation with a third author (MSM) when necessary.

### Data extraction

Two review authors (EH, BLT) each extracted data from half of the eligible studies using a standardized abstraction form and checked each other's work for accuracy. The following data were recorded in the abstraction form: study design, study location, sample size, study years, participant characteristics (age, sex, physiological status), baseline zinc status (mean PZC and/or presence of zinc deficiency), zinc biomarker assay methods, intervention characteristics (zinc dose per day and duration, zinc compound, cofortification of other nutrients), cointerventions other than fortification, comparison group, and all outcomes of interest as described in the inclusion criteria.

During data extraction, we classified studies as “efficacy” if they were controlled or nonplacebo-controlled trials where participants were known to consume food fortified with zinc under carefully dosed and measured conditions, i.e., fortified food prepared by the study investigators and the amount of fortified food consumed was known. We classified studies as “effectiveness” if they were controlled or nonplacebo-controlled trials where participants or households were provided food fortified with zinc, but investigators may not have had control over how participants/households stored, prepared, and cooked the food, who consumed the food, regardless of the target population, and how much of the food was consumed/wasted. Effectiveness studies also included controlled or noncontrolled cohort studies or population-based studies where it may have been unknown whether participants consumed a food fortified with zinc, but mandatory legislation of a food fortified with zinc was in place and a prepost evaluation of the fortification program was conducted.

All PZC were converted to μg/dL and MUAC to centimeters if they were presented in a different unit. We extracted daily dose of zinc as the amount of zinc provided by the fortified food over the course of a day, as reported by authors. In the case of effectiveness studies, where intake of the food was not controlled, if the authors provided daily food intake and the fortification concentration, then we calculated the daily dose of zinc. Seventy-one per cent of studies reported just the extrinsic (fortificant) zinc dose; the remainder reported total daily zinc doses that also included zinc intrinsic to the food vehicle.

### Quality assessment of studies and overall quality of evidence

The National Heart, Lung, and Blood Institute's (NHLBI) Study Quality Assessment Tools (14), which features separate tools by study design, was used to derive a Good, Fair, or Poor score for each study. For controlled bioavailability studies, the NHLBI Controlled Intervention Studies tool was adapted to consider features of bioavailability study designs that could affect quality. BLT and MSM conducted the quality assessment in duplicate, EH conducted quality assessments where there were conflicts of interest, and RB resolved any disputes or contradictions. BLT conducted the overall quality of evidence assessment using Grading of Recommendations Assessment, Development, and Evaluation (GRADE) methodology (15) in GRADEpro (16) software.

### Data synthesis for statistical analysis

Where the study's effect estimate was directly reported (OR, rate ratio), we entered data directly into Review Manager 5 (RevMan) software (17). If the study reported a prevalence instead of an effect estimate (e.g., percentage of population with zinc deficiency), the number of cases was calculated to generate an unadjusted OR on a natural log scale; the unadjusted ORs were used in data analysis. For continuous data, we entered the data as arithmetic means and SDs; where studies reported alternate central measures of tendencies or errors, we used published formulae (10, 18) to convert values from geometric means or medians to arithmetic means, and ranges or 95% CIs to SDs. In the case of 1 missing SD (19), we imputed the SD by taking the average SD at end line from 3 similar studies. In 1 study where it appeared that the authors mislabeled the type of variance (20), we assumed it was the SD for analysis. In 1 study (21), FAZ from meals and the fortified food (milk) was only reported separately; in order for comparison with the baseline FAZ value (which included nonfortified milk in the usual diet), we calculated FAZ from the TAZ summary estimate and total zinc provided from the meal and fortified food.

In 4 studies (20, 22–25), in which the intervention arms did not differ by zinc fortification details, we combined the data for 2 study locations/intervention arms using a published formula (26). As the magnitude of change at end line may be masked if there are statistically significant differences at baseline between intervention groups, we increased/decreased end-line values for the fortification intervention group to remove any statistically significant differences at baseline. This was done for 3 studies (27–29) for PZC.

### Statistical analysis

We conducted a meta-analysis for an outcome if >1 study assessed the same outcome, and we pooled results from efficacy studies and effectiveness studies separately. We conducted analyses in RevMan using a random-effects model, anticipating that there would be natural heterogeneity between studies that differed according to populations, dietary patterns, doses, durations, fortification vehicles, and implementation/delivery strategies. For both continuous and dichotomous variables, the inverse variance method was used. We pooled end-line values and change from baseline values from similar study designs but presented them as subgroups in the main meta-analysis. To avoid double-counting controls where there were multiple intervention arms, we divided the control population across the intervention arms. We considered heterogeneity across studies for an outcome *moderate* if the *I^2^* statistic was 40–75% and *P* <0.05 and *serious* if the *I^2^* statistic was >75% and *P* <0.05 [modified from Cochrane guidance (26)]. If heterogeneity was moderate or serious, we conducted subgroup analyses if there were a minimum of 4 studies for an outcome, with a minimum of 2 studies in each subgroup.

We conducted the following subgroup analyses: study quality by NHLBI score (Good, Fair, Poor), food vehicle (cereals, beverages, or condiments), daily zinc dose [above or below the median International Zinc Nutrition Consultative Group's (IZiNCG) Estimated Average Requirement (EAR) contribution in a mixed or refined plant-based diet (30) for a given age and gender], study design, duration (above or below the median duration for the study design), baseline age and sex [<2 y; preschool-age children 2–4 y (PSAC); school-age children from 5 to 11 y (SAC); female adolescents and women of reproductive age (WRA) from 12 to 49 y; male adolescents and men aged 12–49 y; individuals aged 50 y and older; age and sex categories were collapsed in subgroup analyses if there were not enough data for a single category (e.g., <5 y or 5 y and older)], baseline zinc status of the study population (≥50% or <50% zinc deficient, as defined by trial authors, or mean PZC above or below the IZiNCG cut-offs for deficiency by population), baseline stunting prevalence [≥20% stunted or <20% stunted (1, 31, 32)], and MMN and zinc comparisons (MMN + zinc compared with nonfortified food/no food, MMN + zinc compared with MMN, or zinc compared with nonfortified food).

## Results

### Study characteristics

**FIGURE 1:**
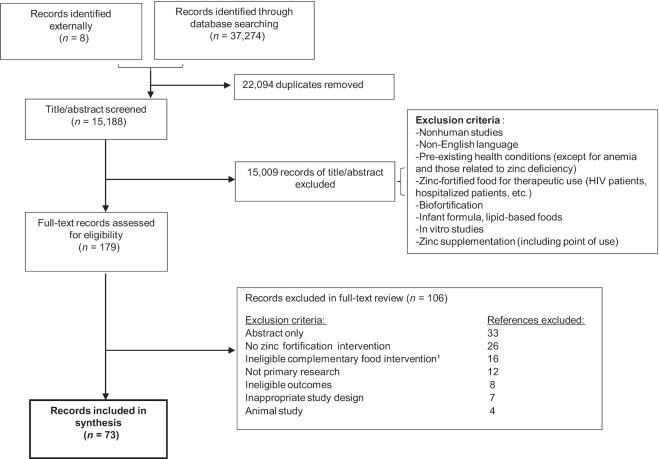
Preferred Reporting Items for Systematic Reviews and Meta-Analyses flow diagram. ^1^Studies involving complementary foods were only included if all ingredients were clearly stated, if the food contained a single cereal ingredient, or did not contain legumes.

The search strategy yielded a total of 37,274 records; after removal of duplicate records, 15,184 records remained for title and abstract review. The full PRISMA record management flow is presented in **Figure 1**. After contacting authors and searching reference lists of previous zinc fortification reviews (7–9), we found a total of 73 records that were eligible. After accounting for multiple records of the same study (e.g., if outcomes were reported in separate records), we included 59 unique studies. All included records, the outcomes reported, and the foods fortified are in **Supplemental Table 1**.

Broadly, there were 3 categories of fortification vehicles: 33 studies in cereal grains (wheat flour/products, *n* = 17; maize flour/products, *n* = 5; rice/rice flour, *n* = 10; unknown flour, *n* = 1), 21 in beverages (milk, *n* = 12; other beverages, *n* = 9), and 3 in condiments (seasoning powder, *n* = 2; salt, *n* = 1); 2 additional studies provided participants with both fortified cereal grains and beverages. Three studies included MMN with zinc but had a comparison that allowed for the isolation of the effect of zinc [i.e., type and concentrations of MMN (except for zinc) remained the same in the comparison], 11 studies fortified foods with only zinc, and 45 studies included cofortification with MMN (and in some cases, nonmicronutrients such as ω-3 fatty acids), compared with a nonfortified/fewer MMN-fortified food (or no food, *n* = 2). Where reported, the most commonly utilized zinc compounds (5 studies included 2 compounds) were zinc sulfate (*n* = 16) and zinc oxide (*n* = 16), followed by zinc gluconate (*n* = 3), zinc chloride (*n* = 2), and zinc acetate (*n* = 2). Other compounds used included amino-chelated zinc, zinc dioxide, zinc glycinate chelate, and zinc lactate. There was a wide range in zinc doses, from 0.7 mg/d to 54.4 mg/d (median, 4.37 mg/d), representing 17–1088% of the EAR for zinc in the respective study populations. Where reported, we did not consider total zinc provided through the overall diet, as this was only available in a very small number of studies. With the exception of zinc stable isotope tracer studies of zinc absorption that included just a day or 2 of fortified food intake, study durations ranged from 27 d to 3 y.

Seventy-one per cent of studies were primarily conducted in low- or lower-middle-income countries (World Bank classification for the year the study took place or year of publication) and 52.5% in South Asia or East Asia and Pacific regions (UNICEF regions). Twelve studies specifically screened for zinc deficiency (based on PZC) and only included deficient participants. The greatest number of studies (25, 42.4%) were conducted in SAC (5–11 y) populations, followed by 20 (32.2%) in WRA, 8 (13.6%) in PSAC, and 3 studies (5.1%) included children aged under 2 y. Two studies enrolled pregnant women, and 1 study each enrolled adults over 50 y, male and female adults aged 12–49, or men only.

We classified 32.1% of studies included in meta-analyses as being of Good quality, 43.5% of Fair quality, and 24.5% of Poor quality (**Supplemental Tables 2–5**).

### Meta-analysis results

Key outcomes (PZC, prevalence of zinc deficiency, weight, and diarrhea), their absolute effects, alongside study details and GRADE score are presented in a Summary of Findings table (**Table 1**).

#### PZC (μg/dL) and prevalence of zinc deficiency

**FIGURE 2:**
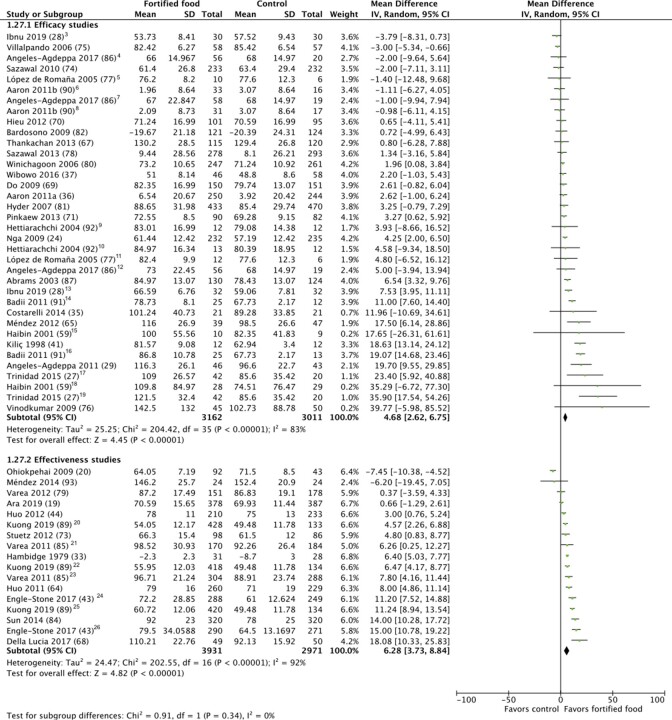
Effect of foods fortified with zinc, alone, or cofortified with multiple micronutrients, on plasma/serum zinc concentrations.^1,2^ ^1^Studies with multiple intervention arms included in meta-analysis are specified in footnotes. Unless noted otherwise, the comparison group was a nonfortified food. ^2^End-line values and mean difference values have been combined in analysis. ^3^Participant group: nonanemic. ^4^Intervention arm: low (3 beverages/wk, ∼2.4 mg/d zinc). ^5^Intervention arm: flour fortified with 30 mg zinc/kg; control: flour fortified with iron. ^6^Intervention arm: bread providing 7.5 mg zinc/d, iron and folic acid; control: flour fortified with iron and folic acid. ^7^Intervention arm: high (7 beverages/wk, ∼5.4 mg/d zinc). ^8^Intervention arm: bread providing 15 mg zinc/d, iron and folic acid; control: flour fortified with iron and folic acid. ^9^Intervention arm: rice flour fortified with zinc, iron as iron sulfate (FeSO_4_), folic acid, and disodium EDTA (Na_2_EDTA); control: rice flour fortified with iron as iron sulfate (FeSO_4_), folic acid, and disodium EDTA (Na_2_EDTA). ^10^Intervention arm: rice flour fortified with zinc, iron as iron sulfate (FeSO_4_), and folic acid; control: rice flour fortified with iron as iron sulfate and folic acid. ^11^Intervention arm: flour fortified with 90 mg zinc/kg and iron; control: flour fortified with iron. ^12^Intervention arm: moderate (5 beverages/wk, ∼4 mg/d zinc). ^13^Participant group was anemic. ^14^Intervention arm: flour fortified at 100 mg zinc/kg. ^15^Intervention arm: biscuits fortified with zinc, calcium, vitamin D, and iron; control: biscuits with calcium, vitamin D, iron. ^16^Intervention arm: flour fortified at 50 mg zinc/kg. ^17^Intervention arm: 1 glass of fortified milk; control: water. ^18^Intervention arm: biscuits fortified with zinc, calcium, vitamin D; control: biscuits with calcium and vitamin D. ^19^Intervention arm: 2 glasses of fortified milk; control: water. ^20^Intervention arm: UltraRice New. ^21^Participant group: 1–2 y. ^22^Intervention arm: UltraRice Original. ^23^Participant group: 2–6 y. ^24^Participant group: 12–59 mo. ^25^Intervention group: NutriRice. ^26^Participant group: women 15–49 y.

**FIGURE 3:**
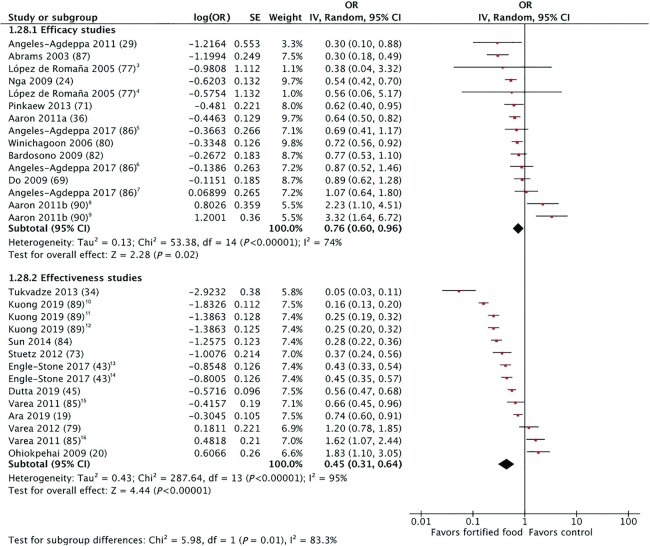
Effect of foods fortified with zinc, alone, or cofortified with multiple micronutrients, on zinc deficiency.^1,2^ ^1^Studies with multiple intervention arms included in meta-analysis are specified in footnotes. Unless noted otherwise, the comparison group was a nonfortified food. ^2^End-line values and mean difference values have been combined in analysis. ^3^Intervention arm: flour fortified with 90 mg zinc/kg and iron; control: flour fortified with iron. ^4^Intervention arm: flour fortified with 30 mg zinc/kg and iron; control: flour fortified with iron. ^5^Intervention arm: moderate (5 beverages/wk, ∼4 mg/d zinc). ^6^Intervention arm: high (7 beverages/wk, ∼5.4 mg/d zinc). ^7^Intervention arm: low (3 beverages/wk, ∼2.4 mg/d zinc). ^8^Intervention arm: bread providing 15 mg zinc/d, iron, and folic acid; control: flour fortified with iron and folic acid. ^9^Intervention arm: bread providing 7.5 mg zinc/d, iron, and folic acid; control: flour fortified with iron and folic acid. ^10^Intervention arm: NutriRice. ^11^Intervention arm: UltraRice New. ^12^Intervention arm: UltraRice Original. ^13^Participant group: women 15–49 y. ^14^Participant group: 12–59 mo. ^15^Intervention group: 2–6 y. ^16^Participant group: 1–2 y.

Regardless of study design (efficacy or effectiveness), the effect of zinc-fortified food on PZC and prevalence of zinc deficiency was statistically significant, indicating an increase in PZC (**Figures 2** and **3**) and protective effect on zinc deficiency. The details of the studies included in these meta-analyses are provided in **Supplemental Table 6**. The mean increase in PZC over the course of the intervention periods was statistically significant in effectiveness studies (*n* = 13) (6.28 μg/dL; 95% CI: 3.72–8.84 μg/dL; low-quality evidence) compared with efficacy studies (*n* = 27) (4.68 μg/dL; 95% CI: 2.62–6.75 μg/dL; low-quality evidence). Similarly, the protective effect on the prevalence of zinc deficiency was statistically significant in effectiveness studies (*n* = 10) (OR: 0.45; 95% CI: 0.31–0.64; low-quality evidence) compared with efficacy studies (*n* = 11) (OR: 0.75; 95% CI: 0.60–0.96; very low-quality evidence).

Heterogeneity was serious for PZC (*I^2^* >75%, *P* <0.05) and prevalence of zinc deficiency (*I^2^* = 74%, *P* <0.05) in efficacy studies, and serious for both outcomes in effectiveness studies; heterogeneity was explored through subgroup analyses. The subgroup analysis for study quality found that there was no statistically significant change in PZC after fortification in efficacy studies rated of Good quality (*n* = 11). The change was statistically significant in Fair-quality studies (*n* = 7) (6.04 μg/dL; 95% CI: 1.34–10.74 μg/dL), with a trend towards a greater effect in Poor-quality studies (*n* = 9) (11.28 μg/dL; 95% CI: 6.22–16.33 μg/dL) (**Supplemental Figure 1**). In efficacy study subgroup analyses, the increase in PZC was greater for studies with intervention periods <6 mo (*n* = 14) (7.24 μg/dL; 95% CI: 4.00–10.48 μg/dL), whereas there was no statistically significant effect in studies of ≥6 mo duration (*n* = 13) (1.72 μg/dL; 95% CI: −0.26–3.71) (**Supplemental Figure 2**). A similar trend was seen in the effectiveness study subgroup analysis for duration: studies with shorter intervention duration (<12 mo) (*n* = 5) (OR: 0.28; 95% CI: 0.17–0.44) also had a greater effect on zinc deficiency than those with interventions ≥12 mo (*n* = 5) (OR: 0.70; 95% CI: 0.52–0.94), although both were significant (**Supplemental Figure 3**).

In subgroup analyses of efficacy studies by comparison group [comparing MMN + zinc to nonfortified/no food control groups (*n* = 20), comparing MMN + zinc to MMN (*n* = 4), or comparing zinc-only fortified foods to nonfortified foods (*n* = 3)], the increase in PZC was statistically significant and highest when zinc was provided alone (15.78 μg/dL; 95% CI: 10.52–21.05 μg/dL). There was a smaller, statistically significant increase in PZC when MMN + zinc-cofortified foods were compared with nonfortified versions/no foods (2.99 μg/dL; 95% CI: 1.03–4.95 μg/dL) but no statistically significant effect in studies of MMN + zinc versus MMN only (−0.53 μg/dL; 95% CI: −3.63–2.57 μg/dL) (**Supplemental Figure 4**).

In efficacy studies, there was no statistical difference in the effect on PZC or zinc deficiency by food vehicle group (cereal grains, beverages, and condiments) (**Supplemental Figures 5–6**). The remaining subgroup analyses for efficacy and effectiveness studies for the prevalence of PZC or zinc deficiency were not statistically significant or had an insufficient number of studies in each subgroup category for analysis.

#### Child anthropometry

**FIGURE 4:**
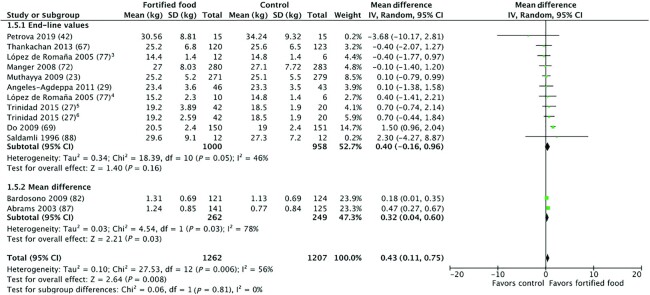
Effect of foods fortified with zinc, alone, or cofortified with multiple micronutrients, on child weight (kg), efficacy studies.^1,2^ ^1^Studies with multiple intervention arms included in meta-analysis are specified in footnotes. Unless noted otherwise, the comparison group was a nonfortified food. ^2^End-line values and mean difference values have been combined in analysis. ^3^Intervention arm: flour fortified with 90 mg zinc/kg and iron; control: flour fortified with iron. ^4^Intervention arm: flour fortified with 30 mg zinc/kg and iron; control: flour fortified with iron. ^5^Intervention arm: 2 glasses of fortified milk; control: water. ^6^Intervention arm: 1 glass of fortified milk; control: water.

Included studies with child anthropometry outcomes are detailed in **Supplemental Table 7**. As all studies but 1 (27) compared the fortified food to a nonfortified food, differences in calorie intake were not expected to be a confounding factor (sensitivity analyses removing this article did not cause any changes in results). Provision of zinc-fortified foods in efficacy studies (*n* = 11) resulted in a statistically significant increase in weight (0.43 kg; 95% CI: 0.11–0.75 kg; low-quality evidence) (**Figure 4**). Meta-analyses for all other child anthropometry outcomes (height, MUAC, HAZ, WAZ, WHZ, and prevalences of stunting, wasting, and underweight) for efficacy studies, and where possible, for effectiveness studies were not statistically significant (**Supplemental Figures 7–17**). Where heterogeneity was serious, we conducted subgroup analyses. Although the overall height meta-analysis was not significant, subgroup analysis by study duration found a statistically significant smaller height increase in children who received zinc-fortified foods in studies of <6 mo duration (−0.97 cm; 95% CI: −1.21 to −0.72 cm) compared with children who received zinc-fortified foods in studies ≥6 mo duration (0.18 cm; 95% CI: −0.05 to 0.41 cm) (**Supplemental Figure 18**).

#### Cognition

Cognition outcomes varied widely, both with regard to the outcomes measured in different studies and the assessment tools used. Across 10 studies, 25 cognitive outcomes were reported (**Supplemental Table 8**). Only 3 outcomes were reported in >1 study (all efficacy studies): the Wechsler Intelligence Scale for Children (WISC) test's digit span forward (*n* = 3), digit span backward (*n* = 3), and coding score change (*n* = 3). Fortified food with zinc had a statistically significant increase in the digit span forward score, which is a measurement of short-term auditory memory (0.32 point; 95% CI: 0.13–0.50 point; moderate-quality evidence) (score maximum of 14) (**Supplemental Figure 19**). There was no statistically significant increase or decrease in digit span backward (*n* = 3) and coding scores (*n* = 3) (**Supplemental Figures 20** and **21**). No subgroup analyses were conducted due to an insufficient number of studies.

#### Morbidity

**FIGURE 5:**
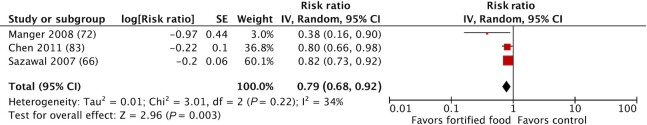
Effect of foods fortified with zinc, alone, or cofortified with multiple micronutrients, on diarrhea episodes, efficacy studies.

**FIGURE 6:**
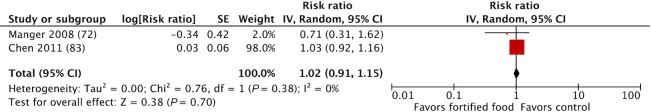
Effect of foods fortified with zinc, alone, or cofortified with multiple micronutrients, on fever episodes, efficacy studies.

Across the various morbidity outcomes (**Supplemental Table 9**) reported, we had adequate data to conduct meta-analyses for episodes of diarrhea (*n* = 3), vomiting (*n* = 2), nausea (*n* = 2), stomach pain (*n* = 2), fever (*n* = 2), skin rash (*n* = 2), upper respiratory tract infections (URTI) (*n* = 2), lower respiratory tract infections (LRTI) (*n* = 2), and other reported morbidities (headaches, constipation, *n* = 2). The reductions in risk of diarrhea (RR: 0.79; 95% CI: 0.68–0.92; low-quality evidence) and fever episodes (RR: 0.85; 95% CI: 0.74–0.97; low-quality evidence) were statistically significant (**Figures 5** and **6**); there was no statistically significant effect of fortification on URTI, LRTI, vomiting, nausea, or stomach pain (**Supplemental Figures 22–28**). No subgroup analyses were conducted due to an insufficient number of studies.

#### FAZ and TAZ

Six studies were included in FAZ and TAZ meta-analyses (**Supplemental Table 10**). There was a statistically significant reduction in FAZ associated with the consumption of zinc-fortified food (−0.08; 95% CI: −0.15 to −0.01; very low-quality evidence) (**Supplemental Figure 29**) and an increase in TAZ (0.93 mg; 95% CI: 0.47–1.40 mg; very low-quality evidence) compared with unfortified control food (**Supplemental Figure 30**). Subgroup analyses found that the reduction in FAZ was only statistically significant for fortified cereal grains (−0.11; 95% CI: −0.18 to −0.04) (**Supplemental Figure 31**) and when the fortified food provided 50% or more of the EAR (−0.15; 95% CI: −0.21 to −0.10) (**Supplemental Figure 32**). The increase in TAZ was statistically significant regardless of the proportion of EAR contributed through the food but was higher in studies that contributed >50% EAR (1.61 mg; 95% CI: 0.71–2.52 mg) compared with <50% EAR (0.20 mg; 95% CI: 0.04–0.36 mg) (**Supplemental Figure 33**). There was no difference in TAZ by food vehicle (**Supplemental Figure 34**).

#### Effects on biomarkers of iron (plasma/serum ferritin) and copper status

Four efficacy studies (zinc doses ranging from 3.0 to 54.5 mg/d) measured plasma/serum ferritin; 2 studies compared a zinc-only fortified food to a nonfortified food; the other 2 studies cofortified with iron in both study arms (zinc + iron compared with iron) (**Supplemental Table 11**). None adjusted serum ferritin concentrations for inflammation; no studies fortified a food with copper. In studies where only zinc was provided, there was a statistically significant increase in plasma/serum ferritin (4.56 μg/L; 95% CI: 3.23–5.89 μg/L; moderate-quality evidence) (Supplemental Figure 31). In the 2 studies where iron was provided in all study arms (zinc:iron ratios were 0.85 in 3 arms; 2.56 in 1), cofortification with zinc was neither superior nor inferior to fortification with iron alone. Two efficacy studies and effectiveness studies each measured plasma/serum copper concentrations. Neither meta-analysis found a statistically significant increase or decrease in plasma/serum copper from consuming a fortified food with zinc (Supplemental Figures 32 and 33); very low-quality (effectiveness studies) and very low-quality (efficacy studies) evidence. We did not conduct subgroup analyses on plasma/serum ferritin or copper concentrations due to an insufficient number of studies.

#### Hair and urine zinc concentrations

Two effectiveness studies (**Supplemental Table 12**) reported hair and urine zinc concentrations; 1 study (33) was controlled whereas the other (34) was a prepost comparison. The effect of fortification on zinc concentrations in hair was significant (22.71 μg/g; 95% CI: 11.91–33.51 μg/g; very low-quality evidence) (**Supplemental Figure 35**). There was also a statistically significant increase in zinc concentrations in urine [78.10 μg/(dL · 24 h); 95% CI: 52.6, 104 μg/(dL · 24 h); very low-quality evidence] (Supplemental Figure 34).

### Narrative results

The cognition and morbidity outcomes which could not be pooled in meta-analyses are detailed by study in Supplemental Tables 8 and 9. For cognition, 9/11 studies reported a statistically significant, positive effect in ≥1 cognition outcome for the fortified group compared with the nonfortified group. One study found no significant differences by intervention for any cognitive test (35); another study (36) was not considered eligible for cognition outcomes as it did not describe any of the methods or results, but stated that there was no effect of the intervention on any cognitive abilities. One study (25) found that vitamin A and zinc deficiency were the only 2 end-point variables that contributed significantly to a modeled end-point Raven Coloured Progressive Matrices Test score (a measure of fluid reasoning).

Compared with the meta-analysis results, narrative review results were inconsistent for diarrhea, fever, and URTI, making it difficult to draw an overall conclusion for these outcomes. The only study that included mortality and hospitalizations (35) was also the only study to use a zinc-only fortified food (milk). Twenty-one nursing home residents participated in a crossover trial, consuming zinc-fortified or nonfortified milk for 2 mo, with a 15-d wash-out period in between. Compared with other nursing home residents who did not participate in the trial, mortality and hospitalizations were lower in the participants in the 1-y follow-up period after the end of the intervention. It is not clear whether those who did not participate in the crossover trial also received nonfortified milk in regular meals. If they did, the effects on morbidity are confounded by the calories and nutrients inherent to the milk itself.

There were insufficient or incomparable data to conduct meta-analyses for immune system biomarkers (*n* = 6), plasma fatty acids (*n* = 3), and change in fortified food intake after fortification (*n* = 3). Immune system biomarkers (and the methodologies for inducing immune system reactions) differed widely across studies. Three studies (37–39) found no significant differences between intervention and control groups in the measured immune system biomarkers, whereas 3 other studies (35, 40, 41) found significant differences in 1 or more of the biomarkers evaluated (**Supplemental Table 13**). All studies (23, 37, 42) assessing impact on plasma fatty acids found an increase in the various fatty acid biomarkers evaluated; however, all of these foods were also fortified with PUFAs (**Supplemental Table 14**). Studies assessing any differences in consumption of the food vehicle after fortification was introduced found no change in mean usual intake (43), proportion of fortified flour to overall flour consumption (44), or finishing a school meal (45).

Studies with FAZ/TAZ outcomes but without appropriate comparison groups (e.g., no baseline measurement or nonfortified food control) were not included in meta-analyses (**Supplemental Table 15**). These studies varied by their study objective and compared zinc-fortified food in relation to the inclusion of iron, zinc fortification concentrations, or type of zinc compounds.

## Discussion

This is the fourth review to report an increase in PZC and the first to report a reduction in the prevalence of zinc deficiency in studies of zinc-only or MMN + zinc-fortified foods, suggesting that zinc fortification is an efficacious and effective delivery mechanism for improving biochemical zinc status and reducing the prevalence of zinc deficiency, across multiple types of food vehicles. Whereas past reviews (7–9) required that the effect of zinc fortification be isolated, the broader eligibility criteria of our review more closely reflects how zinc is delivered in large-scale food fortification programs. In particular, all countries that currently include zinc in their mandatory or voluntary fortification standards also include other vitamins or minerals (46). As a result, this review included a total of 73 records (59 unique studies) compared with 9 in the 2016 Cochrane Review by Shah et al. (9).

Similar to Shah et al. (9), who reported a mean PZC increase of 13.9 μg/dL (2.12 μmol/L), our review found a consistent positive effect of zinc fortification on PZC, both when zinc was provided alone or with other micronutrients; however, our results showed a lower magnitude of effect across both efficacy (4.68 μg/dL) and effectiveness studies (6.28 μg/dL). When stratified by studies where the effect of zinc could be isolated, the effect on PZC in efficacy studies was closer (15.78 μg/dL) to that reported by Shah et al. (9). Whereas Shah et al. (9) were not able to include prevalence of zinc deficiency as an outcome due to an insufficient number of studies, we found a protective effect of zinc fortification on the prevalence of zinc deficiency in both efficacy and effectiveness studies.

The most concerning subgroup analysis finding was by study quality for efficacy studies – the impact on PZC disappeared when considering Good-quality studies and only remained for Fair- and Poor-quality studies. The funnel plot for PZC in efficacy studies (not shown) indicates that smaller studies were more likely to report a statistically significant impact, indicating that there may be publication bias present (e.g., small studies with nonsignificant results not published). It was unclear why shorter study duration in efficacy studies would lead to a greater increase in PZC or lower prevalence of zinc deficiency in effectiveness studies. Compared with longer duration efficacy studies (≥6 mo), shorter duration studies were not more likely to provide higher doses of zinc. Reduction in participant adherence to the intervention or compliance to a fortification program over time may be a confounding factor. PZC responds relatively quickly to supplementation and withdrawal (47); if there is any reduction in participant adherence during a longer study, then there may be corresponding attenuation of the response in PZC. The longer duration studies in our review did not include intermediate measurements, so it was not possible to compare intermediate results with the results of shorter duration studies. However, since fortified foods are intended to target regularly consumed foods, without the need to change consumer dietary patterns, reduction in participant adherence may be less likely in a fortification program compared with a research setting.

Subgroup analyses found no difference in effect by contribution to EAR for zinc – a greater contribution to EAR did not lead to greater PZC increase or effects on prevalence of zinc deficiency, but this does not mean that greater zinc doses did not have greater effects at the individual level within a study. The limitations of study-level rather than individual-level classifications, e.g., baseline zinc deficiency, age, and baseline stunting status, and the use of categorical variables for zinc dose and duration, are a shortcoming of subgroup analyses compared with linear metaregression. Additionally, any subgroup interpretations should be considered cautiously, and the high number of outcomes and subgroup analyses included in this review increases the likelihood of a statistically significant false-positive, which could explain contradictory or unexpected findings.

The results of meta-analyses for FAZ and TAZ were in line with the PZC and zinc deficiency meta-analyses. Studies providing a zinc dose greater than 50% of the EAR had a lower FAZ but greater TAZ, which is consistent with studies of zinc supplementation (48). A significant positive effect was seen with hair and urine zinc, but only 2 studies presented these outcomes, 1 of which was considered of Poor quality, undermining confidence in these results. Other zinc-related biomarkers, including plasma fatty acid concentrations and immune system biomarkers, were too varied in measurement and/or confounded by coadministration of PUFAs to permit conclusions on effect of zinc fortification. There were no studies assessing comet assays for DNA strand breaks.

Although the present review expanded the body of information for anthropometry, cognition, and morbidity outcomes, the available body of evidence remains small. For these outcomes, cofortification with other micronutrients undermines the ability to attribute any effects specifically to the provision of zinc. Results from child anthropometry meta-analyses largely found no effect, except for a slight increase in weight. Ten out of 13 studies included in our review were conducted in SAC (5–12 y; only data for children under 10 was eligible for anthropometry outcomes); any potential effect of zinc fortification on growth is difficult to interpret considering the larger sample sizes necessary to detect differences in anthropometry in older children (due to low growth velocity). The duration of 7/13 studies with anthropometry outcomes was also <1 y; for older children, the period of observation may have been too short to allow detection of a growth effect. Although there were a limited number of studies included in the diarrhea and fever episodes meta-analyses (3 and 2, respectively), the statistically significant reduction in diarrhea and fever episodes is promising. Although the impact on diarrhea incidence cannot be attributed specifically to zinc because of cofortification with other nutrients, zinc is the 1 micronutrient whose supplementation is consistently linked with reduced incidence and duration of acute diarrhea in supplementation trials (49–52). From the small number of studies included in the meta-analysis, there may be a slight improvement in short-term auditory memory (as measured by WISC's digit span forward test); however, it's not clear whether this increase is cognitively meaningful or attributable specifically to zinc.

This review found an increase in iron status, as measured by plasma/serum ferritin, when foods were only fortified with zinc and no positive or negative effect when the food was cofortified with zinc and iron. There is evidence that when zinc and iron are provided simultaneously as aqueous solutions or in supplemental tablets in zinc-to-iron ratios greater than 2:1, zinc will inhibit iron uptake and vice versa (53, 54), although inhibition is generally not observed when both minerals are provided with food. However, interaction with iron may differ by zinc compound (55), zinc/iron molar ratios (54), and fortification vehicles (53). In 2 of the 3 studies (all in wheat flour) included for serum ferritin outcomes in this review, zinc sulfate was the compound used in fortification; zinc acetate and zinc oxide were used in the other 2 studies.

High daily intakes of supplemental zinc have been shown to block the intestinal absorption of copper (56), but little is known regarding zinc and copper interactions at the lower doses that are provided with food fortification. Although there were a limited number of studies that assessed plasma/serum copper concentrations after fortification with MMN + zinc, our review supports Das et al. (7) and Shah et al.’s (9) findings, which suggest there is no positive or negative effect on serum copper concentrations.

### Quality of evidence in the review

#### PZC, prevalence of zinc deficiency, and other zinc-related biomarkers (FAZ, TAZ, hair, and urine zinc concentrations)

The quality of the evidence for both PZC and prevalence of zinc deficiency was low, indicating that further research could possibly change the magnitude of the estimate and have an important impact on the CI of the effect. PZC and prevalence of zinc deficiency were downgraded for inconsistency, suggesting that although consuming zinc-fortified foods can positively affect these outcomes, there are other factors unidentified in this review that can affect the outcomes. The very low-quality evidence for FAZ, TAZ, hair, and urine zinc concentrations means that any estimate of effect is very uncertain. These outcomes were downgraded for inconsistency and imprecision, suggesting that unidentified factors were affecting the estimate *and* magnitude of effect. Factors known to affect zinc absorption include phytates, gastrointestinal and metabolic disorders, and hemoglobinopathies (1). Genetic polymorphisms may also have a role in influencing PZC and zinc-related outcomes (57); 1 study found that 20% of the variation in PZC in Australian adult twins was due to genetic factors (58). As PZC is homeostatically controlled, varying zinc intake (outside of the fortified food) and inhibitors to absorption (e.g., phytates, heat-derived zinc-binding ligands) in participants' diets may have also contributed to heterogeneity between studies.

#### Anthropometry

The strongest quality of evidence for any anthropometric outcome was that MMN + zinc fortification had no effect on MUAC and wasting. The evidence for both outcomes was classified as moderate-quality evidence, suggesting that additional research may change the estimate of effect. The remaining outcomes were considered low quality (weight, height, underweight, prevalence of underweight) or very low quality (stunting, prevalence of stunting, prevalence of wasting). Evidence for all of the anthropometric outcomes was downgraded based on indirectness (indirect intervention due to the inclusion of MMN); additional research with the ability to isolate the effect from zinc would improve our confidence in the estimates of this and other functional effects. However, considering that anthropometry could be influenced by many factors other than micronutrient intake, focused, well-designed research is necessary to improve our confidence in any estimates of specific effects attributable to zinc fortification.

#### Cognition

The positive effect on digit span forward and no effect on digit span backward were considered moderate-quality evidence; although there were just a small number of studies with cognition outcomes that could be pooled, there was low heterogeneity between the studies. However, cognition outcomes were downgraded based on indirectness (indirect intervention due to the inclusion of MMN), pointing to the need for study designs that isolate for the effect of zinc in fortification.

#### Morbidity

Evidence for no effect on nausea or skin rashes was moderate quality; otherwise, evidence for other outcomes was low quality (diarrhea, vomiting, and fever episodes) or very low (stomach pain, URTI, LRTI). Like anthropometry and cognition, evidence for all of the morbidity outcomes was downgraded based on indirectness (indirect intervention due to the inclusion of MMN); additional research with the ability to isolate the effect of zinc would improve our confidence in the estimates of effect on morbidity outcomes. A clear shortcoming, however, was inconsistent measurements and reporting that did not allow for pooling results even though many more studies reported morbidity outcomes.

#### Iron/copper interaction

The increase in serum ferritin from zinc fortification was of moderate quality and downgraded for imprecision. Our finding that zinc fortification had no effect on copper concentrations was of very low quality due to risk of bias and imprecision.

### Strengths and limitations of this review

Inclusion eligibility in this review was broad, including multiple biomarkers and functional outcomes, thus providing a comprehensive update of zinc-related outcomes. Although the results for some of the outcomes included in this review lacked any information (comet assay) or were difficult to interpret due to cofortification (e.g., with PUFAs) or inconsistent assessment methodologies across studies (e.g., immune function), the inclusion of these outcomes in the review provides information on the current state of available evidence.

A limitation of this review was that we did not have capacity to include non-English language studies. However, Shah et al. (9) were able to share the translated manuscript of 1 of the non-English studies included in their review (59). Although we screened Shah et al.’s (9) list of excluded records and did not find any other relevant non-English language studies, given the differing eligibility criteria between the reviews, we may have missed additional non-English records that were published after Shah et al.’s (9) search was conducted.

### Implications for fortification programs considering the inclusion of zinc

The results of this review provide further justification for the inclusion of zinc in maize and wheat flour fortification programs as an effective and safe intervention, for which the WHO has existing recommendations for zinc concentrations and compounds to add (60, 61). For other foods – particularly rice, milk, and certain condiments, where there are no food-specific WHO recommendations for zinc fortification concentrations – our review suggests that adding zinc to these foods could increase PZC as well. In countries with populations at risk of inadequate zinc intake, and where these foods are widely consumed in adequate amounts and are industrially processed, fortification program managers should consider adding zinc to these foods as a complementary intervention alongside dietary diversification and modification, home-fortification, supplementation, and other approaches, to improve dietary zinc intake and biochemical zinc status.

Based on this review, there is no evidence of an impact of zinc fortification on functional outcomes such as stunting, which has been linked to zinc in supplementation trials. Zinc fortification in combination with other micronutrients did however, lead to a reduced incidence of acute diarrhea among children, but this was based on data from just 3 efficacy studies. Decision makers should be informed that evidence to date on health impacts of zinc fortification, although promising, is based on a small number of studies. When evaluating the inclusion of zinc in fortification programs, program managers should focus on PZC and preventing biochemical evidence of zinc deficiency, using recommended assessment methods (62, 63).

### Implications for further research

It remains to be confirmed whether zinc fortification can translate into a significant effect in morbidity outcomes, including diarrhea and fever. Researchers or fortification program evaluators should consider consistency in measuring and reporting morbidity outcomes (e.g., reporting prevalence, episodes, or days with disease); this would increase the number of studies eligible for inclusion in meta-analyses and confidence in results and interpretation. Better designed trials, of longer duration and in appropriate age groups, to specifically assess changes in anthropometry are also needed. There were confounded, limited, or inconsistently evaluated results for novel zinc and immune system biomarkers – future research on the potential effect of zinc fortification on these outcomes would fill an existing gap, but would first benefit from a research agenda that identifies common methods and indicators for evaluation.

## Conclusions

This review considered the impacts of zinc fortification, delivered alone or in the context of MMN fortification, which reflects current practices in fortification programs. In the populations included in this review, fortifying foods with zinc increased PZC and reduced the prevalence of zinc deficiency, regardless of the composition of the fortification premix or the vehicles that were fortified. Where populations are at risk of inadequate zinc intake, zinc fortification of foods that are widely consumed in adequate amounts should be considered as part of a comprehensive nutrition strategy to improve zinc intakes and status.

**TABLE 1:** Summary of findings – fortified food with zinc (alone or cofortified with multiple micronutrients) compared with control (nonfortified, fortified but without zinc, or no food)

Patient or population: General population					
Setting: Global					
Intervention: Fortified food with zinc					
Comparison: Nonfortified food, fortified but without zinc, or no food					
	Anticipated absolute effects^1^ (95% CI)				Certainty of the evidence (GRADE)^2^
Outcomes	*Risk with control*	*Risk with fortified food with zinc*	Relative effect (95% CI)	Participants, *n* (studies)	
PZC (μg/dL) efficacy studies	—	MD 4.58 μg/dL higher (2.62–6.75 higher)	—	6173 (27 RCTs)	Low^3,4^
PZC (μg/dL) effectiveness studies	—	MD 6.28 μg/dL higher (3.73–8.84 higher)	—	6902 (13 observational studies)	Low^3^
Prevalence of zinc deficiency: efficacy studies	404 per 1000	340 per 1000 (289–394)	OR 0.76 (0.60 to 0.96)	3562 (10 RCTs)	Very low^5,6^
Prevalence of zinc deficiency: effectiveness studies	502 per 1000	312 per 1000 (238–392)	OR 0.45 (0.31 to 0.64)	7780 (10 observational studies)	Low^3,7^
Weight (kg): efficacy studies	—	MD 0.43 kg higher (0.11 higher to 0.75 higher)	—	1410 (11 RCTs)	Low^5,8^
Diarrhea (episodes): efficacy studies	—	—	RR 0.79 (0.68 to 0.92)	1364 (3 RCTs)	Low^8,9^

GRADE, Grading of Recommendations, Assessment, Development, and Evaluations; MD, mean difference; PZC, plasma/zinc concentrations; RCT, randomized controlled trials; RR, rate ratio.

1 The risk in the intervention group (and its 95% CI) is based on the assumed risk in the comparison group and the relative effect of the intervention (and its 95% CI).

2 High certainty: we are very confident that the true effect lies close to that of the estimate of the effect; moderate certainty: we are moderately confident in the effect estimate: the true effect is likely to be close to the estimate of the effect, but there is a possibility that it is substantially different; low certainty: our confidence in the effect estimate is limited: the true effect may be substantially different from the estimate of the effect; very low certainty: we have very little confidence in the effect estimate: the true effect is likely to be substantially different from the estimate of effect.

3 *I^2^* = 75 to 100%, *P* <0.05: considerable heterogeneity.

4 Asymmetrical funnel plot.

5 *I^2^* = 40 to 74% and *P* ≤0.05.

6 24 efficacy articles in this review included serum/plasma zinc concentrations. However, only 9 reported prevalence of zinc deficiency.

7 12 effectiveness articles in this review reported serum/plasma zinc concentrations. However, only 8 reported prevalence of zinc deficiency.

8 Indirect intervention: studies included other nutrients, not just zinc.

9 Wide CIs include appreciable benefit (0.75) and/or harm (1.25).

## Supplementary Material

nmab065_Supplemental_Files

## Notes

This work was supported by the Bill & Melinda Gates Foundation (INV-007767).

Author disclosures: The authors report no conflicts of interest.

Supplemental Figures 1–39, Supplemental Tables 1–15, and Supplemental Method are available from the “Supplementary data” link in the online posting of the article and from the same link in the online table of contents at https://academic.oup.com/advances/.

Abbreviations used: EAR, estimated average requirement; FAZ, fractional zinc absorption; GRADE, Grading of Recommendations Assessment, Development, and Evaluation; HAZ, height-for-age z-score; IZiNCG, International Zinc Nutrition Consultative Group; LRTI, lower respiratory tract infection; MeSH, Medical Subject Headings; MMN, multiple micronutrients; MUAC, midupper arm circumference; NHLBI, National Heart, Lung, and Blood Institute; PRISMA, Preferred Reporting Items for Systematic Reviews and Meta-Analyses; PSAC, preschool-age children; PZC, plasma/serum zinc concentrations; SAC, school-age children; TAZ, total absorbed zinc; URTI, upper respiratory tract infection; WAZ, weight-for-age z-score; WHZ, weight-for-height z-score; WISC, Wechsler Intelligence Scale for Children; WRA, women of reproductive age.
