# Supplementary material for: Effects of Foods Fortified with Zinc, Alone or Cofortified with Multiple Micronutrients, on Health and Functional Outcomes: A Systematic Review and Meta-Analysis
Source: Adv Nutr. 2021 Jun 24;12(5):1821–37. doi: 10.1093/advances/nmab065 (PMC8483949; doi:10.1093/advances/nmab065)
Supplement: nmab065_Supplemental_Files [file nmab065_supplemental_files.zip › Supplemental Document 1.docx]

**PROPOSED AUTHORS**

Becky Tsang

Independent consultant

IZiNCG Fortification Task Force

Erin Holsted

Independent consultant

Emory University, MPH candidate

Mari Manger

IZiNCG

Christine McDonald

IZiNCG

Children’s Hospital Oakland Research Institute

Mduduzi Mbuya

Global Alliance for Improved Nutrition

IZiNCG Fortification Task Force

Frederick Grant

Helen Keller International

IZiNCG Fortification Task Force

Ken Brown

UC Davis

IZiNCG Fortification Task Force

Robert Black

Johns Hopkins School of Public Health

IZiNCG Fortification Task Force

Laura Rowe

Food Fortification Initiative

IZiNCG Fortification Task Force

**BACKGROUND**

IZiNCG is seeking evidence to inform and update its position and recommendations on food fortification with zinc. A systematic review of studies that have evaluated efficacy and effectiveness outcomes of zinc fortified foods will be conducted alongside a series of key informant interviews. IZiNCG has formed a multi-agency Task Force to guide the systematic review process and interpret the results.

*Objectives:*

Review published literature to identify and describe the bioavailability, efficacy and effectiveness of any foods fortified with zinc. Through the identified methods below, the review will aim to answer the following questions:

- What is the impact of zinc fortification on defined zinc-related biochemical and functional health outcomes and adverse effects??
- What is the adequate dosage and duration of exposure to zinc-fortified food that is required to demonstrate an effect on defined zinc-related biochemical and functional outcomes?
- What proportion reduction in zinc deficiency can we expect to see as a result of zinc fortification, and which groups may require additional/complementary interventions? This should also include the reduction in the proportion of the population with inadequate dietary zinc intake.

**METHODS**

Criteria for considering studies for this review

*Types of studies for inclusion*

- Placebo controlled trials
  - Randomized controlled trials
  - Quasi-randomized controlled trials
  - Non-randomized controlled trials
  - Zinc absorption studies (e.g., balance studies, stable isotope bioavailability studies)
- Non-placebo controlled trials
  - Randomized trials
  - Quasi-randomized trials
  - Zinc absorption studies (e.g., balance studies, stable isotope bioavailability studies)

*Types of studies that will be excluded*

- Bio-accessibility studies using *in vitro* methods to assess zinc availability in model digestive systems.

*Classification into efficacy or effectiveness studies*

- Efficacy studies
  - Controlled or non-placebo controlled trials where participants are known to consume food fortified with zinc under carefully dosed and measured settings:
    - Fortified food should be prepared by the study investigators
    - The amount of fortified food consumed is known
- Effectiveness studies
  - Controlled or non-placebo controlled trials where participants or households are provided food fortified with zinc, but investigators may not have control over the following:
    - How participants/households store, prepare, and cook the food
    - Who consumes the food, regardless of the target population
    - How much of the food is consumed/wasted
  - Controlled or non-controlled cohort or population-based studies where participants are unknown to have access or to have consumed a food fortified with zinc, but mandatory legislation of a food fortified with zinc is in place and a pre-post evaluation of the fortification program is conducted

*Types of participants for inclusion*

For studies in humans: male or female, of any age, regardless of baseline zinc status

*Types of participants for exclusion*

- For studies in populations selected for pre-existing health conditions (except for anemia prevalence, zinc deficiency, stunting)
- Participants receiving a zinc-fortified food product for therapeutic use:
  - Participants in supplementary feeding programs for the prevention or treatment of moderate and severe acute malnutrition
  - HIV patients
  - Hospitalized, institutionalized, or long-term care patients

*Types of interventions for inclusion*

- Post-harvest fortification of any food (inclusive of condiments and beverages) for human consumption with zinc
  - Studies where zinc is the only fortificant or studies of co-fortification where the effect of zinc can be isolated.
  - Foods co-fortified with multiple micronutrients in addition to zinc and there is no control or comparison group that allows for the isolation of the effect of zinc.
  - Where a food is fortified with zinc and delivered with a co-intervention (e.g. de-worming) and there is no control or comparison group that allows for the isolation of the effect of zinc.

*Types of interventions for exclusion*

- Bio-fortification of zinc is not considered within scope of this review.
- Zinc supplementation, including point-of-use fortification with micronutrient powders or lipid-based foods, infant formula, or tablet/pill supplementation.
- Non-centrally produced foods (or without plausible likelihood to be centrally produced)

*Other inclusion/exclusion criteria*

- English language literature
- No time limitation
- Studies in humans only

Types of outcome measures for extraction (both positive and adverse effects)

- Zinc biomarkers (serum or plasma zinc, hair, nail, urine), continuous and deficiency as defined by IZiNCG (1), other deficiency as defined by trial authors
- Child anthropometry (height, weight, stunting, wasting, underweight, mid-upper arm circumference)
- Morbidity and mortality, e.g. diarrhea, acute respiratory infections, vomiting, hospitalizations, all-cause mortality and others as defined by trial authors
- Immune function biomarkers (as defined by trial authors)
- Comet assay (i.e. DNA strand breaks)
- Plasma fatty acid concentrations
- Fractional absorption of zinc and total absorbed zinc
- Interaction with iron absorption (as measured by serum ferritin)
- Interaction with copper absorption (as measured by copper biomarkers, defined by trial authors)
- Change in consumption and coverage of the zinc-fortified food (effectiveness studies only)

Search strategy for the identification of studies

*Electronic search:*

We will search the following databases for relevant studies:

- PubMed
- Agricultural & Environmental Science Collection
- Agricola
- CAB Abstracts
- Embase
- Scopus
- Web of Science

We will not conduct hand searching of references in reviews found in the results (except for known previous zinc fortification reviews, i.e., Hess and Brown 2009, Das et al 2013, Shah et al 2016.

*Search string:*

The following search string will be in PubMed to identify studies inclusive of food fortification with zinc, and modified as necessary for the other databases listed in the electronic search:

(fortify OR fortifies OR fortified OR fortifying OR fortification OR enrich OR enriches OR

enriched OR enriching OR enrichment OR enriched food OR enriched foods OR fortified

food OR fortified foods)

*Grey literature search resources:*

- OpenGrey
- ProQuest Dissertations and Theses Global
- Known literature from the Task Force

Data collection and analysis

Search results will be managed and de-duplicated in *Endnote* software^[[1]](#footnote-1)^.

*Selection of studies*

Two review authors will independently screen all titles and abstracts using *Covidence* software^[[2]](#footnote-2)^. When a title or abstract cannot be rejected with certainty, we will obtain the full text of the article for further evaluation. BLT and the EH will independently screen full-text articles for final assessment of eligibility. If full articles cannot be obtained, we will attempt to contact the authors to obtain further details of the study. Failing this, we will classify studies as ’awaiting assessment’ until further information is published or made available to us. Disagreements at any stage of the eligibility assessment process will be resolved through discussion and consultation with a third author (TBD) where necessary.

*Data extraction and management*

We will extract data from studies using a digital extraction form in Microsoft Excel, designed for this review. We will first pilot the form on a small number of study reports, and modify it if necessary. Two review authors will independently extract data from all eligible studies and check each other’s work for accuracy. Study data will be entered into Review Manager 5 (RevMan) software^[[3]](#footnote-3)^. Disagreements at any stage of the data extraction process will be resolved through discussion and consultation with a third author (KB), where necessary.

Data will be collected across the following domains.

- Study design:
  - Unit of randomization
  - Method of allocation
  - Masking of participants, personnel, and outcome assessors
- Study population:
  - Sample size
  - Procedures for selecting participants
  - Exclusion of participants and losses to follow-up
  - Location of the study
  - Age, sex, physiological status
  - Baseline zinc status
  - Inclusion and exclusion criteria, as described above
- Zinc biomarker assay method
  - Inductively Coupled Plasma - Mass Spectrometry (ICP-MS)
  - Inductively Coupled Plasma - Optical Emission Spectrophotometers (ICP-OES)
  - Atomic Absorption Spectroscopy (AAS)
- Intervention/exposure
  - Zinc dose (e.g. consumption), coverage, and frequency
  - Zinc compound
  - Duration of the intervention
  - Co-fortification of other nutrients
  - Co-interventions other than fortification
  - Comparison group
- Outcomes
  - As described above.

*Assessment of risk of bias in included studies*

Two review authors (BLT and MM) will independently assess risk of bias of the included studies. We will use the standard Cochrane ‘Risk of bias’ tool to assess the following domains below for all efficacy studies^[[4]](#footnote-4)^ (Note Addendum 2/07/2020 – the Cochrane tool was replaced by the National Heart Lung and Blood Institute tools). We will resolve any disagreement by discussion or by involving a third reviewer (CM). The following domains will be included:

- Random sequence generation (checking for selection bias)
- Allocation concealment (checking for possible selection bias)
- Blinding of participants and personnel (checking for possible performance bias
- Blinding of outcome assessment (checking for possible detection bias)
- Incomplete outcome data (checking for possible attrition bias through withdrawals, dropouts, protocol deviations
- Reporting bias
- Other sources of bias (to be defined by task force)

Overall risk of bias will be summarized at two levels: within studies (across domains) and across studies (for each outcome). Studies judged to be at high risk of bias will be those with high or unclear risk of bias in the following domains: allocation concealment, similarity of baseline outcome measurements, and completeness of outcome data.

If there is insufficient information in study reports for us to be able to assess risk of bias, studies will await assessment until further information is published, or made available to us. For each individual outcome, we will assess the quality of the evidence using the Grades of Recommendations, Assessment, Development and Evaluation (GRADE) approach^[[5]](#footnote-5)^, which involves consideration of within-study risk of bias (methodological quality), directness of evidence, heterogeneity, precision of effect estimates, and risk of publication bias. We will express the results as one of four levels of quality (high, moderate, low or very low). We will limit this assessment to the trials included in this review only (i.e. we will not consider indirect evidence).

We will set out the main findings in ’Summary of findings’ (SoF) tables prepared using GRADE profiler software^[[6]](#footnote-6)^. We will list the outcomes for each comparison with estimates of relative effects along with the number of participants and studies contributing data for those outcomes.

*Dealing with missing data*

If there is further data necessary to include or analyze a study, we will contact authors with a maximum of two attempts.

*Data synthesis/Meta-analysis*

We will carry out a meta-analysis to provide an overall estimate of treatment effect when more than one study examines the same intervention, if the studies use similar methods and measure the same outcome in similar ways. We will combine results from randomized controlled trials and quasi-randomized trials only. We will not pool any of the other study designs together (See Addendum, 6/01/2020; efficacy and effectiveness studies were pooled separately).

We will carry out statistical analysis using RevMan. We will use a random-effects meta-analysis for combining data, as we anticipate that there may be natural heterogeneity between studies attributable to the different doses, durations, populations, and implementation/delivery strategies. For continuous variables, we will use the inverse variance method, while for dichotomous variables we will use the method proposed by Mantel and Haenszel (See Addendum, 6/01/2020; as the data were entered in RevMan, Mantel Haenszel option was not a selectable option for analysis).

For non-randomized controlled trials, where results have been adjusted to take account of possible confounding factors, we will use the generic inverse variance method in RevMan to carry out any meta-analysis (if both adjusted and non-adjusted figures are provided, we will carry out a sensitivity analysis using the unadjusted figures to examine any possible impact on the estimate of treatment effect)^[[7]](#footnote-7)^.

We will explore heterogeneity according to the subgroups identified below in the next section. In addition, we will use narrative synthesis, guided by the data extraction form in terms of the ways in which studies may be grouped and summarized in this review, to explore intervention implementation (using information about resource use and findings from process evaluations).

*Subgroup analysis*

Where data are available, we will conduct the following subgroup analyses:

- Study quality (Good, Fair, Poor)
- Food vehicle (cereals vs. beverages vs. condiments)
- Co-fortification with iron
  - Iron status (serum ferritin)
  - Copper status (as defined by trial authors)
- Daily dose (% EAR, median)
- Duration (above and below median)
- Baseline zinc status (deficient versus non-deficient)
- Age and sex (under 2 years of age, PSAC (under 5), SAC (5-12), WRA (aged 12-49), men aged 12-49, individuals aged 50+)
- Stunting status (population prevalence of stunting > 20 % stunted vs. < 20% stunted)
- Region, as defined by UNICEF
- Income status (World Bank classification)

Ethics review:

Protocol will be submitted to Emory and Children’s Hospital Oakland Research Institute’s IRB for determination if an ethics review of the project needs to be completed.

Author roles and responsibilities:

Proposed co-authors: Erin Holsted (EH), Becky L Tsang (BLT), Mari Manger (MSM), Christine McDonald (CM), Mduduzi Mbuya (MM), Fred Grant (FG), Ken Brown (KB), Robert Black (RB), Laura Rowe (LR) and proposed roles are:

- BLT will supervise the student and BLT will be the lead author
- EH will conduct the search, remove duplicates using Endnote, and use *Covidence* to organize title and abstract review.
- EH and BLT will review titles and abstracts and full texts eligibility in duplicate; a member of the task force will serve as the third-party adjudicator.
- EH and BLT will extract data from eligible papers and review each other’s work.
- BLT and pre-identified members of the task force will conduct the risk of bias, grading of evidence, and meta-analysis. EH may potentially also be involved depending on her proficiency and availability.
- EH, BLT, MM, CM, MM, FG, KB, RB, and LR will contribute to writing and reviewing of the manuscript.

Deliverable:

A systematic review manuscript, with accompanying tables, and PRISMA flow-chart figure, full PDF files of included studies, and Endnote library. Dissemination activities include a conference presentation and webinar (TBC).

Addendums to clarify protocol:

Addendum (12/11/19):

- Studies involving complementary foods will be included if the food in the study aligns with the following criteria:
  - All ingredients are clearly stated
  - Contains a single cereal ingredient
  - Must not contain legumes
- Studies involving rice fortification
  - Methodology to be included:
    - Coating
    - Extrusion
  - Methodology to be excluded:
    - Exogenously added zinc
- Studies involving fortification of water
  - Methodology to be excluded:
    - Point-of-use filtration system (glassy zinc phosphate–based plates)

Addendum (2/07/20)

Switched to using the National Heart Lung and Blood Institute’s (NHLBI) quality assessment tools – advantage being that the NHLBI tools are already adapted to different study designs. The Cochrane RoB tool is best suited for randomized, controlled trials.

Addendum (2/18/20):

- For iron Interaction:
  - Studies measuring interaction with iron absorption will be included if measured by serum ferritin
  - We will not include studies that have multiple-micronutrient fortified food compared to no fortification because we cannot isolate the zinc/iron interaction
  - Include studies that compare:
    - Zinc only vs iron+zinc fortified food
    - Iron only vs iron+zinc fortified food

Addendum (2/24/20):

- For child anthropometry:
  - We will extract anthropometric data for children ages 0-10
  - The World Health Organization’s definition of a child is as follows: “A child is a person 19 years or younger unless national law defines a person to be an adult at an earlier age. However, in these guidelines when a person falls into the 10 to 19 age category they are referred to as an adolescent”

Addendum (4/20/20):

- For copper interaction
  - Include studies that compare:
    - Zinc only vs copper+zinc fortified food
    - Copper only vs copper+zinc fortified food
    - Zinc only vs. no zinc fortified food
- Risk of Bias for stable isotope studies will only be conducted for placebo-controlled studies or where studies compare multiple zinc fortification levels. All other stable isotope studies comparing other zinc fortification characteristics without a control arm (e.g. comparing zinc compounds) will be included only descriptively.

Addendum (6/01/20)

- Study pooling for meta-analysis
  - Studies were pooled by efficacy study design and effectiveness study design
  - Both dichotomous and continuous variables used inverse variance method; this was the only analysis method offered in RevMan since the effect estimate was calculated and entered directly into RevMan.

Addendum (6/25/20)

- Two studies (Hansen, Muthayya) used low-zinc fortification arms as controls (<1.5 mg/d); these were included in the review.

**References:**

1. IZiNCG. Assessing population zinc status with serum zinc concentration [Internet]. 2012. Report No.: 02. Available from: https://static1.squarespace.com/static/56424f6ce4b0552eb7fdc4e8/t/5774378f414fb5410541b748/1467234199261/IZiNCG_TechBrief2_2012-3.pdf

1. Endnote X9 software, Clarivate Analytics, Available at https://endnote.com/product-details/ [↑](#footnote-ref-1)
2. Covidence systematic review software, Veritas Health Innovation, Melbourne, Australia. Available at www.covidence.org [↑](#footnote-ref-2)
3. Review Manager (RevMan) [Computer program]. Version 5.3. Copenhagen: The Nordic Cochrane Centre, The Cochrane Collaboration, 2014. [↑](#footnote-ref-3)
4. Higgins JPT, Green S (editors). Cochrane Handbook for Systematic Reviews of Interventions Version 5.0.1 [updated March 2011]. The Cochrane Collaboration, 2011. Available from www.cochrane-handbook.org. [↑](#footnote-ref-4)
5. Balshem H, Helfand M, Schünemann HJ, Oxman AD, Kunz R, Brozek J, et al. GRADE guidelines 3: rating the quality of evidence. *Journal of Clinical Epidemiology* 2011; **64**(4):401–6. [↑](#footnote-ref-5)
6. GRADEpro GDT: GRADEpro Guideline Development Tool [Software]. McMaster University, 2015 (developed by Evidence Prime, Inc.). Available from gradepro.org. [↑](#footnote-ref-6)
7. Adjusted estimates were not typically provided; where adjusted estimates were provided, they were used in analysis. No sensitivity analysis was conducted. [↑](#footnote-ref-7)
