# Supplementary material for: Effects of Foods Fortified with Zinc, Alone or Cofortified with Multiple Micronutrients, on Health and Functional Outcomes: A Systematic Review and Meta-Analysis
Source: Adv Nutr. 2021 Jun 24;12(5):1821–37. doi: 10.1093/advances/nmab065 (PMC8483949; doi:10.1093/advances/nmab065)
Supplement: nmab065_Supplemental_Files [file nmab065_supplemental_files.zip › Supplemental figure 18.pdf]

Tsang BL, et al. “Effects of foods fortified with zinc, alone or co-fortified with multiple micronutrients, on health and functional outcomes: a systematic review and meta-analysis”
Online Supplementary Material

Supplemental figure 18.Height duration

	Fortified food			Control				Mean Difference
**Study or Subgroup**	**Mean [cm]**	**SD [cm]**	**Total**	**Mean [cm]**	**SD [cm]**	**Total**	**Weight**	**IV, Random, 95% CI**
**7.4.1 <6 months**								
Petrova 2019	133.18	6.47	19	135.67	8.01	15	1.6%	−2.49 [−7.48, 2.50]
López de Romaña 2005 (1)	91.7	4.6	12	93.5	3.7	6	2.5%	−1.80 [−5.74, 2.14]
Saldamli 1996	132.3	10.2	12	133.9	11.1	12	0.6%	−1.60 [−10.13, 6.93]
Thankachan 2013	128	1	120	129	1	123	21.8%	−1.00 [−1.25, −0.75]
Trinidad 2015 (2)	114.4	4.54	42	114.5	4.43	21	5.8%	−0.10 [−2.44, 2.24]
Angeles−Agdeppa 2011	123	7.4	46	123	5.9	43	4.5%	0.00 [−2.77, 2.77]
Trinidad 2015 (3)	115	4.54	42	114.5	4.43	21	5.8%	0.50 [−1.84, 2.84]
López de Romaña 2005 (4)	94.7	5.5	10	93.5	3.7	6	1.9%	1.20 [−3.32, 5.72]
**Subtotal (95% CI)**			**303**			**247**	**44.5%**	**−0.97 [−1.21, −0.72]**
Heterogeneity: $Tau^2 = 0.00$; $Chi^2 = 4.01$, df = 7 (P = 0.78); $I^2 = 0\%$								
Test for overall effect: Z = 7.69 (P < 0.00001)								
**7.4.2 6+ months**								
Do 2009	120.9	5.2	150	121	5	151	13.3%	−0.10 [−1.25, 1.05]
Bardosono 2009	3.03	1	121	2.84	0.88	124	21.9%	0.19 [−0.05, 0.43]
Muthayya 2009	129.2	8	271	129	8.1	279	11.6%	0.20 [−1.15, 1.55]
Manger 2008	131.3	10.77	280	131.1	10.31	283	8.7%	0.20 [−1.54, 1.94]
**Subtotal (95% CI)**			**822**			**837**	**55.5%**	**0.18 [−0.05, 0.41]**
Heterogeneity: $Tau^2 = 0.00$; $Chi^2 = 0.23$, df = 3 (P = 0.97); $I^2 = 0\%$								
Test for overall effect: Z = 1.55 (P = 0.12)								
**Total (95% CI)**			**1125**			**1084**	**100.0%**	**−0.20 [−0.85, 0.46]**
Heterogeneity: $Tau^2 = 0.50$; $Chi^2 = 49.33$, df = 11 (P < 0.00001); $I^2 = 78\%$								
Test for overall effect: Z = 0.59 (P = 0.56)								
Test for subgroup differences: $Chi^2 = 45.09$, df = 1 (P < 0.00001), $I^2 = 97.8\%$								

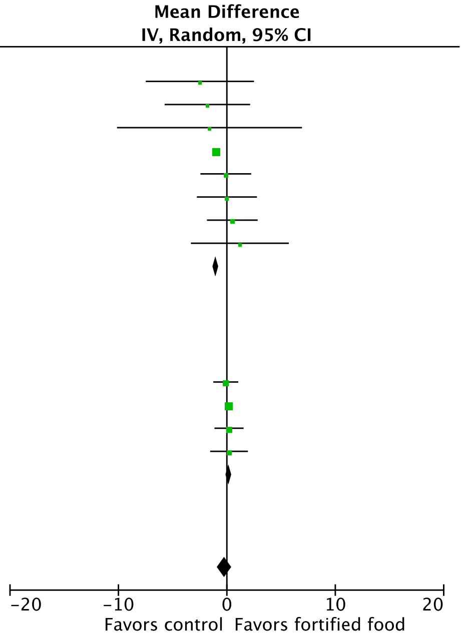

Footnotes
(1) ZN0 v ZN9
(2) 1 glass of fortified milk v. water
(3) 2 glasses of fortified milk v. water
(4) ZN0 v ZN3
