# Supplementary material for: Effects of Foods Fortified with Zinc, Alone or Cofortified with Multiple Micronutrients, on Health and Functional Outcomes: A Systematic Review and Meta-Analysis
Source: Adv Nutr. 2021 Jun 24;12(5):1821–37. doi: 10.1093/advances/nmab065 (PMC8483949; doi:10.1093/advances/nmab065)
Supplement: nmab065_Supplemental_Files [file nmab065_supplemental_files.zip › Supplemental figure 2.pdf]

Supplemental figure 2.efficacy PZC duration subgroup

Study or Subgroup	Fortified food Mean [μg/dL]	Fortified food SD [μg/dL]	Fortified food Total	Control Mean [μg/dL]	Control SD [μg/dL]	Control Total	Weight	Mean Difference IV, Random, 95% CI
**2.4.3 <6 months**								
Angeles-Agdeppa 2017 (1)	66	14.967	56	68	14.97	20	2.7%	−2.00 [−9.64, 5.64]
López de Romaña 2005 (2)	76.2	8.2	10	77.6	12.3	6	1.9%	−1.40 [−12.48, 9.68]
Aaron 2011b (3)	1.96	8.64	33	3.07	8.64	16	3.4%	−1.11 [−6.27, 4.05]
Angeles-Agdeppa 2017 (4)	67	22.847	58	68	14.97	19	2.4%	−1.00 [−9.94, 7.94]
Aaron 2011b (5)	2.09	8.73	31	3.07	8.64	17	3.4%	−0.98 [−6.11, 4.15]
Thankachan 2013	130.2	28.5	115	129.4	26.8	120	2.9%	0.80 [−6.28, 7.88]
Pinkaew 2013	72.55	8.5	90	69.28	9.15	82	4.1%	3.27 [0.62, 5.92]
Hettiarachchi 2004 (6)	83.01	16.99	12	79.08	14.38	12	1.7%	3.93 [−8.66, 16.52]
Nga 2009	61.44	12.42	232	57.19	12.42	235	4.2%	4.25 [2.00, 6.50]
Hettiarachchi 2004 (7)	84.97	16.34	13	80.39	18.95	12	1.5%	4.58 [−9.34, 18.50]
López de Romaña 2005 (8)	82.4	9.9	12	77.6	12.3	6	1.9%	4.80 [−6.52, 16.12]
Angeles-Agdeppa 2017 (9)	73	22.45	56	68	14.97	19	2.4%	5.00 [−3.94, 13.94]
Abrams 2003	84.97	13.07	130	78.43	13.07	124	4.0%	6.54 [3.32, 9.76]
Badii 2011 (10)	78.73	8.1	25	67.73	2.17	12	3.9%	11.00 [7.60, 14.40]
Costarelli 2014	101.24	40.73	21	89.28	33.85	21	0.7%	11.96 [−10.69, 34.61]
Haibin 2001 (11)	100	55.56	10	82.35	41.83	9	0.2%	17.65 [−26.31, 61.61]
Kiliç 1998	81.57	9.08	12	62.94	3.4	12	3.3%	18.63 [13.14, 24.12]
Badii 2011 (12)	86.8	10.78	25	67.73	2.17	13	3.7%	19.07 [14.68, 23.46]
Angeles-Agdeppa 2011	116.3	26.1	46	96.6	22.7	43	2.1%	19.70 [9.55, 29.85]
Trinidad 2015 (13)	109	26.57	42	85.6	35.42	20	1.1%	23.40 [5.92, 40.88]
Haibin 2001 (14)	109.8	84.97	28	74.51	76.47	29	0.2%	35.29 [−6.72, 77.30]
Trinidad 2015 (15)	121.5	32.4	42	85.6	35.42	20	1.0%	35.90 [17.54, 54.26]
**Subtotal (95% CI)**			**1099**			**867**	**52.7%**	**7.24 [4.00, 10.48]**
Heterogeneity: Tau² = 36.61; Chi² = 117.47, df = 21 (P < 0.00001); I² = 82%								
Test for overall effect: Z = 4.38 (P < 0.0001)								
**2.4.4 6+ months**								
Ibnu 2019 (16)	53.73	8.41	30	57.52	9.43	30	3.6%	−3.79 [−8.31, 0.73]
Villalpando 2006	82.42	6.27	58	85.42	6.54	57	4.1%	−3.00 [−5.34, −0.66]
Sazawal 2010	61.4	26.8	233	63.4	29.4	232	3.5%	−2.00 [−7.11, 3.11]
Hieu 2012	71.24	16.99	101	70.59	16.99	95	3.6%	0.65 [−4.11, 5.41]
Bardosono 2009	−19.67	21.18	121	−20.39	24.31	124	3.3%	0.72 [−4.99, 6.43]
Sazawal 2013	9.44	28.56	278	8.1	26.21	293	3.6%	1.34 [−3.16, 5.84]
Winichagoon 2006	73.2	10.65	247	71.24	10.92	261	4.2%	1.96 [0.08, 3.84]
Wibowo 2016	51	8.14	46	48.8	8.6	58	4.0%	2.20 [−1.03, 5.43]
Do 2009	82.35	16.99	150	79.74	13.07	151	3.9%	2.61 [−0.82, 6.04]
Aaron 2011a	6.54	20.67	250	3.92	20.42	244	3.9%	2.62 [−1.00, 6.24]
Hyder 2007	88.65	31.98	433	85.4	29.74	470	3.8%	3.25 [−0.79, 7.29]
Ibnu 2019 (17)	66.59	6.76	32	59.06	7.81	32	3.9%	7.53 [3.95, 11.11]
Méndez 2012	116	26.9	39	98.5	26.6	47	1.9%	17.50 [6.14, 28.86]
Vinodkumar 2009	142.5	132	45	102.73	88.78	50	0.2%	39.77 [−5.98, 85.52]
**Subtotal (95% CI)**			**2063**			**2144**	**47.3%**	**1.72 [−0.26, 3.71]**
Heterogeneity: Tau² = 8.71; Chi² = 44.43, df = 13 (P < 0.0001); I² = 71%								
Test for overall effect: Z = 1.70 (P = 0.09)								
**Total (95% CI)**			**3162**			**3011**	**100.0%**	**4.68 [2.62, 6.75]**
Heterogeneity: Tau² = 25.25; Chi² = 204.42, df = 35 (P < 0.00001); I² = 83%								
Test for overall effect: Z = 4.45 (P < 0.00001)								
Test for subgroup differences: Chi² = 8.09, df = 1 (P = 0.004), I² = 87.6%								

Mean Difference
IV, Random, 95% CI

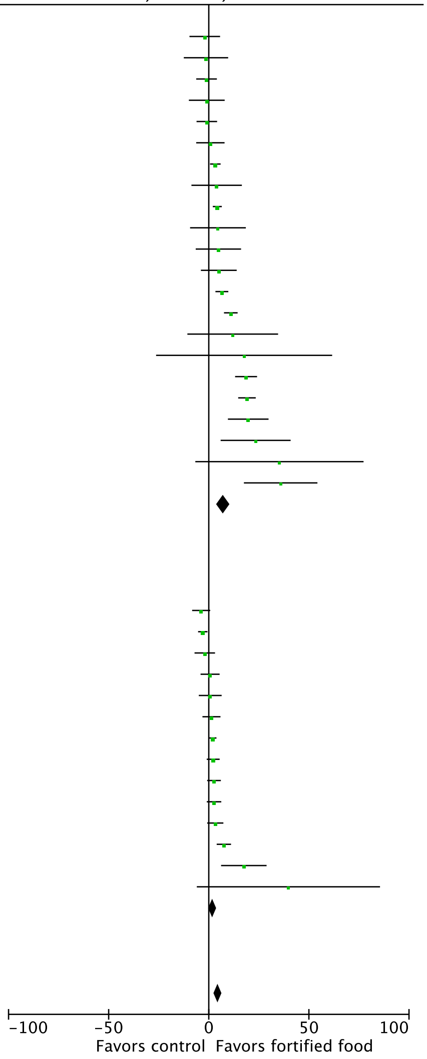

Footnotes
(1) Low (3 beverages/wk, ~2.4 mg/d on average)
(2) ZN3 v. ZN0
(3) 7.5 mg/d
(4) High (7 drinks/wk, 5.6 mg/d)
(5) 15 mg/d
(6) Na2EDTA+FeSO4+FA
(7) FeSO4+FA
(8) ZN9 v. ZN0
(9) Moderate (5 drinks/wk, ~4 mg/d on average)
(10) 100 mg/kg v. Control
(11) Zn+Ca+VD+Fe vs. Ca+VD+Fe
(12) 50 mg/kg v. control
(13) 1 glass fortified milk v. water
(14) Zn+Ca+VD vs. Ca+VD
(15) 2 glasses fortified milk v. water
(16) Non-anemic
(17) Anemic
