# Supplementary material for: Effects of Foods Fortified with Zinc, Alone or Cofortified with Multiple Micronutrients, on Health and Functional Outcomes: A Systematic Review and Meta-Analysis
Source: Adv Nutr. 2021 Jun 24;12(5):1821–37. doi: 10.1093/advances/nmab065 (PMC8483949; doi:10.1093/advances/nmab065)
Supplement: nmab065_Supplemental_Files [file nmab065_supplemental_files.zip › Supplemental figure 29.pdf]

Tsang BL, et al. “Effects of foods fortified with zinc, alone or co-fortified with multiple micronutrients, on health and functional outcomes: a systematic review and meta-analysis”
Online Supplementary Material
Supplemental figure 29.FAZ

	Fortified food			Control				Mean Difference
**Study or Subgroup**	**Mean**	**SD**	**Total**	**Mean**	**SD**	**Total**	**Weight**	**IV, Random, 95% CI**
Sandström 1980 (1)	0.132	0.0502	10	0.382	0.0714	10	13.4%	−0.25 [−0.30, −0.20]
López de Romaña 2005 (2)	0.133	0.041	15	0.341	0.111	7	12.1%	−0.21 [−0.29, −0.12]
Hansen 2001	0.247	0.0891	15	0.397	0.1493	15	11.9%	−0.15 [−0.24, −0.06]
López de Romaña 2005 (3)	0.237	0.052	12	0.341	0.111	7	11.9%	−0.10 [−0.19, −0.02]
Sandström 1980 (4)	0.082	0.0181	10	0.166	0.0456	10	14.2%	−0.08 [−0.11, −0.05]
Rosado 2012 (5)	0.36	0.16	10	0.35	0.22	5	6.2%	0.01 [−0.21, 0.23]
Ruz 2005	0.3	0.09	7	0.29	0.09	7	11.6%	0.01 [−0.08, 0.10]
Rosado 2012 (6)	0.37	0.22	10	0.35	0.22	5	5.6%	0.02 [−0.22, 0.26]
Méndez 2015	0.205	0.1131	14	0.17	0.02	14	13.2%	0.03 [−0.03, 0.10]
**Total (95% CI)**			**103**			**80**	**100.0%**	**−0.09 [−0.16, −0.02]**

Heterogeneity: $Tau^2 = 0.01$; $Chi^2 = 64.62$, df = 8 ($P < 0.00001$); $I^2 = 88\%$
Test for overall effect: Z = 2.56 (P = 0.01)

Mean Difference
IV, Random, 95% CI

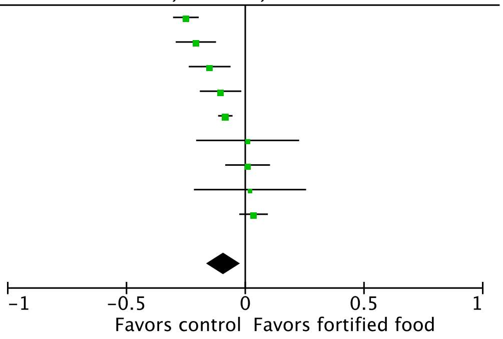

Footnotes
(1) Meal 1 vs. Meal 2 (white bread)
(2) ZN9 v ZN0
(3) ZN3 v ZN0
(4) Meal 4 vs. Meal 5 (wholemeal bread)
(5) Zinc oxide
(6) Zinc sulfate
