# Supplementary material for: Effects of Foods Fortified with Zinc, Alone or Cofortified with Multiple Micronutrients, on Health and Functional Outcomes: A Systematic Review and Meta-Analysis
Source: Adv Nutr. 2021 Jun 24;12(5):1821–37. doi: 10.1093/advances/nmab065 (PMC8483949; doi:10.1093/advances/nmab065)
Supplement: nmab065_Supplemental_Files [file nmab065_supplemental_files.zip › Supplemental figure 3.pdf]

Supplemental figure 3.effectiveness zinc deficiency prevalence duration subgroup

Study or Subgroup	log[Odds Ratio]	SE	Weight	Odds Ratio IV, Random, 95% CI
**5.4.1 <12 months**				
Tukvadze 2013	−2.9232	0.38	5.8%	0.05 [0.03, 0.11]
Kuong 2019 (1)	−1.8326	0.112	7.5%	0.16 [0.13, 0.20]
Kuong 2019 (2)	−1.3863	0.128	7.4%	0.25 [0.19, 0.32]
Kuong 2019 (3)	−1.3863	0.125	7.4%	0.25 [0.20, 0.32]
Sun 2014	−1.2575	0.123	7.4%	0.28 [0.22, 0.36]
Stuetz 2012	−1.0076	0.214	7.0%	0.37 [0.24, 0.56]
Ohiokpehai 2009	0.6066	0.26	6.6%	1.83 [1.10, 3.05]
**Subtotal (95% CI)**			**49.1%**	**0.28 [0.17, 0.44]**
Heterogeneity: $Tau^2 = 0.35; Chi^2 = 95.22, df = 6 (P < 0.00001); I^2 = 94\%$				
Test for overall effect: Z = 5.44 (P < 0.00001)				
**5.4.2 12+months**				
Engle-Stone 2017 (4)	−0.8548	0.126	7.4%	0.43 [0.33, 0.54]
Engle-Stone 2017 (5)	−0.8005	0.126	7.4%	0.45 [0.35, 0.57]
Dutta 2019	−0.5716	0.096	7.5%	0.56 [0.47, 0.68]
Varea 2011 (6)	−0.4157	0.19	7.1%	0.66 [0.45, 0.96]
Ara 2019	−0.3045	0.105	7.5%	0.74 [0.60, 0.91]
Varea 2012	0.1811	0.221	6.9%	1.20 [0.78, 1.85]
Varea 2011 (7)	0.4818	0.21	7.0%	1.62 [1.07, 2.44]
**Subtotal (95% CI)**			**50.9%**	**0.70 [0.52, 0.94]**
Heterogeneity: $Tau^2 = 0.13; Chi^2 = 49.06, df = 6 (P < 0.00001); I^2 = 88\%$				
Test for overall effect: Z = 2.39 (P = 0.02)				
**Total (95% CI)**			**100.0%**	**0.45 [0.31, 0.64]**
Heterogeneity: $Tau^2 = 0.43; Chi^2 = 287.64, df = 13 (P < 0.00001); I^2 = 95\%$				
Test for overall effect: Z = 4.44 (P < 0.00001)				
Test for subgroup differences: $Chi^2 = 10.96, df = 1 (P = 0.0009), I^2 = 90.9\%$				

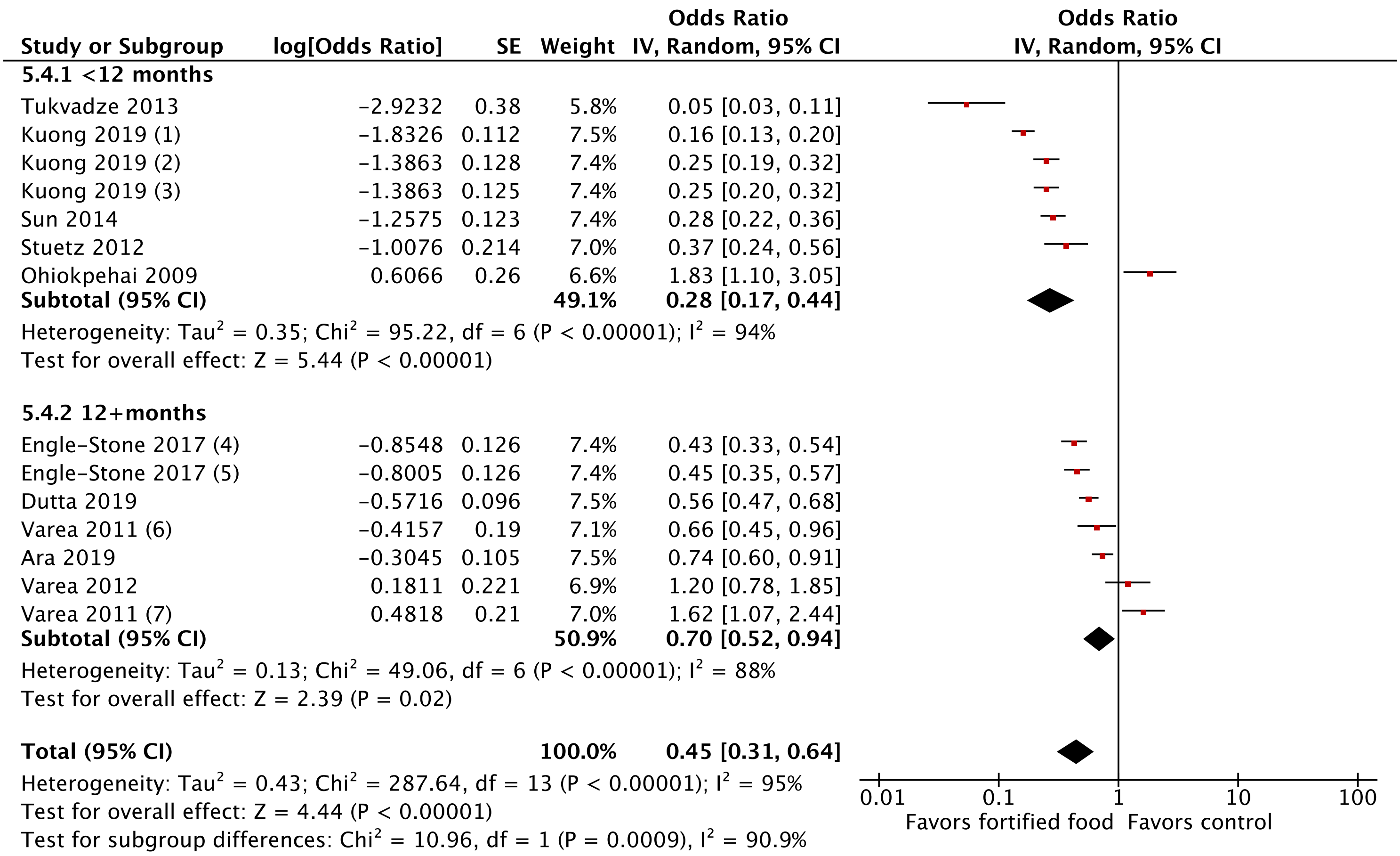

Footnotes
(1) NutriRice v. Control
(2) URN v. Control
(3) URO v. Control
(4) Women 15–49 years
(5) Children 12–59 mos
(6) Children 2–6
(7) Children 1–2
