# Supplementary material for: Effects of Foods Fortified with Zinc, Alone or Cofortified with Multiple Micronutrients, on Health and Functional Outcomes: A Systematic Review and Meta-Analysis
Source: Adv Nutr. 2021 Jun 24;12(5):1821–37. doi: 10.1093/advances/nmab065 (PMC8483949; doi:10.1093/advances/nmab065)
Supplement: nmab065_Supplemental_Files [file nmab065_supplemental_files.zip › Supplemental figure 30.pdf]

Tsang BL, et al. “Effects of foods fortified with zinc, alone or co-fortified with multiple micronutrients, on health and functional outcomes: a systematic review and meta-analysis”
Online Supplementary Material

Supplemental figure 30.TAZ

	Fortified food			Control				Mean Difference
**Study or Subgroup**	**Mean [mg/d]**	**SD [mg/d]**	**Total**	**Mean [mg/d]**	**SD [mg/d]**	**Total**	**Weight**	**IV, Random, 95% CI**
Sandström 1980 (1)	0.29	0.0647	10	0.22	0.0582	10	13.6%	0.07 [0.02, 0.12]
Ruz 2005	0.57	0.16	7	0.43	0.43	7	12.8%	0.14 [−0.20, 0.48]
Hansen 2001	0.73	0.2547	15	0.48	0.1791	15	13.4%	0.25 [0.09, 0.41]
Sandström 1980 (2)	0.48	0.181	10	0.15	0.0291	10	13.5%	0.33 [0.22, 0.44]
López de Romaña 2005 (3)	1.11	0.21	12	0.71	0.18	7	13.4%	0.40 [0.22, 0.58]
López de Romaña 2005 (4)	1.34	0.47	15	0.71	0.18	7	13.1%	0.63 [0.36, 0.90]
Méndez 2015	3.09	0.27	14	1.06	0.14	14	13.4%	2.03 [1.87, 2.19]
Rosado 2012 (5)	4.8	2.53	10	2.3	1.58	5	3.7%	2.50 [0.41, 4.59]
Rosado 2012 (6)	5	3.16	10	2.3	1.58	5	3.0%	2.70 [0.30, 5.10]
**Total (95% CI)**			**103**			**80**	**100.0%**	**0.69 [0.22, 1.16]**

Heterogeneity: $Tau^2 = 0.43$; $Chi^2 = 539.18$, df = 8 ($P < 0.00001$); $I^2 = 99\%$
Test for overall effect: Z = 2.85 (P = 0.004)

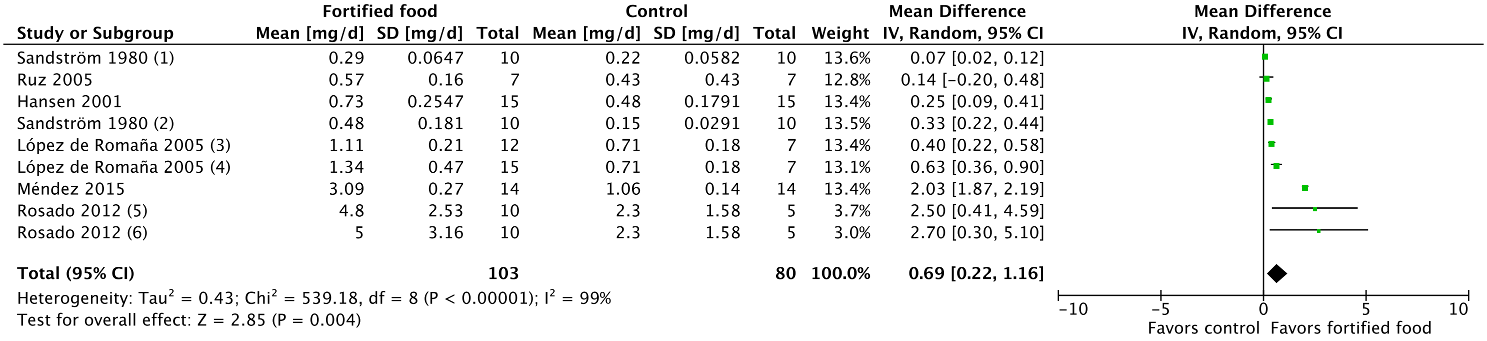

Footnotes
(1) Meal 4 vs. Meal 5 (wholemeal bread)
(2) Meal 1 vs. Meal 2 (white bread)
(3) ZN3 v. ZN0
(4) ZN9 v ZN0
(5) Zinc oxide
(6) Zinc sulfate
