# Supplementary material for: Effects of Foods Fortified with Zinc, Alone or Cofortified with Multiple Micronutrients, on Health and Functional Outcomes: A Systematic Review and Meta-Analysis
Source: Adv Nutr. 2021 Jun 24;12(5):1821–37. doi: 10.1093/advances/nmab065 (PMC8483949; doi:10.1093/advances/nmab065)
Supplement: nmab065_Supplemental_Files [file nmab065_supplemental_files.zip › Supplemental figure 32.pdf]

Tsang BL, et al. “Effects of foods fortified with zinc, alone or co-fortified with multiple micronutrients, on health and functional outcomes: a systematic review and meta-analysis”
Online Supplementary Material

Supplemental figure 32.FAZ daily dose subgroup

	Fortified food			Control				Mean Difference
**Study or Subgroup**	**Mean**	**SD**	**Total**	**Mean**	**SD**	**Total**	**Weight**	**IV, Random, 95% CI**
**14.3.1 <50%**								
Méndez 2015	0.215	0.1131	14	0.17	0.02	14	11.6%	0.04 [−0.02, 0.11]
Rosado 2012 (1)	0.37	0.07	10	0.35	0.07	5	11.0%	0.02 [−0.06, 0.10]
Rosado 2012 (2)	0.36	0.05	10	0.35	0.07	5	11.3%	0.01 [−0.06, 0.08]
Ruz 2005	0.3	0.09	7	0.29	0.09	7	10.2%	0.01 [−0.08, 0.10]
Sandström 1980 (3)	0.132	0.0502	10	0.382	0.0714	10	11.8%	−0.25 [−0.30, −0.20]
Sandström 1980 (4)	0.082	0.0181	10	0.166	0.0456	10	12.4%	−0.08 [−0.11, −0.05]
**Subtotal (95% CI)**			**61**			**51**	**68.3%**	**−0.04 [−0.13, 0.04]**
Heterogeneity: $Tau^2 = 0.01$; $Chi^2 = 70.84$, df = 5 (P < 0.00001); $I^2 = 93\%$								
Test for overall effect: Z = 0.97 (P = 0.33)								
**14.3.2 50+%**								
Hansen 2001	0.247	0.0891	15	0.397	0.1493	15	10.5%	−0.15 [−0.24, −0.06]
López de Romaña 2005 (5)	0.133	0.041	15	0.341	0.111	7	10.6%	−0.21 [−0.29, −0.12]
López de Romaña 2005 (6)	0.237	0.052	12	0.341	0.111	7	10.5%	−0.10 [−0.19, −0.02]
**Subtotal (95% CI)**			**42**			**29**	**31.7%**	**−0.15 [−0.21, −0.10]**
Heterogeneity: $Tau^2 = 0.00$; $Chi^2 = 2.82$, df = 2 (P = 0.24); $I^2 = 29\%$								
Test for overall effect: Z = 5.10 (P < 0.00001)								
**Total (95% CI)**			**103**			**80**	**100.0%**	**−0.08 [−0.15, −0.01]**
Heterogeneity: $Tau^2 = 0.01$; $Chi^2 = 82.77$, df = 8 (P < 0.00001); $I^2 = 90\%$								
Test for overall effect: Z = 2.26 (P = 0.02)								
Test for subgroup differences: $Chi^2 = 4.14$, df = 1 (P = 0.04), $I^2 = 75.9\%$								

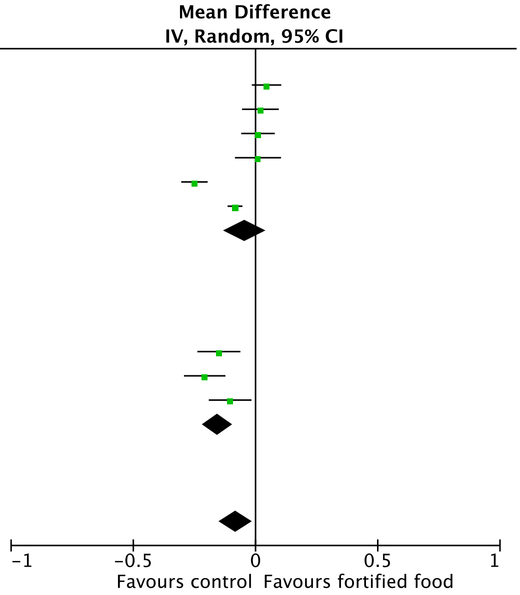

Footnotes
(1) Zinc sulfate
(2) Zinc oxide
(3) Meal 1 vs. Meal 2 (white bread)
(4) Meal 4 vs. Meal 5 (wholemeal bread)
(5) ZN9 v ZN0
(6) ZN3 v ZN0
