# Supplementary material for: Effects of Foods Fortified with Zinc, Alone or Cofortified with Multiple Micronutrients, on Health and Functional Outcomes: A Systematic Review and Meta-Analysis
Source: Adv Nutr. 2021 Jun 24;12(5):1821–37. doi: 10.1093/advances/nmab065 (PMC8483949; doi:10.1093/advances/nmab065)
Supplement: nmab065_Supplemental_Files [file nmab065_supplemental_files.zip › Supplemental figure 33.pdf]

Supplemental figure 33.TAZ daily dose subgroup

	Fortified food			Control				Mean Difference
**Study or Subgroup**	**Mean**	**SD**	**Total**	**Mean**	**SD**	**Total**	**Weight**	**IV, Random, 95% CI**
**15.3.1 <50%**								
Hansen 2001	0.73	0.2547	15	0.48	0.1791	15	11.7%	0.25 [0.09, 0.41]
Ruz 2005	0.57	0.16	7	0.43	0.43	7	11.2%	0.14 [−0.20, 0.48]
Sandström 1980 (1)	0.29	0.0647	10	0.22	0.0582	10	11.9%	0.07 [0.02, 0.12]
Sandström 1980 (2)	0.48	0.181	10	0.15	0.0291	10	11.8%	0.33 [0.22, 0.44]
**Subtotal (95% CI)**			**42**			**42**	**46.6%**	**0.20 [0.04, 0.36]**
Heterogeneity: $Tau^2 = 0.02$; $Chi^2 = 18.89$, df = 3 (P = 0.0003); $I^2 = 84\%$								
Test for overall effect: Z = 2.42 (P = 0.02)								
**15.3.2 50+%EAR**								
López de Romaña 2005 (3)	1.11	0.21	12	0.71	0.18	7	11.7%	0.40 [0.22, 0.58]
López de Romaña 2005 (4)	1.34	0.47	15	0.71	0.18	7	11.4%	0.63 [0.36, 0.90]
Méndez 2015	3.09	0.27	14	1.06	0.14	14	11.7%	2.03 [1.87, 2.19]
Rosado 2012 (5)	4.8	0.8	10	2.3	0.5	5	9.6%	2.50 [1.84, 3.16]
Rosado 2012 (6)	5	1	10	2.3	0.5	5	9.0%	2.70 [1.94, 3.46]
**Subtotal (95% CI)**			**61**			**38**	**53.4%**	**1.61 [0.71, 2.52]**
Heterogeneity: $Tau^2 = 1.01$; $Chi^2 = 226.62$, df = 4 (P < 0.00001); $I^2 = 98\%$								
Test for overall effect: Z = 3.49 (P = 0.0005)								
**Total (95% CI)**			**103**			**80**	**100.0%**	**0.93 [0.47, 1.40]**
Heterogeneity: $Tau^2 = 0.48$; $Chi^2 = 611.86$, df = 8 (P < 0.00001); $I^2 = 99\%$								
Test for overall effect: Z = 3.91 (P < 0.0001)								
Test for subgroup differences: $Chi^2 = 9.04$, df = 1 (P = 0.003), $I^2 = 88.9\%$								

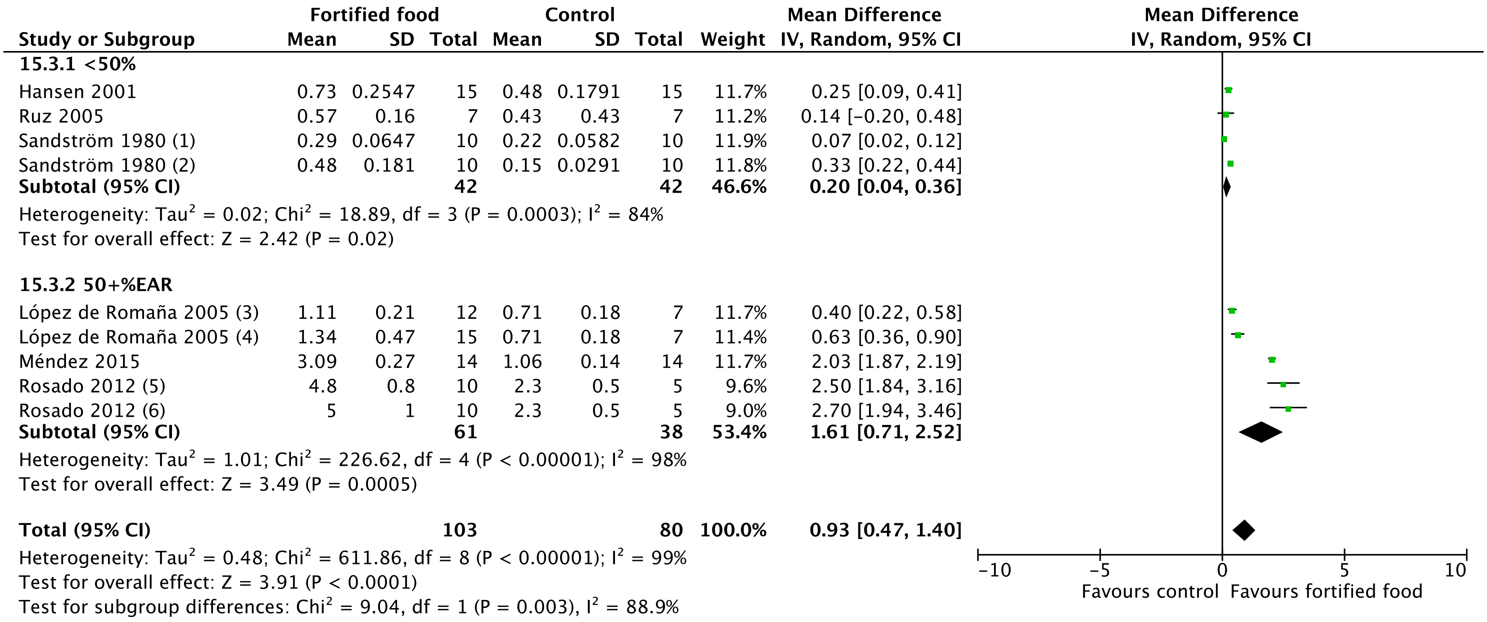

Footnotes
(1) Meal 4 vs. Meal 5 (wholemeal bread)
(2) Meal 1 vs. Meal 2 (white bread)
(3) ZN3 v. ZN0
(4) ZN9 v ZN0
(5) Zinc oxide
(6) Zinc sulfate
