# Supplementary material for: Effects of Foods Fortified with Zinc, Alone or Cofortified with Multiple Micronutrients, on Health and Functional Outcomes: A Systematic Review and Meta-Analysis
Source: Adv Nutr. 2021 Jun 24;12(5):1821–37. doi: 10.1093/advances/nmab065 (PMC8483949; doi:10.1093/advances/nmab065)
Supplement: nmab065_Supplemental_Files [file nmab065_supplemental_files.zip › Supplemental figure 5.pdf]

Supplemental figure 5.efficacy PZC food vehicles subgroup

Study or Subgroup	Fortified food Mean	Fortified food SD	Fortified food Total	Control Mean	Control SD	Control Total	Weight	Mean Difference IV, Random, 95% CI
**2.2.1 Beverages**								
Aaron 2011a (36)	6.54	20.67	250	3.92	20.42	244	3.9%	2.62 [−1.00, 6.24]
Abrams 2003 (87)	84.97	13.07	130	78.43	13.07	124	4.0%	6.54 [3.32, 9.76]
Angeles-Agdeppa 2011 (29)	116.3	26.1	46	96.6	22.7	43	2.1%	19.70 [9.55, 29.85]
Angeles-Agdeppa 2017 (86) (1)	73	22.45	56	68	14.97	19	2.4%	5.00 [−3.94, 13.94]
Angeles-Agdeppa 2017 (86) (2)	66	14.967	56	68	14.97	20	2.7%	−2.00 [−9.64, 5.64]
Angeles-Agdeppa 2017 (86) (3)	67	22.847	58	68	14.97	19	2.4%	−1.00 [−9.94, 7.94]
Bardosono 2009 (82)	−19.67	21.18	121	−20.39	24.31	124	3.3%	0.72 [−4.99, 6.43]
Costarelli 2014 (35)	101.24	40.73	21	89.28	33.85	21	0.7%	11.96 [−10.69, 34.61]
Do 2009 (69)	82.35	16.99	150	79.74	13.07	151	3.9%	2.61 [−0.82, 6.04]
Hyder 2007 (81)	88.65	31.98	433	85.4	29.74	470	3.8%	3.25 [−0.79, 7.29]
Méndez 2012 (65)	116	26.9	39	98.5	26.6	47	1.9%	17.50 [6.14, 28.86]
Sazawal 2010 (74)	61.4	26.8	233	63.4	29.4	232	3.5%	−2.00 [−7.11, 3.11]
Sazawal 2013 (78)	9.44	28.56	278	8.1	26.21	293	3.6%	1.34 [−3.16, 5.84]
Thankachan 2013 (67)	130.2	28.5	115	129.4	26.8	120	2.9%	0.80 [−6.28, 7.88]
Trinidad 2015 (27) (4)	109	26.57	42	85.6	35.42	20	1.1%	23.40 [5.92, 40.88]
Trinidad 2015 (27) (5)	121.5	32.4	42	85.6	35.42	20	1.0%	35.90 [17.54, 54.26]
Villalpando 2006 (75)	82.42	6.27	58	85.42	6.54	57	4.1%	−3.00 [−5.34, −0.66]
Wibowo 2016 (37)	51	8.14	46	48.8	8.6	58	4.0%	2.20 [−1.03, 5.43]
**Subtotal (95% CI)**			**2174**			**2082**	**51.1%**	**3.73 [1.13, 6.33]**
Heterogeneity: $Tau^2 = 18.98$; $Chi^2 = 68.82$, df = 17 (P < 0.00001); $I^2 = 75\%$								
Test for overall effect: Z = 2.81 (P = 0.005)								
**2.2.2 Cereal grains**								
Aaron 2011b (90) (6)	1.96	8.64	33	3.07	8.64	16	3.4%	−1.11 [−6.27, 4.05]
Aaron 2011b (90) (7)	2.09	8.73	31	3.07	8.64	17	3.4%	−0.98 [−6.11, 4.15]
Badii 2011 (91) (8)	78.73	8.1	25	67.73	2.17	12	3.9%	11.00 [7.60, 14.40]
Badii 2011 (91) (9)	86.8	10.78	25	67.73	2.17	13	3.7%	19.07 [14.68, 23.46]
Haibin 2001 (59) (10)	109.8	84.97	28	74.51	76.47	29	0.2%	35.29 [−6.72, 77.30]
Haibin 2001 (59) (11)	100	55.56	10	82.35	41.83	9	0.2%	17.65 [−26.31, 61.61]
Hettiarachchi 2004 (92) (12)	84.97	16.34	13	80.39	18.95	12	1.5%	4.58 [−9.34, 18.50]
Hettiarachchi 2004 (92) (13)	83.01	16.99	12	79.08	14.38	12	1.7%	3.93 [−8.66, 16.52]
Hieu 2012 (70)	71.24	16.99	101	70.59	16.99	95	3.6%	0.65 [−4.11, 5.41]
Ibnu 2019 (28) (14)	66.59	6.76	32	59.06	7.81	32	3.9%	7.53 [3.95, 11.11]
Ibnu 2019 (28) (15)	53.73	8.41	30	57.52	9.43	30	3.6%	−3.79 [−8.31, 0.73]
Kiliç 1998 (41)	81.57	9.08	12	62.94	3.4	12	3.3%	18.63 [13.14, 24.12]
López de Romaña 2005 (77) (16)	82.4	9.9	12	77.6	12.3	6	1.9%	4.80 [−6.52, 16.12]
López de Romaña 2005 (77) (17)	76.2	8.2	10	77.6	12.3	6	1.9%	−1.40 [−12.48, 9.68]
Nga 2009 (24)	61.44	12.42	232	57.19	12.42	235	4.2%	4.25 [2.00, 6.50]
Pinkaew 2013 (71)	72.55	8.5	90	69.28	9.15	82	4.1%	3.27 [0.62, 5.92]
**Subtotal (95% CI)**			**696**			**618**	**44.5%**	**5.58 [2.00, 9.16]**
Heterogeneity: $Tau^2 = 36.53$; $Chi^2 = 110.38$, df = 15 (P < 0.00001); $I^2 = 86\%$								
Test for overall effect: Z = 3.06 (P = 0.002)								
**2.2.3 Condiments**								
Vinodkumar 2009 (76)	142.5	132	45	102.73	88.78	50	0.2%	39.77 [−5.98, 85.52]
Winichagoon 2006 (80)	73.2	10.65	247	71.24	10.92	261	4.2%	1.96 [0.08, 3.84]
**Subtotal (95% CI)**			**292**			**311**	**4.4%**	**13.67 [−20.59, 47.94]**
Heterogeneity: $Tau^2 = 441.92$; $Chi^2 = 2.62$, df = 1 (P = 0.11); $I^2 = 62\%$								
Test for overall effect: Z = 0.78 (P = 0.43)								
**Total (95% CI)**			**3162**			**3011**	**100.0%**	**4.68 [2.62, 6.75]**
Heterogeneity: $Tau^2 = 25.25$; $Chi^2 = 204.42$, df = 35 (P < 0.00001); $I^2 = 83\%$								
Test for overall effect: Z = 4.45 (P < 0.00001)								
Test for subgroup differences: $Chi^2 = 0.96$, df = 2 (P = 0.62), $I^2 = 0\%$								

Mean Difference
IV, Random, 95% CI

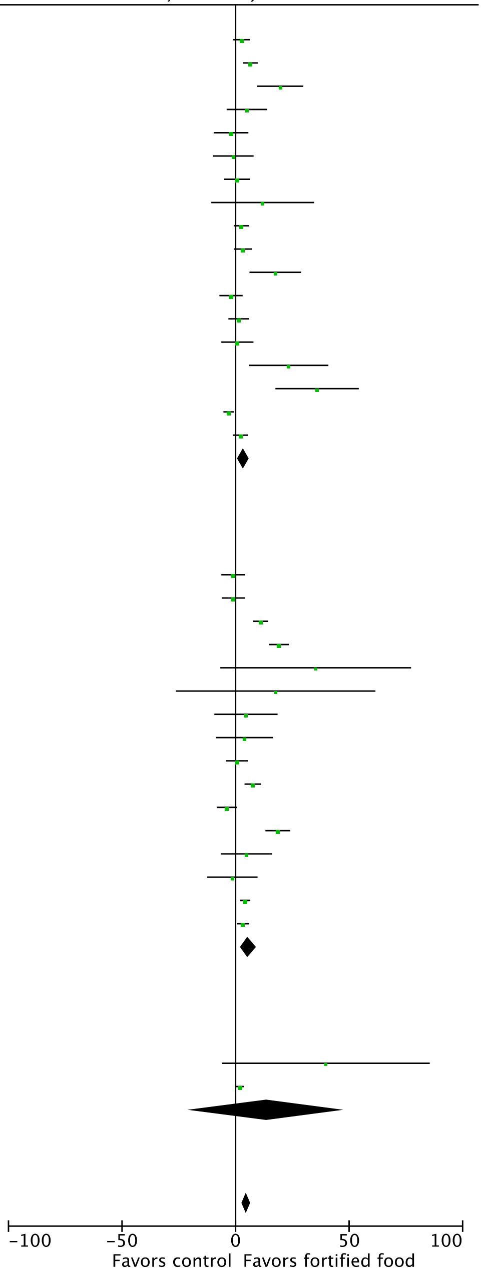

Footnotes
(1) Moderate (5 drinks/wk, ~4 mg/d on average)
(2) Low (3 beverages/wk, ~2.4 mg/d on average)
(3) High (7 drinks/wk, 5.6 mg/d)
(4) 1 glass fortified milk v. water
(5) 2 glasses fortified milk v. water
(6) 7.5 mg/d
(7) 15 mg/d
(8) 100 mg/kg v. Control
(9) 50 mg/kg v. control
(10) Zn+Ca+VD vs. Ca+VD
(11) Zn+Ca+VD+Fe vs. Ca+VD+Fe
(12) FeSO4+FA
(13) Na2EDTA+FeSO4+FA
(14) Anemic
(15) Non-anemic
(16) ZN9 v. ZN0
(17) ZN3 v. ZN0
