# Supplementary material for: Effects of Foods Fortified with Zinc, Alone or Cofortified with Multiple Micronutrients, on Health and Functional Outcomes: A Systematic Review and Meta-Analysis
Source: Adv Nutr. 2021 Jun 24;12(5):1821–37. doi: 10.1093/advances/nmab065 (PMC8483949; doi:10.1093/advances/nmab065)
Supplement: nmab065_Supplemental_Files [file nmab065_supplemental_files.zip › Supplemental figure 6.pdf]

Tsang BL, et al. “Effects of foods fortified with zinc, alone or co-fortified with multiple micronutrients, on health and functional outcomes: a systematic review and meta-analysis”
Online Supplementary Material

Supplemental figure 6.efficacy zinc deficiency prevalence food vehicle subgroup

Study or Subgroup	log[Odds Ratio]	SE	Weight	Odds Ratio IV, Random, 95% CI
**4.2.1 Cereal grains**				
López de Romaña 2005 (1)	−0.9808	1.112	1.1%	0.38 [0.04, 3.32]
Nga 2009	−0.6203	0.132	9.7%	0.54 [0.42, 0.70]
López de Romaña 2005 (2)	−0.5754	1.132	1.0%	0.56 [0.06, 5.17]
Pinkaew 2013	−0.481	0.221	8.0%	0.62 [0.40, 0.95]
Aaron 2011b (3)	0.8026	0.359	5.5%	2.23 [1.10, 4.51]
Aaron 2011b (4)	1.2001	0.36	5.5%	3.32 [1.64, 6.72]
**Subtotal (95% CI)**			**30.8%**	**1.03 [0.51, 2.08]**
Heterogeneity: $Tau^2 = 0.53$; $Chi^2 = 33.71$, df = 5 (P < 0.00001); $I^2 = 85\%$				
Test for overall effect: Z = 0.07 (P = 0.94)				
**4.2.2 Beverages**				
Angeles-Agdeppa 2011	−1.2164	0.553	3.3%	0.30 [0.10, 0.88]
Abrams 2003	−1.1994	0.249	7.5%	0.30 [0.18, 0.49]
Aaron 2011a	−0.4463	0.129	9.7%	0.64 [0.50, 0.82]
Angeles-Agdeppa 2017 (5)	−0.3663	0.266	7.1%	0.69 [0.41, 1.17]
Bardosono 2009	−0.2672	0.183	8.7%	0.77 [0.53, 1.10]
Angeles-Agdeppa 2017 (6)	−0.1386	0.263	7.2%	0.87 [0.52, 1.46]
Do 2009	−0.1151	0.185	8.7%	0.89 [0.62, 1.28]
Angeles-Agdeppa 2017 (7)	0.06899	0.265	7.2%	1.07 [0.64, 1.80]
**Subtotal (95% CI)**			**59.4%**	**0.68 [0.52, 0.88]**
Heterogeneity: $Tau^2 = 0.09$; $Chi^2 = 19.52$, df = 7 (P = 0.007); $I^2 = 64\%$				
Test for overall effect: Z = 2.91 (P = 0.004)				
**4.2.3 Condiments**				
Winichagoon 2006	−0.3348	0.126	9.8%	0.72 [0.56, 0.92]
**Subtotal (95% CI)**			**9.8%**	**0.72 [0.56, 0.92]**
Heterogeneity: Not applicable				
Test for overall effect: Z = 2.66 (P = 0.008)				
**Total (95% CI)**			**100.0%**	**0.76 [0.60, 0.96]**
Heterogeneity: $Tau^2 = 0.13$; $Chi^2 = 53.38$, df = 14 (P < 0.00001); $I^2 = 74\%$				
Test for overall effect: Z = 2.28 (P = 0.02)				
Test for subgroup differences: $Chi^2 = 1.19$, df = 2 (P = 0.55), $I^2 = 0\%$				

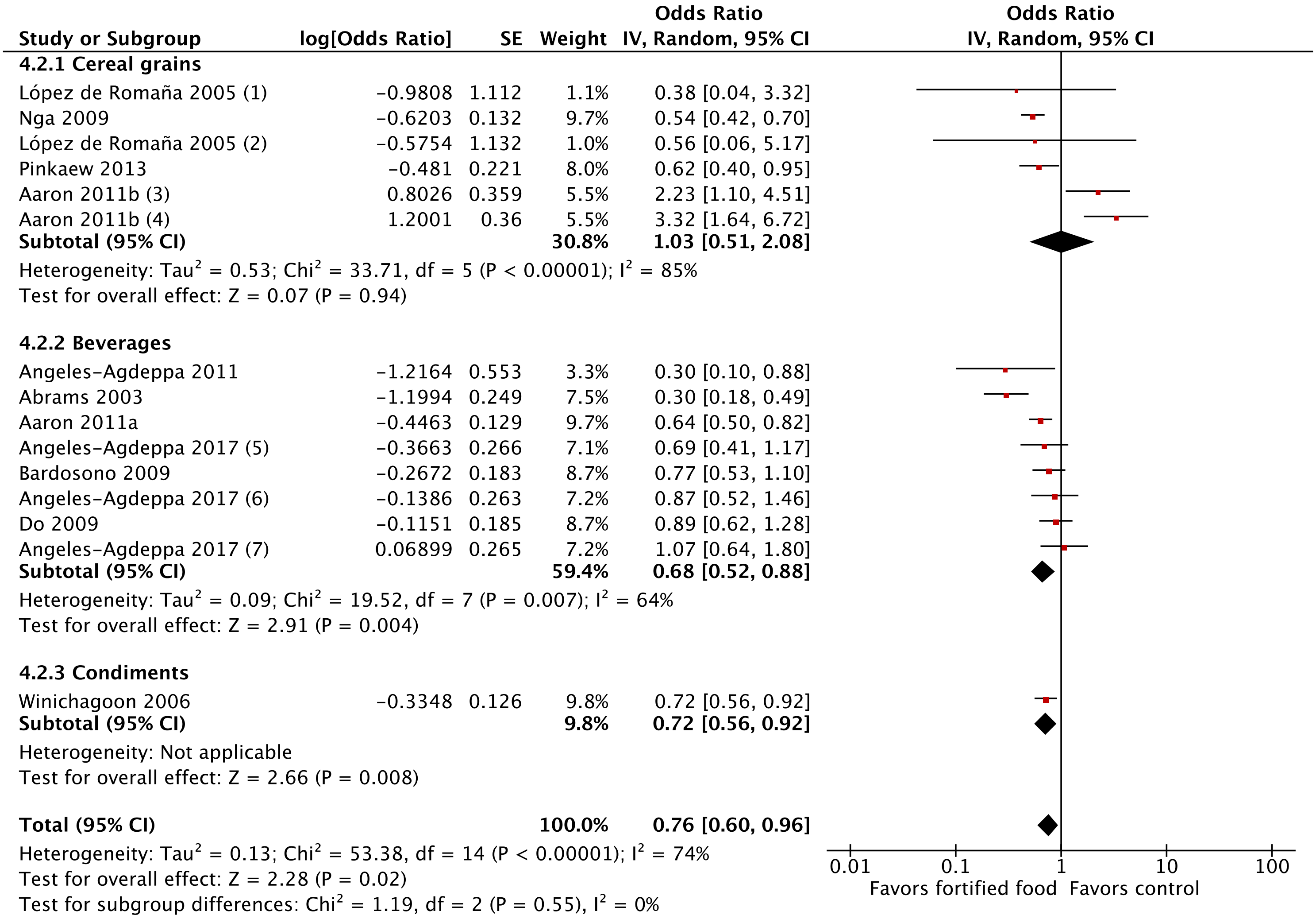

Footnotes
(1) ZN0 vs ZN9
(2) ZN0 vs ZN3
(3) 15 mg/d
(4) 7.5 mg/d
(5) Moderate (5 drinks/wk, ~4 mg/d on average)
(6) High (7 drinks/wk, 5.6 mg/d)
(7) Low (3 beverages/wk, ~2.4 mg/d on average)
