# Supplementary material for: Effects of Foods Fortified with Zinc, Alone or Cofortified with Multiple Micronutrients, on Health and Functional Outcomes: A Systematic Review and Meta-Analysis
Source: Adv Nutr. 2021 Jun 24;12(5):1821–37. doi: 10.1093/advances/nmab065 (PMC8483949; doi:10.1093/advances/nmab065)
Supplement: nmab065_Supplemental_Files [file nmab065_supplemental_files.zip › Supplemental Table 1. Overview of included studies.docx]

**Table S1: Overview of 59 studies (72 records) assessing foods fortified with zinc (alone or co-fortified with multiple micronutrients) on health and functional outcomes^[[1]](#endnote-1),^^[[2]](#endnote-2)^**

| Reference | Location, *Region^[[3]](#endnote-3)^* | Fortified Food^[[4]](#endnote-4)^ | Compound | Participant Age^[[5]](#endnote-5)^ | Adherence^[[6]](#endnote-6)^ | Outcome(s) Measured |
| --- | --- | --- | --- | --- | --- | --- |
| Aaron et al. 2011a (1) | Nigeria  *WCA* | Beverage | Zinc oxide and zinc glycinate Chelate | 5-13 y | High | PZC  Zinc deficiency prevalence  Morbidity |
| Abrams et al. 2003 (2) | Botswana  *ESA* | Beverage | Zinc gluconate | 5-11 y | High | Zinc deficiency prevalence  Anthropometry |
| Angeles-Agdeppa et al. 2011 (3) | Philippines  *EAP* | Beverage | Zinc sulfate | 6-9 y | NR | PZC  Zinc deficiency prevalence  Morbidity  Anthropometry |
| Angeles-Agdeppa et al. 2017 (4) | Philippines  *EAP* | Beverage | NR | 6-9 y | High | PZC |
| Hyder et al. 2007 (5) | Bangladesh  *SA* | Beverage | NR | C: 12 y  I: 11.9 y^[[7]](#endnote-7)^ | High | PZC |
| Mishaan et al. 2004 (6) | Peru  *LAC* | Beverage | Zinc gluconate | 6-9 y | High | FAZ/TAZ |
| Rameshwar Sarma et al. 2006 (7)  Sivakumar et al. 2006 (8)  Vazir et al. 2006 (9) | India  *SA* | Beverage | NR | 6-18 y | High | Morbidity  Anthropometry  Cognition  Immune function |
| Thankachan et al. 2013 (10) | India  *SA* | Beverage | Zinc sulfate | 6-12 y | High | PZC |
| Tukvadze & Kverenchkhiladze 2013 (11) | Georgia  *EECA* | Tea | NR | 11-14 y | Low | Zinc deficiency prevalence  Hair zinc  Urine zinc |
| Bardosono et al. 2009 (12) | Indonesia  *EAP* | Milk, powder | NR | 7-9 y | High | PZC  Zinc deficiency prevalence  Anthropometry  Cognition |
| Dhingra et al. 2004 (13)  Marwah et al. 2004 (14)  Sazawal et al. 2007 (15)  Sazawal et al. 2010 (16) | India  *SA* | Milk, powder | NR | 1-3 y | High | Morbidity  Anthropometry |
| Méndez et al. 2012 (17) | Mexico  *LAC* | Milk, powder | NR | 12-17 y | High | PZC |
| Méndez et al. 2014 (18)  Méndez et al. 2015 (19) | Mexico  *LAC* | Milk, powder | Zinc oxide | 12-16 y | High | PZC  FAZ/TAZ |
| Ruz et al. 2005 (20) | Chile  *LAC* | Milk, powder | Zinc acetate | 41.9 y^[[8]](#endnote-8)^ | High | FAZ/TAZ |
| Trinidad et al. 2015 (21) | Philippines  *EAP* | Milk, powder | NR | ≥ 6 y | NR | PZC  Anthropometry |
| Villalpando et al. 2006 (22) | Mexico  *LAC* | Milk, powder | Zinc oxide | 10-30 mo | High | PZC |
| Wibowo et al. 2016 (23) | Indonesia  *EAP* | Milk, powder | NR | 18-35 y | High | PZC  Plasma fatty acids |
| Do et al. 2009 (24) | Viet Nam  *EAP* | Milk, liquid | NR | 7-8 y | NR | PZC  Zinc deficiency prevalence  Anthropometry  Cognition  Immune function |
| Petrova et al. 2019 (25) | Spain  *WE* | Milk, liquid | NR | 8-14 y | High | Cognition  Immune function  Plasma fatty acids |
| Costarelli et al. 2014 (26) | Italy  *WE* | Milk, skim | Zinc gluconate | ≥ 82 y | NR | PZC  Immune function  Copper interaction |
| Sazawal et al. 2013 (27) | India  *SA* | Yogurt | NR | 6-9 y | High | PZC  Anthropometry |
| Muthayya et al. 2009 (28)  Thomas et al. 2012 (29) | India  *SA* | Wheat flour, biscuit & milk, powder | NR | 6-10 y | High | Anthropometry  Immune function  Plasma fatty acids  Morbidity |
| Engle-Stone et al. 2017 (30) | Cameroon  *WCA* | Wheat flour | Zinc oxide | 12-59 mo,  15-49 y | NR | PZC  Zinc deficiency prevalence  Morbidity  Anthropometry  Change in FF intake |
| Huo et al. 2011 (31) | China  *EAP* | Wheat flour | Zinc oxide | 20-60 y | High | PZC  Change in FF intake |
| Huo et al. 2012 (32) | China  *EAP* | Wheat flour | Zinc oxide | 20-60 y | NR | PZC  Change in FF intake |
| Jiang et al. 2011 (33) | China  *EAP* | Wheat flour | NR | WRA | NA | Plasma fatty acids |
| Stuetz et al. 2012 (34) | Thailand, (Maela refugee camp)  *EAP* | Wheat flour | NR | 16-46 y | NR | PZC  Zinc deficiency prevalence  Copper interaction |
| Hieu et al. 2012 (35) | Viet Nam  *EAP* | Wheat flour, biscuits | Zinc sulfate | 6-9 y | High | PZC |
| Nga et al. 2009 (36)  Nga et al. 2011 (37) | Viet Nam  *EAP* | Wheat flour, biscuits | Zinc sulfate | 6-8 y | High | PZC  Zinc deficiency prevalence  Cognition  Immune function |
| López de Romaña et al. 2003 (38) | Peru  *LAC* | Wheat flour, biscuits | Zinc sulfate, zinc oxide | 3-4 y | High | FAZ/TAZ |
| Sari et al. 2014 (39) | Indonesia  *EAP* | Wheat flour, biscuits | NR | 4-5 y | High | Immune function |
| López de Romaña et al. 2005 (40) | Peru  *LAC* | Wheat flour, biscuits and noodles | Zinc sulfate | 3-4 y | Low | PZC  Zinc deficiency prevalence  Anthropometry  FAZ/TAZ  Iron interaction |
| Aaron et al. 2011b (41) | Senegal  *WCA* | Wheat flour, bread | Zinc oxide | 18+ y | High | PZC  Zinc deficiency prevalence |
| Badii et al. 2011 (42) | Iran  *MENA* | Wheat flour, bread (Iranian) | Zinc sulfate | 32.2 y^[[9]](#endnote-9)^ | High | PZC  Iron interaction |
| Hansen et al. 2001 (43) | Denmark  *WE* | Wheat flour, bread | Zinc chloride | 22-33 y | High | FAZ/TAZ |
| Sandström et al. 1980 (44) | Sweden  *WE* | Wheat flour, bread | Zinc chloride | 19-61 y | NR | FAZ/TAZ |
| Saldamli et al. 1996 (45)  Kiliç et al. 1998 (46) | Turkey  *EECA* | Wheat flour, bread | Zinc acetate | 7-11 y | NR | Morbidity  Anthropometry  PZC  Copper interaction  Iron interaction |
| Herman et al. 2002 (47) | Indonesia  *EAP* | Wheat flour, dumplings | Zinc sulfate | 4-8 y | High | FAZ/TAZ |
| Hambidge et al. 1979 (48) | United States  *NA* | Breakfast cereal | Zinc oxide | 33-90 mo | High | PZC  Copper Interaction  Hair zinc  Urine zinc |
| Nieman et al. 2011 (49) | United States  *NA* | Breakfast cereal | NR | 7-13 y | High | Morbidity  Anthropometry |
| Ara et al. 2019 (50) | Bangladesh  *SA* | Rice | NR | 15-49 y | NR | PZC  Zinc deficiency prevalence  Morbidity  Change in FF intake |
| De Gier et al. 2016 (51)  Fiorentino et al. 2018 (52)  Kuong et al. 2019 (53) | Cambodia  *EAP* | Rice | NR | 6-16 y | High | Morbidity  Cognition  Immune function  PZC  Zinc deficiency prevalence |
| Della Lucia et al. 2016 (54)  Della Lucia et al. 2017 (55) | Brazil  *LAC* | Rice | Zinc oxide | 2-6 y | Low | PZC |
| Dutta et al. 2019 (56) | India  *SA* | Rice | NR | 6-14 y | NR | Zinc deficiency prevalence  Change in FF intake |
| Hackl et al. 2017 (57) | Switzerland  *WE* | Rice | Zinc oxide, zinc sulfate | 18-40 y | Low | FAZ/TAZ |
| Ibnu et al. 2019 (58) | Indonesia  *EAP* | Rice | NR | 12-15 y | NR | PZC |
| Pinkaew et al. 2013 (59) | Thailand  *EAP* | Rice | NR | 4-12 y | High | PZC  Zinc deficiency prevalence |
| Sun et al. 2013 (60) | China  *EAP* | Rice | Zinc dioxide | 11-16 y | High | PZC  Zinc deficiency prevalence |
| Hettiarachchi et al. 2004 (61) | Sri Lanka  *EAP* | Rice, flour | Zinc oxide | 7-10 y | NR | PZC  FAZ/TAZ |
| Varea et al. 2011 (62) | Argentina  *LAC* | Maize flour | Zinc sulfate | 1-6 y | High | PZC  Zinc deficiency prevalence  Anthropometry |
| Varea et al. 2012 (63) | Argentina  *LAC* | Maize flour | Zinc sulfate | 15-47 y | High | PZC  Zinc deficiency prevalence |
| Hotz et al. 2005 (64) | Mexico  *LAC* | Maize flour, tortillas | Zinc oxide, zinc sulfate | 19-44 y | NR | FAZ/TAZ |
| Rosado et al. 2012 (65) | Mexico  *LAC* | Maize flour, tortillas | Zinc oxide, zinc sulfate | 21-51 y | High | FAZ/TAZ |
| Chen et al. 2008 (66)  Chen et al. 2011 (67) | China  *EAP* | Seasoning powder | Zinc oxide | 2-6 y | High | Anthropometry  Morbidity |
| Winichagoon et al. 2006 (68)  Manger et al. 2008 (69) | Thailand  *EAP* | Seasoning powder | Zinc sulfate | 5.5-13.4 y | High | PZC  Zinc deficiency prevalence  Morbidity  Anthropometry  Cognition  Immune function |
| Vinodkumar et al. 2009 (70) | India  *SA* | Salt | Zinc oxide | I: 12.21 y  C: 12.27 y^7^ | High | PZC  Morbidity  Cognition  Immune function |
| Ohiokpehai et al 2009 (71) | Kenya  *ESA* | Corn-soy porridge | NR | 6-9 y | NR | PZC  Zinc deficiency prevalence |
| Haibin et al 2001 (72) | China  *EAP* | Flour (unspecified), biscuits | Zinc lactate | 24.7^7^ ± 2.4 y | NR | PZC |

Abbreviations (in alphabetical order): C, Control Group; EAP, East Asia and Pacific; EECA, Eastern Europe and Central Asia; ESA, Eastern and Southern Africa; FAZ, Fractional Zinc Absorption; FF, fortified food; I, Intervention Group; LAC, Latin America and Caribbean; MENA, Middle East and North Africa; NA, North America; NR, Not reported; PZC, plasma/serum zinc concentrations; SA, South Asia; TAZ, Total Absorbed Zinc; WCA, West and Central Africa; WE, Western Europe; WRA, women of reproductive age

**References**

1. Aaron GJ, Kariger P, Aliyu R, Flach M, Iya D, Obadiah M, et al. A Multi-Micronutrient Beverage Enhances the Vitamin A and Zinc Status of Nigerian Primary Schoolchildren. Journal of Nutrition. 2011;141(8):1565–72.

2. Abrams SA, Mushi A, Hilmers DC, Griffin IJ, Davila P, Allen L. A multinutrient-fortified beverage enhances the nutritional status of children in Botswana. Journal of Nutrition. 2003;133(6):1834–40.

3. Angeles-Agdeppa I, Magsadia CR, Capanzana MV. Fortified juice drink improved iron and zinc status of schoolchildren. Asia Pacific Journal of Clinical Nutrition. 2011;20(4):535–43.

4. Angeles-Agdeppa I, Magsadia CR, Aaron GJ, Lloyd BB, Hilmers DC, Bhutta ZA. A Micronutrient Fortified Beverage Given at Different Dosing Frequencies Had Limited Impact on Anemia and Micronutrient Status in Filipino Schoolchildren. Nutrients [Internet]. 2017;9(9). Available from: ://WOS:000411973200085

5. Ziauddin Hyder SM, L©œnnerdal B, Rahman M, Mehansho H, Mannar V, Khan M, et al. A Multiple-Micronutrient-Fortified Beverage Affects Hemoglobin, Iron, and Vitamin A Status and Growth in Adolescent Girls in Rural Bangladesh. Journal of Nutrition. 2007;137(9):2147–53.

6. Mishaan AMA, Zavaleta N, Griffin IJ, Hilmers DC, Hawthorne KM, Abrams SA. Bioavailability of iron and zinc from a multiple micronutrient-fortified beverage. Journal of Pediatrics. 2004;145(1):26–31.

7. Rameshwar Sarma KV, Udaykumar P, Balakrishna N, Vijayaraghavan K, Sivakumar B. Effect of micronutrient supplementation on health and nutritional status of schoolchildren: Growth and morbidity. Nutrition. 2006;22(1 SUPPL.):S8–14.

8. Sivakumar B, Nair KM, Sreeramulu D, Suryanarayana P, Ravinder P, Shatrugna V, et al. Effect of micronutrient supplement on health and nutritional status of schoolchildren: biochemical status. Nutrition. 2006 Jan;22(1 Suppl):S15-25.

9. Vazir S, Nagalla B, Thangiah V, Kamasamudram V, Bhattiprolu S. Effect of micronutrient supplement on health and nutritional status of schoolchildren: mental function. Nutrition. 2006 Jan;22(1 Suppl):S26-32.

10. Thankachan P, Selvam S, Surendran D, Chellan S, Pauline M, Abrams SA, et al. Efficacy of a multi micronutrient-fortified drink in improving iron and micronutrient status among schoolchildren with low iron stores in India: A randomised, double-masked placebo-controlled trial. European Journal of Clinical Nutrition. 2013;67(1):36–41.

11. Tukvadze S, Kverenchkhiladze R. Inclusion of zinc fortified tea into the children’s diet and its hygienicassessment. Georgian medical news. 2013;(217):53–6.

12. Bardosono S, Dewi LE, Sukmaniah S, Permadhi I, Eka AD, Lestarina L. Effect of a six-month iron-zinc fortified milk supplementation on nutritional status, physical capacity and speed learning process in Indonesian underweight schoolchildren: Randomized, placebo-controlled. Medical Journal of Indonesia. 2009;18(3):193–202.

13. Dhingra P, Menon VP, Sazawal S, Dhingra U, Marwah D, Sarkar A, et al. Effect of fortification of milk with zinc and iron along with vitamins C, E, A and selenium on growth, iron status and development in preschool children - A community based double-masked randomized trial [Internet]. 2004. 53 p. Available from: ://WOS:000227354700008

14. Marwah D, Sazawal S, Dhingra U, Verma P, Deb S, Dhingra P, et al. Efficacy of micronutrient fortification of milk in prevention of childhood morbidity in children 1-3 y of age - A community based double masked randomized trial [Internet]. 2004. 367 p. Available from: ://WOS:000227354700062

15. Sazawal S, Dhingra U, Dhingra P, Hiremath G, Kumar J, Sarkar A, et al. Effects of fortified milk on morbidity in young children in north India: community based, randomised, double masked placebo controlled trial. Bmj. 2007;334(7585):140.

16. Sazawal S, Dhingra U, Dhingra P, Hiremath G, Sarkar A, Dutta A, et al. Micronutrient fortified milk improves iron status, anemia and growth among children 1-4 y: A double masked, randomized, controlled trial. PLoS ONE [Internet]. 2010;5(8). Available from: https://www.scopus.com/inward/record.uri?eid=2-s2.0-77957861492&doi=10.1371%2fjournal.pone.0012167&partnerID=40&md5=7c3940d6762d5c82fbccb481dc8cce42

17. Mendez RO, Galdamez K, Grijalva MI, Quihui L, Garcia HS, de la Barca AM. Effect of micronutrient-fortified milk on zinc intake and plasma concentration in adolescent girls. J Am Coll Nutr. 2012;31(6):408–14.

18. Méndez RO, Santiago A, Yepiz-Plascencia G, Peregrino-Uriarte AB, Calderón de la Barca AM, García HS. Zinc fortification decreases ZIP1 gene expression of some adolescent females with appropriate plasma zinc levels. Nutrients. 2014;6(6):2229–39.

19. Mendez RO, Hambidge M, Baker M, Salgado SA, Ruiz J, Garcia HS, et al. Zinc Absorption from Fortified Milk Powder in Adolescent Girls. Biol Trace Elem Res. 2015;168(1):61–6.

20. Ruz M, Codoceo J, Inostroza J, Rebolledo A, Krebs NF, Westcott JE, et al. Zinc absorption from a micronutrient-fortified dried cow’s milk used in the Chilean National Complementary Food Program. Nutrition Research. 2005;25(12):1043–8.

21. Trinidad TP, Mallillin AC, Sagum RS, de Leon MP, Borlagdan MS, Baquiran AFP. Fortified milk consumption among 6-year old children: changes in biochemical markers of trace minerals and vitamins. Trace Elements and Electrolytes. 2015;32(3):112–8.

22. Villalpando S, Shamah T, Rivera JA, Lara Y, Monterrubio E. Fortifying milk with ferrous gluconate and zinc oxide in a public nutrition program reduced the prevalence of anemia in toddlers. Journal of Nutrition. 2006;136(10):2633–7.

23. Wibowo N, Bardosono S, Irwinda R. Effects of Bifidobacterium animalis lactis HN019 (DR10TM), inulin, and micronutrient fortified milk on faecal DR10TM, immune markers, and maternal micronutrients among Indonesian pregnant women. Asia Pacific Journal of Clinical Nutrition. 2016;25:S102–10.

24. Do TKL, Bui TN, Nguyen CK, Le TH, Nguyen TQN, Nguyen TH, et al. Impact of milk consumption on performance and health of primary school children in rural Vietnam. Asia Pacific Journal of Clinical Nutrition. 2009;18(3):326–34.

25. Petrova D, Bernabeu Litrán MA, García-Mármol E, Rodríguez-Rodríguez M, Cueto-Martín B, López-Huertas E, et al. Еffects of fortified milk on cognitive abilities in school-aged children: results from a randomized-controlled trial. European Journal of Nutrition. 2019;58(5):1863–72.

26. Costarelli L, Giacconi R, Malavolta M, Basso A, Piacenza F, DeMartiis M, et al. Effects of zinc-fortified drinking skim milk (as functional food) on cytokine release and thymic hormone activity in very old persons: A pilot study. Age. 2014;36(3):1421–31.

27. Sazawal S, Habib A, Dhingra U, Dutta A, Dhingra P, Sarkar A, et al. Impact of micronutrient fortification of yoghurt on micronutrient status markers and growth - a randomized double blind controlled trial among school children in Bangladesh. BMC Public Health. 2013;13:514.

28. Muthayya S, Eilander A, Transler C, Thomas T, van der Knaap HCM, Srinivasan K, et al. Effect of fortification with multiple micronutrients and n-3 fatty acids on growth and cognitive performance in Indian schoolchildren: the CHAMPION (Children’s Health and Mental Performance Influenced by Optimal Nutrition) Study. American Journal of Clinical Nutrition. 2009;89(6):1766–75.

29. Thomas T, Eilander A, Muthayya S, McKay S, Thankachan P, Theis W, et al. The effect of a 1-year multiple micronutrient or n-3 fatty acid fortified food intervention on morbidity in Indian school children. European Journal of Clinical Nutrition. 2012;66(4):452–8.

30. Engle-Stone R, Nankap M, Ndjebayi AO, Allen LH, Shahab-Ferdows S, Hampel D, et al. Iron, Zinc, Folate, and Vitamin B-12 Status Increased among Women and Children in Yaounde and Douala, Cameroon, 1 Year after Introducing Fortified Wheat Flour. Journal of Nutrition. 2017;147(7):1426–36.

31. Huo J, Sun J, Huang J, Li W, Wang L, Selenje L, et al. The effectiveness of fortified flour on micro-nutrient status in rural female adults in China. Asia Pacific Journal of Clinical Nutrition. 2011;20(1):118–24.

32. Huo J, Gary RG, Jian H, Jing S, Lijuan W, Lilian S, et al. Effectiveness of Fortified Flour for Enhancement of Vitamin and Mineral Intakes and Nutrition Status in Northwest Chinese Villages. Food and Nutrition Bulletin. 2012;33(2):161–8.

33. Jiang Z, Liang Q, Wang Y, Zheng X, Pei L, Zhang T, et al. Metabonomic study on women of reproductive age treated with nutritional intervention: Screening potential biomarkers related to neural tube defects occurrence. Biomedical Chromatography. 2011;25(7):767–74.

34. Stuetz W, Carrara VI, McGready R, Lee SJ, Erhardt JG, Breuer J, et al. Micronutrient status in lactating mothers before and after introduction of fortified flour: Cross-sectional surveys in Maela refugee camp. European Journal of Nutrition. 2012;51(4):425–34.

35. Hieu NT, Sandalinas F, De Sesmaisons A, Laillou A, Tam NP, Khan NC, et al. Multi-micronutrient-fortified biscuits decreased the prevalence of anaemia and improved iron status, whereas weekly iron supplementation only improved iron status in Vietnamese school children. British Journal of Nutrition. 2012;108(8):1419–27.

36. Nga TT, Wasantwisut E, Furr H, Wieringa FT, Winichagoon P, Dijkhuizen MA, et al. Multi-Micronutrient-Fortified Biscuits Decreased Prevalence of Anemia and Improved Micronutrient Status and Effectiveness of Deworming in Rural Vietnamese School Children. Journal of Nutrition. 2009;139(5):1013–21.

37. Nga TT, Winichagoon P, Dijkhuizen MA, Khan NC, Wasantwisut E, Wieringa FT. Decreased Parasite Load and Improved Cognitive Outcomes Caused by Deworming and Consumption of Multi-Micronutrient Fortified Biscuits in Rural Vietnamese Schoolchildren. American Journal of Tropical Medicine and Hygiene. 2011;85(2):333–40.

38. de Romana DL, Lonnerdal B, Brown KH. Absorption of zinc from wheat products fortified with iron and either zinc sulfate or zinc oxide. American Journal of Clinical Nutrition. 2003;78(2):279–83.

39. Sari DK, Marliyati SA, Kustiyah L, Khomsan A. Role of biscuits enriched with albumin protein from snakehead fish, zinc and iron on immune response of under five children. Pakistan Journal of Nutrition. 2014;13(1):28–32.

40. Romana DL de, Peerson JM, Krebs NF, Brown KH, Salazar M, Hambidge KM, et al. Longitudinal measurements of zinc absorption in Peruvian children consuming wheat products fortified with iron only or iron and 1 of 2 amounts of zinc. American Journal of Clinical Nutrition. 2005;81(3):637–47.

41. Aaron GJ, Ba Lo N, Hess SY, Guiro AT, Wade S, Brown KH. Plasma Zinc Concentration Increases within 2 Weeks in Healthy Senegalese Men Given Liquid Supplemental Zinc, but Not Zinc-Fortified Wheat Bread. The Journal of Nutrition. 2011 Jul 1;141(7):1369–74.

42. Badii A, Nekouei N, Fazilati M, Shahedi M, Badiei S. Effect of Consuming Zinc-fortified Bread on Serum Zinc and Iron Status of Zinc-deficient Women: A Double Blind, Randomized Clinical Trial. Int J Prev Med. 2012 Mar;3(Suppl 1):S124-130.

43. Hansen M, Samman S, Madsen LT, Jensen M, Sorensen SS, Sandstrom B. Folic acid enrichment of bread does not appear to affect zinc absorption in young women. American Journal of Clinical Nutrition. 2001;74(1):125–9.

44. Sandstrom B, Bjorn-Rasmussen E, Cederblad A, Arvidsson B. Zinc absorption from composite meals. I. The significance of wheat extraction rate, zinc, calcium, and protein content in meals based on bread. American Journal of Clinical Nutrition. 1980;33(4):739–45.

45. Saldamli I, Ozalp I, Kilic I, Koksel H, Ozboy O. Zinc-supplemented bread and its utilization in zinc deficiency. Cereal Chemistry. 1996;73(4):424–7.

46. Kiliç I, Ozalp I, Coskun T, Tokatli A, Emre S, Saldamli I, et al. The effect of zinc-supplemented bread consumption on school children with asymptomatic zinc deficiency. J Pediatr Gastroenterol Nutr. 1998;26(2):167–71.

47. Herman S, Griffin IJ, Suwarti S, Ernawati F, Permaesih D, Pambudi D, et al. Cofortification of iron-fortified flour with zinc sulfate, but not zinc oxide, decreases iron absorption in Indonesian children. American Journal of Clinical Nutrition. 2002;76(4):813–7.

48. Hambidge KM, Chavez MN, Brown RM, Walravens PA. ZINC NUTRITIONAL-STATUS OF YOUNG MIDDLE-INCOME CHILDREN AND EFFECTS OF CONSUMING ZINC-FORTIFIED BREAKFAST CEREALS. American Journal of Clinical Nutrition. 1979;32(12):2532–9.

49. Nieman DC, Henson DA, Sha W. Ingestion of micronutrient fortified breakfast cereal has no influence on immune function in healthy children: A randomized controlled trial. Nutrition Journal [Internet]. 2011;10(1). Available from: http://www.embase.com/search/results?subaction=viewrecord&from=export&id=L51384261

50. Ara G, Khanam M, Rahman AS, Islam Z, Farhad S, Sanin KI, et al. Effectiveness of micronutrient-fortified rice consumption on anaemia and zinc status among vulnerable women in Bangladesh. PLoS ONE [Internet]. 2019;14(1). Available from: https://www.scopus.com/inward/record.uri?eid=2-s2.0-85059829140&doi=10.1371%2fjournal.pone.0210501&partnerID=40&md5=f0a7361196c7c59ee37c587f6f6665fc

51. De Gier B, Ponce MC, Perignon M, Fiorentino M, Khov K, Chamnan C, et al. Micronutrient-fortified rice can increase hookworm infection risk: A cluster randomized trial. PLoS ONE [Internet]. 2016;11(1). Available from: https://www.scopus.com/inward/record.uri?eid=2-s2.0-84954091365&doi=10.1371%2fjournal.pone.0145351&partnerID=40&md5=82b0f1bd54675139a3da585d1a273698

52. Fiorentino M, Perignon M, Kuong K, de Groot R, Parker M, Burja K, et al. Effect of multi-micronutrient-fortified rice on cognitive performance depends on premix composition and cognitive function tested: results of an effectiveness study in Cambodian schoolchildren. Public Health Nutrition. 2018;21(4):816–27.

53. Kuong K, Tor P, Perignon M, Fiorentino M, Chamnan C, Berger J, et al. Multi-Micronutrient Fortified Rice Improved Serum Zinc and Folate Concentrations of Cambodian School Children. A Double-Blinded Cluster-Randomized Controlled Trial. Nutrients. 2019 Nov 20;11(12).

54. Della Lucia CM, Rodrigues KC, Rodrigues VC, Santos LL, Cardoso LM, Martino HS, et al. Diet Quality and Adequacy of Nutrients in Preschool Children: Should Rice Fortified with Micronutrients Be Included in School Meals? Nutrients. 2016;8(5).

55. Della Lucia CM, Santos LLM, Da Silva BP, Anunciação PC, Alfenas RCG, Franceschini SCC, et al. Impact of rice fortified with iron, zinc, thiamine and folic acid on laboratory measurements of nutritional status of preschool children. Ciencia e Saude Coletiva. 2017;22(2):583–92.

56. Dutta A. Assessment of fortification of Mid-Day Meal Programme in Dhenkanal, Odisha, 2016-2018 Evaluation Report - India [Internet]. World Food Programme; 2020 [cited 2020 Jul 7]. Available from: https://reliefweb.int/report/india/assessment-fortification-mid-day-meal-programme-dhenkanal-odisha-2016-2018-evaluation

57. Hackl L, Speich C, Zeder C, Sanchez-Ferrer A, Adelmann H, de Pee S, et al. Cold Extrusion but Not Coating Affects Iron Bioavailability from Fortified Rice in Young Women and Is Associated with Modifications in Starch Microstructure and Mineral Retention during Cooking. Journal of Nutrition. 2017;147(12):2319–25.

58. Ibnu IN, Thaha RM, Suriah. Effect of iron and zinc substance giving through fortification rice on stress level of school age children in islamic boarding school annihayahkarawang. Indian Journal of Public Health Research and Development. 2019;10(4):1071–7.

59. Pinkaew S, Winichagoon P, Hurrell RF, Wegmuller R. Extruded Rice Grains Fortified with Zinc, Iron, and Vitamin A Increase Zinc Status of Thai School Children When Incorporated into a School Lunch Program. Journal of Nutrition. 2013;143(3):362–8.

60. Sun J, Huang J, Huo JS. School food fortification improves nutrition status of students from poor migrant families. Annals of Nutrition and Metabolism. 2013;63:483.

61. Hettiarachchi M, Hilmers DC, Liyanage C, Abrams SA. Na2EDTA Enhances the Absorption of Iron and Zinc from Fortified Rice Flour in Sri Lankan Children. The Journal of Nutrition. 2004 Nov 1;134(11):3031–6.

62. Varea A, Malpeli A, Etchegoyen G, Vojkovic M, Disalvo L, Apezteguia M, et al. Short-Term Evaluation of the Impact of a Food Program on the Micronutrient Nutritional Status of Argentinean Children Under the Age of Six. Biological Trace Element Research. 2011;143(3):1337–48.

63. Varea A, Carmuega E, Pereyras S, Etchegoyen G, Gonz©Łlez HF, Vojkovic M, et al. Evaluation of the Impact of a Food Program on the Micronutrient Nutritional Status of Argentinean Lactating Mothers [electronic resource]. Biological Trace Element Research. 2012;150(1–3):103–8.

64. Hotz C, DeHaene J, Woodhouse LR, Villalpando S, Rivera JA, King JC. Zinc absorption from zinc oxide, zinc sulfate, zinc oxide + EDTA, or sodium-zinc EDTA does not differ when added as fortificants to maize tortillas. Journal of Nutrition. 2005;135(5):1102–5.

65. Rosado JL, Diaz M, Munoz E, Westcott JL, Gonzalez KE, Krebs NF, et al. Bioavailability of zinc oxide added to corn tortilla is similar to that of zinc sulfate and is not affected by simultaneous addition of iron. Food and Nutrition Bulletin. 2012;33(4):261–6.

66. Chen K, Li TY, Chen L, Qu P, Liu YX. Effects of vitamin A, vitamin A plus iron and multiple micronutrient-fortified seasoning powder on preschool children in a suburb of Chongqing, China. Journal of Nutritional Science and Vitaminology. 2008;54(6):440–7.

67. Chen K, Wei X, Qu P, Liu Y, Zhang X, Li T, et al. Effect of vitamin A, vitamin A plus iron and multiple micronutrient-fortified seasoning powder on infectious morbidity of preschool children [electronic resource]. Nutrition. 2011;27(4):428–34.

68. Winichagoon P, Bailey KB, Manger MS, Gibson RS, Wasantwisut E, Boonpraderm A, et al. A Multimicronutrient-Fortified Seasoning Powder Enhances the Hemoglobin, Zinc, and Iodine Status of Primary School Children in North East Thailand: A Randomized Controlled Trial of Efficacy. Journal of Nutrition. 2006;136(6):1617–23.

69. Manger MS, McKenzie JE, Winichagoon P, Gray A, Chavasit V, Pongcharoen T, et al. A micronutrient-fortified seasoning powder reduces morbidity and improves short-term cognitive function, but has no effect on anthropometric measures in primary school children in northeast Thailand: A randomized controlled trial. American Journal of Clinical Nutrition. 2008;87(6):1715–22.

70. Vinodkumar M, Erhardt JG, Rajagopalan S. Impact of a multiple-micronutrient fortified salt on the nutritional status and memory of schoolchildren. International Journal for Vitamin and Nutrition Research. 2009;79(5–6):348–61.

71. Ohiokpehai O, David DM, Kamau J. Serum zinc levels of school children on a corn-soy blend feeding trial in primary schools in Suba district, Kenya. Journal of Applied Biosciences. 2009 May 8;17:904–12.

72. An H, Yin S, Xu Q. [Effects of supplementing calcium, iron and zinc on the fetus development and growth during pregnancy]. Zhonghua Yu Fang Yi Xue Za Zhi. 2001 Nov;35(6):370–3.

1. References listed in the same row are different articles reporting separate outcomes but based on the same study [↑](#endnote-ref-1)
2. References appear in the following hierarchy: food category, alphabetical order, year of publication [↑](#endnote-ref-2)
3. UNICEF regional classifications [↑](#endnote-ref-3)
4. Where described as ‘Beverage’, the fortified food is a drink produced by the investigators for the study (as opposed to a drink such as milk). [↑](#endnote-ref-4)
5. Participant ages is presented as a range across intervention arms or equal to/greater than, unless the authors only provided mean age, separated by intervention and control group [↑](#endnote-ref-5)
6. High adherence defined as: 80% or more of the participants adhered to the protocols for assigned interventions [↑](#endnote-ref-6)
7. Mean age [↑](#endnote-ref-7)
8. Mean age [↑](#endnote-ref-8)
9. Mean age [↑](#endnote-ref-9)
