# Supplementary material for: Effects of Foods Fortified with Zinc, Alone or Cofortified with Multiple Micronutrients, on Health and Functional Outcomes: A Systematic Review and Meta-Analysis
Source: Adv Nutr. 2021 Jun 24;12(5):1821–37. doi: 10.1093/advances/nmab065 (PMC8483949; doi:10.1093/advances/nmab065)
Supplement: nmab065_Supplemental_Files [file nmab065_supplemental_files.zip › Supplemental Table 10. Zinc absorption.docx]

**TABLE S10: Fractional and total absorbed zinc of zinc-fortified foods (*n*=6)^[[1]](#endnote-1),^^[[2]](#endnote-2)^**

| Reference | *n* | Population^[[3]](#endnote-3)^ | Study design | Fortified food^[[4]](#endnote-4)^, zinc compound | Dose (mg/d)^[[5]](#endnote-5)^ | FAZ (mean ± SD)^[[6]](#endnote-6)^ | | TAZ (mean ± SD)^6^ | |
| --- | --- | --- | --- | --- | --- | --- | --- | --- | --- |
|  |  |  |  |  |  | *Control/Baseline* | *Intervention/End line* | *Control/Baseline* | *Intervention/End line* |
| Hansen et al. 2001^[[7]](#endnote-7)^ (1)  *Denmark* | 15 | WRA, 22-33 y | Whole-body counting, cross-over | Wheat flour: buns (co-fortified with or without folic acid) (zinc chloride)  Control: buns fortified with low zinc | Low zinc: 1.2  High zinc: 3.0 | Low zinc:  0.40 ± 0.15 | High zinc:  0.25 ± 0.09 | Low zinc:  0.48 ± 0.18 | High zinc:  0.73 ± .25 |
| López de Romaña et al. 2005 (2)  *Peru* | 41 | Children, 3-4 y | Dual stable isotope tracer | Wheat flour: noodles and biscuits fortified at 30 mg/kg or 90 mg/kg of flour (zinc sulfate)  Control: non-fortified noodles and biscuits | 30 mg/kg: 3  90 mg/kg: 9 | 0.341 ± 0.111^a^ | 3 mg/d:  0.237 ± 0.052^b^  9 mg/d:  0.133 ± 0.041^c^ | 0.71 ± 0.18^x^ | 3 mg/d:  1.11 ± 0.21^y^  9 mg/d:  1.34 ± 0.47^y^ |
| Méndez et al. 2015 (3)  *Mexico* | 14 | WRA, 12-16 y | Dual stable isotope tracer, pre-post | Milk, liquid (NR)  Comparison: baseline dietary intake | 6.6 | 0.17 ± 0.02 | 0.215 ± 0.11^[[8]](#endnote-8)^ | 1.06 ± 0.14^x^ | 3.09 ± 0.27^y^ |
| Rosado et al. 2012 (4)  *Mexico* | 10 | WRA, 21-51 y | Single isotope tracer balance study, cross-over | Maize flour: tortillas fortified with zinc oxide or zinc sulfate at 20 mg/kg  Control: non-fortified tortillas | Zinc oxide: 6.4  Zinc sulfate: 6.6 | 0.35 ± 0.22 | Zinc oxide:  0.36 ± 0.16  Zinc sulfate:  0.37 ± 0.22 | 2.3 ± 0.5^x^ | Zinc oxide:  4.8 ± 2.53 ^y^  Zinc sulfate:  5.0 ± 3.16^y^ |
| Ruz et al. 2005 (5)  *Chile* | 14 | WRA, 41.9 y | Dual stable isotope tracer, cross-over | Milk, powdered (zinc oxide) | 1.92 | 0.29 ± 0.09 | 0.30 ± 0.09 | 0.43 ± 0.14 | 0.57 ± 0.16 |
| Sandström et al. 1980^[[9]](#endnote-9)^ (6)  *Sweden* | 66 | Adults, 19-61 y | Dual stable isotope tracer | Wheat flour: white or whole-meal bread (zinc chloride)  Control: non-fortified white or whole-meal bread | White bread: 3.1  Whole-meal bread: 2.2 | White bread:  0.382 ± 0.071  Whole-meal bread:  0.166 ± 0.046 | White bread:  0.132 ± 0.050  Whole-meal bread:  0.082 ± 0.018 | White bread:  0.15 ± 0.03^a^  Whole-meal bread:  0.22 ± 0.06^x^ | White bread:  0.48 ± 0.18^b^  Whole-meal bread:  0.29 ± 0.06^y^ |

**References:**

1. Hansen M, Bæch SB, Thomsen AD, Tetens I, Sandström B. Long-term intake of iron fortified wholemeal rye bread appears to benefit iron status of young women. Journal of Cereal Science. 2005;42:165–71.

2. López de Romaña D, Peerson JM, Krebs NF, Brown KH, Salazar M, Hambidge KM, Penny ME. Longitudinal measurements of zinc absorption in Peruvian children consuming wheat products fortified with iron only or iron and 1 of 2 amounts of zinc. American Journal of Clinical Nutrition. 2005;81:637–47.

3. Méndez RO, Hambidge M, Baker M, Salgado SA, Ruiz J, Garcia HS, Calderon de la Barca AM. Zinc Absorption from Fortified Milk Powder in Adolescent Girls. Biol Trace Elem Res. 2015;168:61–6.

4. Rosado JL, Diaz M, Munoz E, Westcott JL, Gonzalez KE, Krebs NF, Caamano MC, Hambidge M. Bioavailability of zinc oxide added to corn tortilla is similar to that of zinc sulfate and is not affected by simultaneous addition of iron. Food and Nutrition Bulletin. 2012;33:261–6.

5. Ruz M, Codoceo J, Inostroza J, Rebolledo A, Krebs NF, Westcott JE, Sian L, Hambidge KM. Zinc absorption from a micronutrient-fortified dried cow’s milk used in the Chilean National Complementary Food Program. Nutrition Research. 2005;25:1043–8.

6. Sandstrom B, Bjorn-Rasmussen E, Cederblad A, Arvidsson B. Zinc absorption from composite meals. I. The significance of wheat extraction rate, zinc, calcium, and protein content in meals based on bread. American Journal of Clinical Nutrition. 1980;33:739–45.

1. Papers organized by alphabetical order. [↑](#endnote-ref-1)
2. Abbreviations (alphabetical): mg/kg, milligrams of zinc per kilogram of fortified food; NR, not reported; WRA, women of reproductive age. [↑](#endnote-ref-2)
3. Age as reported by authors, in range or mean/median [↑](#endnote-ref-3)
4. Staple food: product prepared with that staple food (in the case of maize and wheat flours), and fortification level (if reported). [↑](#endnote-ref-4)
5. All doses refer to extrinsically added zinc [↑](#endnote-ref-5)
6. Results converted to mean ± SD if reported otherwise. Different superscripts between interventions within the same row (a, b, c or x, y, z) indicate statistically significant differences (as reported by study authors). Results without superscripts indicate no statistical test conducted. [↑](#endnote-ref-6)
7. No significant difference in FAZ and TAZ due to the inclusion of folic acid; therefore, the no-folic acid and folic acid groups were combined using the following formula: <https://handbook-5-1.cochrane.org/chapter_7/7_7_3_8_combining_groups.htm> [↑](#endnote-ref-7)
8. Study reported the separate meal and fortified milk FAZ. This combined FAZ is calculated by the review authors using the following formula: <https://handbook-5-1.cochrane.org/chapter_7/7_7_3_8_combining_groups.htm> [↑](#endnote-ref-8)
9. Study authors originally report mean (range). For comparability in meta-analyses, range converted to standard deviation using [Wan X, et al. 2014](https://bmcmedresmethodol.biomedcentral.com/articles/10.1186/1471-2288-14-135#Sec17) [↑](#endnote-ref-9)
