# Supplementary material for: Effects of Foods Fortified with Zinc, Alone or Cofortified with Multiple Micronutrients, on Health and Functional Outcomes: A Systematic Review and Meta-Analysis
Source: Adv Nutr. 2021 Jun 24;12(5):1821–37. doi: 10.1093/advances/nmab065 (PMC8483949; doi:10.1093/advances/nmab065)
Supplement: nmab065_Supplemental_Files [file nmab065_supplemental_files.zip › Supplemental Table 11. Iron and copper outcomes.docx]

**Table S11. Effect of foods fortified with zinc, alone or co-fortified with multiple micronutrients, on plasma/serum ferritin and copper biomarker concentrations**

| Reference  *Study location* | *n*^[[1]](#endnote-1)^ | Population^[[2]](#endnote-2)^ | Zinc fortified food | Zinc dose, duration^[[3]](#endnote-3)^ | Iron dose  Zinc:iron molar ratio | Plasma/serum zinc  (µg/dL) | Plasma/serum ferritin  (µg/L) | Plasma/serum copper  (µg/dL) |
| --- | --- | --- | --- | --- | --- | --- | --- | --- |
| Badii et al. 2011 (6)  *Iran* | 75 | 32.2 y  NPNL women | Bread, Iranian flatbread  (Taftoon) | 9.15 mg/d or 16.16 mg/d, 1 mo | NA | 50 mg/kg  Baseline: 65.06 ± 0.95  End line: 86.8 ± 10.78  100 mg/kg  Baseline: 66.53 ± 3.17  End line: 78.73 ± 8.1  C:  Baseline: 65.53 ± 3.38  End line: 67.73 ± 2.17 | 50 mg/kg  Baseline: 7.8 ± 3.179  End line: 13.9.87 ± 2.766  100 mg/kg  Baseline: 8.0.13 ± 3.205  End line: 12.4.13 ± 3.041  C:  Baseline: 8.3.26 ± 2.148  End line: 8.7.06 ± 0.254 | NM |
| Costarelli et al. 2014 (1)  *Italy* | 21 | ≥ 82 y  Healthy | Milk, skim | 4 mg/d, 2 mo | NA | End line  C: 89.28 ± 33.85  I: 101.24 ± 40.73 | NR | End line  C: 112.52 ± 22.13  I: 118.23 ± 20.96 |
| Hambidge et al. 1979 (2)  *United States* | 93 | 33-90 mo  Healthy children | Cereal, ready to eat | 2.57 mg/d  9 mo | NA | C: -8.7 ± 3^[[4]](#endnote-4)^  I: -2.3 ± 2.3^5^ | NR | C: -6.18 ± 4.75^5^  I: -13.7 ± 5.45^5^ |
| Hettiarachchi et al. 2004  *Sri Lanka* | 53 | 7-10 y | Rice flour | 4.5 mg/d, 1 mo | 4.5 mg/d  0.85 | End line:  C: 12.3 ± 2.9  I: 13.0 ± 2.5 | End line:  C: 48.5 ± 25.1  I: 49.5 ± 21.2 | NM |
| Kiliç et al. 1998 (5)  *Turkey* | 24 | 7-11 y  Healthy children | Wheat flour, bread | 2 mg/kg of body weight/d, 3 mo  54.5 mg/d^[[5]](#endnote-5)^ | NA | Baseline  C: 58.9 ± 2.7  I: 60.8 ± 3.6  End line  C: 62.9 ± 3.4  I: 81.5 ± 9.1 | Baseline  C: 32.2 ± 6.60  I: 28.8 ± 11.0  End line  C: 24.1 ± 11.7  I: 30.4 ± 9.7 | Baseline  C: 92.95 ± 6.61  I: 94.66 ± 35.32  110 d  C: 94.35 ± 16.14  I: 84.56 ± 15.63 |
| López de Romaña et al. 2005 (3)  *Peru* | 31 | 3-4 y  Anemic children  at high risk of  zinc deficiency | Wheat flour, biscuits and noodles | 0 mg/d  3 mg/d  9 mg/d  2.3 mo | 3 mg/d (all arms)  3 mg/d: 0.85  9 mg/d: 2.56 | 0 mg/kg flour:  Baseline: 70.8 ± 8.8  End line: 77.6 ± 12.3  30 mg/kg flour  Baseline: 77.4 ± 13.1  End line: 76.2 ± 8.2  90 mg/kg flour  Baseline: 77.6 ± 16.2  End line: 82.4 ± 9.9 | 0 mg/kg flour:  Baseline: 28.07 ± 21.20  End line: 21.10 ± 20.30  30 mg/kg flour  Baseline: 16.34 ± 17.40  End line: 15.79 ± 13.00  90 mg/kg flour  Baseline: 19.48 ± 19.40  End line: 13.73 ± 14.00 | NM |
| Stuetz et al. 2012 (4)  *Thailand*  *(Maela refugee camp)* | 86 (pre)  98 (post) | Pre: 16-41 y  Post: 17-46 y | Wheat flour | 2.7 mg/d, 3-4 mo | NA | Baseline:  61.50 ± 12.0  End line:  66.30 ± 15.40 | NR | Baseline:  119.9 ± 21.6  End line:  117.3 ± 17.6 |

**References**

1. Costarelli L, Giacconi R, Malavolta M, Basso A, Piacenza F, DeMartiis M, Giannandrea E, Renieri C, Busco F, Galeazzi R, et al. Effects of zinc-fortified drinking skim milk (as functional food) on cytokine release and thymic hormone activity in very old persons: A pilot study. Age. Kluwer Academic Publishers; 2014;36:1421–31.

2. Hambidge KM, Chavez MN, Brown RM, Walravens PA. ZINC NUTRITIONAL-STATUS OF YOUNG MIDDLE-INCOME CHILDREN AND EFFECTS OF CONSUMING ZINC-FORTIFIED BREAKFAST CEREALS. American Journal of Clinical Nutrition. 1979;32:2532–9.

3. López de Romaña D, Peerson JM, Krebs NF, Brown KH, Salazar M, Hambidge KM, Penny ME. Longitudinal measurements of zinc absorption in Peruvian children consuming wheat products fortified with iron only or iron and 1 of 2 amounts of zinc. American Journal of Clinical Nutrition. 2005;81:637–47.

4. Stuetz W, Carrara VI, McGready R, Lee SJ, Erhardt JG, Breuer J, Biesalski HK, Nosten FH. Micronutrient status in lactating mothers before and after introduction of fortified flour: Cross-sectional surveys in Maela refugee camp. European Journal of Nutrition. 2012;51:425–34.

5. Kiliç I, Ozalp I, Coskun T, Tokatli A, Emre S, Saldamli I, Koksel H, Ozboy O. The effect of zinc-supplemented bread consumption on school children with asymptomatic zinc deficiency. J Pediatr Gastroenterol Nutr. 1998;26:167–71.

6. Badii A, Nekouei N, Fazilati M, Shahedi M, Badiei S. Effect of Consuming Zinc-fortified Bread on Serum Zinc and Iron Status of Zinc-deficient Women: A Double Blind, Randomized Clinical Trial. Int J Prev Med. 2012;3:S124-130.

1. Abbreviations: C, control; I, intervention; NA, not applicable; NM, not measured; NPNL, nonpregnant, nonlactating; NR, not reported

   Sample size included in analysis [↑](#endnote-ref-1)
2. Population characteristics included are age and health status [↑](#endnote-ref-2)
3. Durations were converted to months using the following methodology: 4 weeks=1 month, 30 days=1 month, 1 year=12 months [↑](#endnote-ref-3)
4. Reported as change value [↑](#endnote-ref-4)
5. Calculated by review authors; authors stated a fortification level of 3 grams/1kg and an intended daily dose of 2 mg/kg of body weight. Dose/day was calculated using the average of the treatment and control groups’ weight at baseline (27.6 kg for treatment, 26.8 kg for control) [↑](#endnote-ref-5)
