# Supplementary material for: Effects of Foods Fortified with Zinc, Alone or Cofortified with Multiple Micronutrients, on Health and Functional Outcomes: A Systematic Review and Meta-Analysis
Source: Adv Nutr. 2021 Jun 24;12(5):1821–37. doi: 10.1093/advances/nmab065 (PMC8483949; doi:10.1093/advances/nmab065)
Supplement: nmab065_Supplemental_Files [file nmab065_supplemental_files.zip › Supplemental Table 12. Hair and urine zinc.docx]

**Table S12. Effect of foods fortified with zinc, alone or co-fortified with multiple micronutrients, on urine and hair zinc concentrations (*n*=2)**

| Reference  *Study location* | *n*^[[1]](#endnote-1)^ | Population  characteristics^[[2]](#endnote-2)^ | Zinc fortified food | Zinc dose, duration^[[3]](#endnote-3)^ | Control group food | Urinary zinc  (µg/24 hr) | Hair zinc  (µg/g) |
| --- | --- | --- | --- | --- | --- | --- | --- |
| Hambidge et al. 1979 (1)  *United States* | 93 | 33-90 mo  Healthy | Cereal, ready to eat | 2.57 mg/d, 9 mo | Non zinc-fortified  cereal | C: 51.5 ± 115.02^[[4]](#endnote-4)^  I: 68.7 ± 128.02 | C: 27.4 ± 39.53^[[5]](#endnote-5)^  I: 35.6 ± 47.48 |
| Tukvadze & Kverenchkhiladze 2013 (2)  *Georgia* | 36 | 11-14 y  Healthy | Tea | NR, 3 mo | NA^[[6]](#endnote-6)^ | Boys:  Baseline:  220.0 ± 30.0  End line:  315.00 ± 30.0  Girls:  Baseline:  250.0 ± 20.0  End line:  333.0 ± 25.0 | Boys:  Baseline:  135.0±12.4  End line:  129.01±14.0  Girls:  Baseline:  160.0±20.0  End line:  157.8±15.0 |

**References:**

1. Hambidge KM, Chavez MN, Brown RM, Walravens PA. ZINC NUTRITIONAL-STATUS OF YOUNG MIDDLE-INCOME CHILDREN AND EFFECTS OF CONSUMING ZINC-FORTIFIED BREAKFAST CEREALS. American Journal of Clinical Nutrition. 1979;32:2532–9.

2. Tukvadze S, Kverenchkhiladze R. Inclusion of zinc fortified tea into the children’s diet and its hygienicassessment. Georgian medical news. 2013;53–6.

1. Abbreviations: C, control; I, intervention; NA, not applicable; NR, not reported.

   Sample size included in analysis [↑](#endnote-ref-1)
2. Population characteristics included are age and health status [↑](#endnote-ref-2)
3. Durations were converted to months using the following methodology: 4 weeks=1 month, 30 days=1 month, 1 year=12 [↑](#endnote-ref-3)
4. Reported as change value [↑](#endnote-ref-4)
5. Reported as change value [↑](#endnote-ref-5)
6. Pre/post; all participants received the intervention [↑](#endnote-ref-6)
