# Supplementary material for: Effects of Foods Fortified with Zinc, Alone or Cofortified with Multiple Micronutrients, on Health and Functional Outcomes: A Systematic Review and Meta-Analysis
Source: Adv Nutr. 2021 Jun 24;12(5):1821–37. doi: 10.1093/advances/nmab065 (PMC8483949; doi:10.1093/advances/nmab065)
Supplement: nmab065_Supplemental_Files [file nmab065_supplemental_files.zip › Supplemental Table 13. Immune function.docx]

**Table S13: Effect of foods fortified with zinc, alone or co-fortified with multiple micronutrients, on immune function biomarkers (*n*=6)^[[1]](#endnote-1),^^[[2]](#endnote-2)^**

| Reference  *Study location* | *n*^[[3]](#endnote-3)^ | Population  characteristics^[[4]](#endnote-4)^ | Zinc fortified food | Zinc dose, duration^[[5]](#endnote-5)^ | Control group food | Immune Function | |
| --- | --- | --- | --- | --- | --- | --- | --- |
|  |  |  |  |  |  | *Baseline* | *End line* |
| Kiliç et al. 1998 (1)  *Turkey* | 24 | 7-11 y  Healthy | Bread | 54.4 mg/d^[[6]](#endnote-6)^, 3 mo | Non-fortified bread | Delayed Hypersensitivity Response to PPD (%)  C:25  I:33  **Delayed Hypersensitivity Response to PHA (%)**  C:25  I: 16.6  **Delayed Hypersensitivity Response to Candida (%)**  C: 50  I: 41.6  **Total lymphocyte (mm^3^)**  C:2367.3±528.8  I:2041±543 | **Delayed Hypersensitivity Response to PPD (%)**  C:33 I: 66  **Delayed Hypersensitivity Response to PHA (%)**  C: 33 I: 66  **Delayed Hypersensitivity Response to Candida (%)**  C:66 I: 83.3  **Total lymphocyte (mm^3^)**  C:2288.3±716.3 I: 2467±772.4 |
| Nieman et al. 2011 (2)  *United States* | 65 | 7-13 y  Heathy | Cereal, extruded puffed  corn cereal by General Mills  Co-intervention:  Pneumococcal  vaccine | 0 mg/d, 12.5 mg/d, 25 mg/d, 2 mo | Non-fortified cereal | **NK lymphocytes (10^9/L)**  Low: 0.39 ± 0.28  Medium: 0.49 ± 0.34  High: 0.35 ± 0.18  **NKCA 40:1 E:T (% lysis)**  Low: 44.7 ± 16.8  Medium: 47.6 ± 14.1  High: 48.4 ± 16.3  **NKCA 20:1 E:T (% lysis)**  Low: 33.2 ± 16.3  Medium: 33.6 ± 12.7  High: 38.1 ± 17.1  **Saliva IgA concentration (ug/mL)**  Low: 333 ± 181  Medium: 292 ± 143  High: 315 ± 159  **Oxidative burst activity (DCF+ Granulocytes)**  Low: 192 ± 65  Medium: 184 ± 79  High: 213 ± 77  **Phagocytosis (FITC+ Granulocytes)**  Low: 459 ± 92  Medium: 426 ± 109  High: 442 ± 108  **DTH skin response (mm)**  Low 23.5 ± 10.1  Medium: 22.5 ± 11.3  High: 24.3 ± 13.7 | **NK lymphocytes (10^9/L)**  Low: 0.23 ± 0.13  Medium: 0.36 ± 0.20  High: 0.29 ± 0.14  **NKCA 40:1 E:T (% lysis)**  Low: 48.8 ± 16.2  Medium: 47.1 ± 16.8  High: 48.6 ± 16.3  **NKCA 20:1 E:T (% lysis)**  Low: 37.0 ± 16.8  Medium: 35.5 ± 12.7  High: 36.3 ± 13.2  **Saliva IgA concentration (ug/mL)**  Low: 314 ± 152  Medium: 302 ± 151  High: 289 ± 158  **Oxidative burst activity (DCF+ Granulocytes)**  Low: 136 ± 58  Medium: 171 ± 119  High: 141 ± 66  **Phagocytosis (FITC+ Granulocytes)**  Low: 424 ± 84  Medium: 447 ± 170  High: 431 ± 93  **DTH skin response (mm)**  Low: 12.8 ± 7.5  Medium: 16.4 ± 12.4  High: 12.6 ± 6.3 |
| Thomas et al. 2012 (3)  *India* | 546 | 6-10 y  Healthy | Wheat biscuit &  Milk powder  Co-fortification  with n-3 fatty  acids | Dose:  High MMN/high n-3:  10.5 mg/d  High MMN/low n-3:  10.5 mg/d  Low MMN/high n-3:  1.7 mg/d  Low MMN/low n-3:  1.7 mg/d  12 mo |  | *Vaccine response %:*  high MNN: 37  low MNN 36  high n-3: 37  high n-2: 38  *anti-HBsAg antibody titres (25th and 75^th^ percentiles)*  High MMN/high n-3: 2 and 56  High MMN/low n-3: 2 and 36  Low MMN/high n-3: 2 and 45  Low MMN/low n-3: 2 and 62 |  |
| Wibowo et al. 2016 (4)  *Indonesia* | 104 | 18-35 y  Pregnant women | Milk powder | 10.5 mg/d, 9 mo | Non-fortified milk powder | **IL-6, pg/mL**  1^st^ Trimester  C: 1.4 (0.6-4.1)  I:1.6 (0.4-3.8)  **TNF-α**  1^st^ Trimester  C: 1.1 (0.2-6.7)  I: 1.2±0.3 | **IL-6, pg/mL**  3^rd^ Trimester  C:1.8 (0.7-10.6)  I: 1.8 (0.8-13.8)  **TNF-α**  3^rd^ Trimester  C: 1.3 (0.7-4.8)  I: 1.5 (0.7-2.0) |
| Costarelli et al. 2014 (5)  *Italy* | 21 | ≥ 82 y  Healthy | Milk, skim | 4 mg/d, 2 mo | Non-fortified milk | *Cytokine production*  *(pg/mL)*  **IL1-α**  C:2.88±0.23  I: 2.10±0.28*  **IL1-β**  C:4.90±2.06  I: 4.48±1.15  **IL-2**  C:18.30±4.19  I: 18.42±4.66  **IL-10**  C:10.38±1.52  I: 14.90±1.50*  **IL-12p70**  C: 1.46±0.20  I: 2.39±0.35*  **IL-6**  C:167.85±28.89  I: 164.76±23.71  **IFN-γ**  C: 4.61±0.40  I: 5.98±0.31**  **TNF-α**  C: 18.00±4.70  I: 17.49±3.70 |  |
| Sari et al. 2014 (6)  *Indonesia* | NR | 4-5 y  Low weight | Biscuit, fish  protein | 3.81±1.26 mg/d, 2 mo | Milk biscuit | IgG  (mg/mL)  C: -60.31 ± 81.76  I: 0.88 ± 0.58*  Albumin  (g/dL)  C: 0.05 ± 0.13  I: 0.48 ± 0.32* |  |

**References:**

1. Kiliç I, Ozalp I, Coskun T, Tokatli A, Emre S, Saldamli I, Koksel H, Ozboy O. The effect of zinc-supplemented bread consumption on school children with asymptomatic zinc deficiency. J Pediatr Gastroenterol Nutr. 1998;26:167–71.

2. Nieman DC, Henson DA, Sha W. Ingestion of micronutrient fortified breakfast cereal has no influence on immune function in healthy children: A randomized controlled trial. Nutrition Journal [Internet]. 2011;10. Available from: http://www.embase.com/search/results?subaction=viewrecord&from=export&id=L51384261

3. Thomas T, Eilander A, Muthayya S, McKay S, Thankachan P, Theis W, Gandhe A, Osendarp SJM, Kurpad AV. The effect of a 1-year multiple micronutrient or n-3 fatty acid fortified food intervention on morbidity in Indian school children. European Journal of Clinical Nutrition. 2012;66:452–8.

4. Wibowo N, Bardosono S, Irwinda R. Effects of Bifidobacterium animalis lactis HN019 (DR10TM), inulin, and micronutrient fortified milk on faecal DR10TM, immune markers, and maternal micronutrients among Indonesian pregnant women. Asia Pacific Journal of Clinical Nutrition. HEC Press; 2016;25:S102–10.

5. Costarelli L, Giacconi R, Malavolta M, Basso A, Piacenza F, DeMartiis M, Giannandrea E, Renieri C, Busco F, Galeazzi R, et al. Effects of zinc-fortified drinking skim milk (as functional food) on cytokine release and thymic hormone activity in very old persons: A pilot study. Age. Kluwer Academic Publishers; 2014;36:1421–31.

6. Sari DK, Marliyati SA, Kustiyah L, Khomsan A. Role of biscuits enriched with albumin protein from snakehead fish, zinc and iron on immune response of under five children. Pakistan Journal of Nutrition. Asian Network for Scientific Information; 2014;13:28–32.

1. **P*<0.05

   ** *P* <0.01

   Papers organized by dose (descending order) [↑](#endnote-ref-1)
2. Abbreviations (alphabetical): C, Control; I, Intervention; NR, Not Reported [↑](#endnote-ref-2)
3. Sample size included in analysis [↑](#endnote-ref-3)
4. Population characteristics included are age and health status [↑](#endnote-ref-4)
5. Durations were converted to months using the following methodology: 4 weeks=1 month, 30 days=1 month, 1 year=12 [↑](#endnote-ref-5)
6. Calculated by review authors; authors stated a fortification level of 3 grams/1kg and an intended daily dose of 2 mg/kg of body weight. Dose/day was calculated using the average of the treatment and control groups’ weight at baseline (27.6 kg for treatment, 26.8 kg for control) [↑](#endnote-ref-6)
