# Supplementary material for: Effects of Foods Fortified with Zinc, Alone or Cofortified with Multiple Micronutrients, on Health and Functional Outcomes: A Systematic Review and Meta-Analysis
Source: Adv Nutr. 2021 Jun 24;12(5):1821–37. doi: 10.1093/advances/nmab065 (PMC8483949; doi:10.1093/advances/nmab065)
Supplement: nmab065_Supplemental_Files [file nmab065_supplemental_files.zip › Supplemental Table 14. Plasma fatty acids.docx]

**Table S14. Effect of foods fortified with zinc, alone or co-fortified with multiple micronutrients, on plasma fatty acids**

| Reference  *study location* | *n*^[[1]](#endnote-1)^ | Population  characteristics^[[2]](#endnote-2)^ | Zinc fortified food | Zinc dose, duration^[[3]](#endnote-3)^ | Control group food | Plasma Fatty Acids | |
| --- | --- | --- | --- | --- | --- | --- | --- |
|  |  |  |  |  |  | *Baseline* | *End line* |
| Petrova et al. 2019 (1)  *Spain* | 103 | 8-14 y  Healthy | Milk  co-fortification  with poly unsaturated fatty acids | 13.5 mg/d,  5 mo | Non-fortified milk | **Plasma DHA**  C: 2.02 ± 0.09  I: 1.97 ± 0.09 | **Plasma DHA**  End line:  C: 1.88 ± 0.07  I: 2.43 ± 0.07** |
| Wibowo et al. 2016 (2)  *Indonesia* | 104 | 18-35 y  Pregnant women | Milk powder  co-fortification with poly unsaturated fatty acids | 10.5 mg/d, 9 mo | Non-fortified milk powder | **Total FFA**  C: 2,608 (238-10,473)  I: 2,674 ± 1446  **Linoleic**  C: 2,064 (120-8,987)  I: 2,115 ± 1,283  **Arachidonic Acid**  C: 271 ± 135  I: 279 (44.9-993)  **DHA**  C: 12.9 (1.2-96.4)  I: 16.0 (3.1-98.4) | **Total FFA**  C: 3,847 (582-14,004)  I: 4,115 ± 2,533  **Linoleic**  C: 3,055 (232-11,817)  I: 3,115 ± 2,286  **Arachidonic Acid**  C:257 ± 163  I: 248 ± 139  **DHA**  C: 14.0 (6.6-51.8)  I: 12.9 ± 7.0** |
| Muthayya et al. 2009 (3)  *India* | 550 | 6-10 y  Healthy | Wheat biscuit &  milk powder ǂ  co-fortification  with n-3 fatty  acids | 10.5 mg/d or 1.7mg/d, 12 mo | NA^[[4]](#endnote-4)^ | **Total n-3 (% of total fatty acids)**  High MMN/high n-3: 3.6 ± 0.8  High MMN/low n-3: 3.6 ± 0.9  Low MMN/high n-3: 3.6 ± 1.0  Low MMN/low n-3: 3.9 ± 2.6  **ALA (% of total fatty acids)**  High MMN/high n-3: 0.25 ± 0.18  High MMN/low n-3: 0.25 ± 0.16  Low MMN/high n-3: 0.26 ± 0.17  Low MMN/low n-3: 0.26 ± 0.17  **EPA (% of total fatty acids)**  High MMN/high n-3: 0.17 ± 0.07  High MMN/low n-3: 0.17 ± 0.08  Low MMN/high n-3: 0.18 ± 0.10  Low MMN/low n-3: 0.18 ± 0.07  **DHA (% of total fatty acids)**  High MMN/high n-3: 3.2 ± 0.7  High MMN/low n-3: 3.2 ± 0.8  Low MMN/high n-3: 3.2 ± 0.9  Low MMN/low n-3: 3.3 ± 0.9 | **Total n-3 (% of total fatty acids)**  High MMN/high n-3: 5.7 ± 1.3  High MMN/low n-3: 4.1 ± 0.8  Low MMN/high n-3: 5.8 ± 1.2  Low MMN/low n-3: 4.1 ± 1.1  **ALA (% of total fatty acids)**  High MMN/high n-3: 0.23 ± 0.10  High MMN/low n-3: 0.22 ± 0.08  Low MMN/high n-3: 0.23 ± 0.99  Low MMN/low n-3: 0.21 ± 0.07  **EPA (% of total fatty acids)**  High MMN/high n-3: 0.35 ± 0.16  High MMN/low n-3: 0.25 ± 0.14  Low MMN/high n-3: 0.39 ± 0.19  Low MMN/low n-3: 0.26 ± 0.18  **DHA (% of total fatty acids)**  High MMN/high n-3: 5.2 ± 1.2  High MMN/low n-3: 3.6 ± 0.8  Low MMN/high n-3: 5.2 ± 1.2  Low MMN/low n-3: 3.6 ± 1.0 |

**References:**

1. Petrova D, Bernabeu Litrán MA, García-Mármol E, Rodríguez-Rodríguez M, Cueto-Martín B, López-Huertas E, Catena A, Fonollá J. Еffects of fortified milk on cognitive abilities in school-aged children: results from a randomized-controlled trial. European Journal of Nutrition. 2019;58:1863–72.

2. Wibowo N, Bardosono S, Irwinda R. Effects of Bifidobacterium animalis lactis HN019 (DR10TM), inulin, and micronutrient fortified milk on faecal DR10TM, immune markers, and maternal micronutrients among Indonesian pregnant women. Asia pacific journal of clinical nutrition. 2016;25:S102‐S110.

3. Muthayya S, Thankachan P, Hirve S, Amalrajan V, Thomas T, Lubree H, Agarwal D, Srinivasan K, Hurrell RF, Yajnik CS, et al. Iron Fortification of Whole Wheat Flour Reduces Iron Deficiency and Iron Deficiency Anemia and Increases Body Iron Stores in Indian School-Aged Children. The Journal of Nutrition. 2012;142:1997–2003.

1. Abbreviations: MMN, multiple micronutrients; NA, Not Applicable

   **P*<0.05

   ***P* <0.01

   Sample size included in analysis [↑](#endnote-ref-1)
2. Population characteristics included are age and health status [↑](#endnote-ref-2)
3. Durations were converted to months using the following methodology: 4 weeks=1 month, 30 days=1 month, 1 year=12 [↑](#endnote-ref-3)
4. All participants received an intervention [↑](#endnote-ref-4)
