# Supplementary material for: Effects of Foods Fortified with Zinc, Alone or Cofortified with Multiple Micronutrients, on Health and Functional Outcomes: A Systematic Review and Meta-Analysis
Source: Adv Nutr. 2021 Jun 24;12(5):1821–37. doi: 10.1093/advances/nmab065 (PMC8483949; doi:10.1093/advances/nmab065)
Supplement: nmab065_Supplemental_Files [file nmab065_supplemental_files.zip › Supplemental Table 15. Descriptive zinc absorption.docx]

**Table S15: Fractional and total absorbed zinc of zinc-fortified foods – studies not included in meta-analysis due to lack of non-zinc fortified control food (n=7)^[[1]](#endnote-1),^^[[2]](#endnote-2)^**

| Reference | *n* | Population characteristics^[[3]](#endnote-3)^ | Fortified food^[[4]](#endnote-4)^, zinc compound | Study arms, dose (mg/d or mg/kg)^[[5]](#endnote-5)^ | FAZ, % (mean ± SD)^[[6]](#endnote-6)^ | | TAZ, mg (mean ± SD)^6^ | |
| --- | --- | --- | --- | --- | --- | --- | --- | --- |
|  |  |  |  |  | *Control/Baseline* | *Intervention/End line* | *Control/Baseline* | *Intervention/End line* |
| Herman et al. 2002 (1)  *Indonesia* | 86 | 4-8 y  Healthy | Wheat flour, dumplings | No zinc  ZnO: 60 mg/kg (1.5 mg/d)  ZnSO_4_: 60 mg/kg (1.5 mg/d)  All arms co-fortified with 60 mg Fe/kg | NM | Group 2: 24.1 ± 8.2^X^  Group 3: 23.7 ± 11.2^X^ | NM | Group 2: 0.361 ± 0.123  Group 3: 0.33 ± 0.167 |
| López de Romaña et al. 2003 (2)  *Peru* | 22 | 3-4 y | Wheat flour, bread or porridge | ZnO, 60 mg/kg (3 mg/d)  Bread or porridge  ZnSO_4_, 60 mg/kg (3 mg/d)  Bread or porridge  All arms co-fortified with 60 mg Fe/kg (3 mg/d) | NA | ZnO, bread  13.9 ± 5.0^7^  ZnO, porridge  5.6 ± 2.3^7^  ZnSO_4_, bread  13.8 ± 5.6^[[7]](#endnote-7)^  ZnSO_4_, porridge  7.5 ± 2.4^7^ |  | ZnO, bread  0.46 ± 0.16  ZnO, porridge  0.20 ± 0.07  ZnSO_4_, bread  0.56 ± 0.21  ZnSO_4_, porridge  0.24 ± 0.07 |
| Hotz et al. 2005 (3)  *Mexico* | 44 | 19-44 y  Healthy | Maize flour, tortilla | Group 1: ZnO  Group 2: ZnO + Na_2_EDTA  Group 3: Na_2_ZnEDTA  Group 4: ZnSO_4_  3.3mg/d | NA | Group 1: 10.8 ± 0.9^X^  Group 2: 12.7 ± 1.5^X^  Group 3: 12.7 ± 1.5^X^  Group 4: 10.0 ± 1.9^X^ | NA | NR |
| Mishaan et al. 2004 (4)  *Peru* | 39 | 6-9 y | Beverage | Fortified beverage + meal  Fortified beverage, no meal  3.75 mg Zn in the beverage | NA | Meal: 24.5 ± 10.7^X^  No meal: 22.8 ± 7.6^X^ | NA | Meal: 0.92  No meal: 0.86 |
| Hackl et al. 2017 (5)  *Switzerland* | 19 | 18-40 y | Rice | HER2: 6.9 ± 0.27 mg Zn/meal  COR: 7.1 ± 0.18 mg Zn/meal | NA | HER2: 9.54 (7.87, 11.57)^[[8]](#endnote-8)^  COR: 9.63 (8.70, 10.66)^8^ | NA | HER2: 0.49 (0.38, 0.60)^8^  COR: 0.59 (0.53, 0.66)^8^ |
| Hettiarachchi et al. (6)2004  *Sri Lanka* |  | 7-10 y | Rice flour | ZnO, 4.5 mg/d  ZnO + Na_2_EDTA, 4.5 mg/d  All arms co-fortified with Fe and FA | NA | ZnO: 8.8 ± 2.0^X^  ZnO + Na_2_EDTA: 13.5 ± 6^y^ | NA | ZnO: 0.202  ZnO + Na_2_EDTA: 0.132 |

**References:**

1. Herman S, Griffin IJ, Suwarti S, Ernawati F, Permaesih D, Pambudi D, Abrams SA. Cofortification of iron-fortified flour with zinc sulfate, but not zinc oxide, decreases iron absorption in Indonesian children. American Journal of Clinical Nutrition. 2002;76:813–7.

2. López de Romaña DL, Lonnerdal B, Brown KH. Absorption of zinc from wheat products fortified with iron and either zinc sulfate or zinc oxide. American Journal of Clinical Nutrition. 2003;78:279–83.

3. Hotz C, DeHaene J, Woodhouse LR, Villalpando S, Rivera JA, King JC. Zinc absorption from zinc oxide, zinc sulfate, zinc oxide + EDTA, or sodium-zinc EDTA does not differ when added as fortificants to maize tortillas. Journal of Nutrition. 2005;135:1102–5.

4. Mishaan AMA, Zavaleta N, Griffin IJ, Hilmers DC, Hawthorne KM, Abrams SA. Bioavailability of iron and zinc from a multiple micronutrient-fortified beverage. Journal of Pediatrics. 2004;145:26–31.

5. Hackl L, Speich C, Zeder C, Sanchez-Ferrer A, Adelmann H, de Pee S, Tay F, Zimmermann MB, Moretti D. Cold Extrusion but Not Coating Affects Iron Bioavailability from Fortified Rice in Young Women and Is Associated with Modifications in Starch Microstructure and Mineral Retention during Cooking. Journal of Nutrition. 2017;147:2319–25.

6. Hettiarachchi M, Hilmers DC, Liyanage C, Abrams SA. Na2EDTA Enhances the Absorption of Iron and Zinc from Fortified Rice Flour in Sri Lankan Children. The Journal of Nutrition. 2004;134:3031–6.

1. Papers organized by: Fortified food [↑](#endnote-ref-1)
2. Abbreviations (alphabetical): COR, cold extruded rice; HER2, hot extruded rice containing isotopically labeled ferric pyrophosphate, isotopically labeled zinc oxide, citric acid and trisodium citrate mixture, and micronutrients; mg/kg, milligrams of zinc per kilogram of fortified food; NM, not measured; NR, not reported; WRA, women of reproductive age. [↑](#endnote-ref-2)
3. Age as reported by authors, in range or mean/median [↑](#endnote-ref-3)
4. Staple food: product prepared with that staple food (in the case of maize and wheat flours), and fortification level (if reported). [↑](#endnote-ref-4)
5. All doses refer to extrinsically added zinc [↑](#endnote-ref-5)
6. Results converted to mean ± SD if reported otherwise. Different superscripts between interventions within the same row (a, b, c or x, y, z) indicate statistically significant differences (as reported by study authors). Results without superscripts indicate no statistical test conducted. [↑](#endnote-ref-6)
7. Geometric mean ± standard deviation [↑](#endnote-ref-7)
8. Geometric mean ± 95% CI [↑](#endnote-ref-8)
