# Supplementary material for: Effects of Foods Fortified with Zinc, Alone or Cofortified with Multiple Micronutrients, on Health and Functional Outcomes: A Systematic Review and Meta-Analysis
Source: Adv Nutr. 2021 Jun 24;12(5):1821–37. doi: 10.1093/advances/nmab065 (PMC8483949; doi:10.1093/advances/nmab065)
Supplement: nmab065_Supplemental_Files [file nmab065_supplemental_files.zip › Supplemental Table 6. PZC and zinc deficiency.docx]

**Table S6. Effect of foods fortified with zinc, alone or co-fortified with multiple micronutrients, on serum and plasma zinc outcomes**

| Reference  *Study location* | *n*^[[1]](#endnote-1)^ | | Population  characteristics^[[2]](#endnote-2)^ | Zinc fortified food | | Zinc dose/fortification level, duration^[[3]](#endnote-3),^^[[4]](#endnote-4)^ | Control group | | Baseline zinc status^[[5]](#endnote-5)^ | Serum/plasma zinc  (µg/dL)^[[6]](#endnote-6),^^[[7]](#endnote-7)^ | | Zinc deficiency  Prevalence (%) | | | |  |
| --- | --- | --- | --- | --- | --- | --- | --- | --- | --- | --- | --- | --- | --- | --- | --- | --- |
| *Efficacy Studies (n=27)* | |  | | | | | | | | | | | | | |  |
| *Liquid Foods (n=15)* | | | | | | | | | | | | | | | |  |
| Thankachan et al. 2013 (1)  *India* | 243 | | 6-12 y  Healthy | | Beverage | 1.2 mg/d, 2 mo | Non-fortified  beverage | | Not Deficient | Baseline  C: 130.9 ± 28.7  I: 129.7 ± 27.9  End line  C: 129.4 ± 26.8  I: 130.2 ± 28.5 | | Baseline for both groups: 0% | | | |  |
| Angeles-Agdeppa et al.  2011 (2)  *Philippines* | 89 | | 6-9 y  Anemic | | Beverage, non-carbonated,  orange flavored | 2.8 mg/d, 3.3 mo | Orange-flavored beverage fortified only with vitamin C | | Not Deficient | Baseline:  C: 96.2 ± 24.1  I: 83.9 ± 23.3  End line:  C: 96.6 ± 22.7  I: 104 ± 26.1* | | Baseline:  C: 4.7  I: 19.6  End line:  C: 7.0  I: 2.2 | | | |  |
| Abrams et al. 2003 (3)  *Botswana* | 263 | | 5-11 y  Healthy | | Beverage, fruit-flavored | 3.75 mg/d, 2 mo | Non-fortified fruit flavored beverage | | NA^[[8]](#endnote-8)^ | NR | | Baseline: NA  End line:  C: 14.5  I: 4.6* | | | |  |
| Angeles-Agdeppa et al.  2017 (4)  *Philippines* | 228 | | 6-9 y  Anemic | | Beverage, juice | *Low*:  5.6 mg/d for 3 d/wk  *Moderate*:  5.6 mg/d for 5 d/wk  *High:*  5.6 mg/d for 7 d/wk  4 mo | Non-fortified juice beverage | | Deficient | Baseline:  C: 64 (3)^[[9]](#endnote-9)^  Low: 63 (3)^9^  Mod: 64 (3)^9^  High: 64 (2)^9^  End line^11^:  C: 68 (2)^9^  Low: 66 (2)^9^  Mod: 73 (3)^9^  High: 67 (3)^9^ | | Baseline  C: 56.9  Low: 60.7  Mod: 55.4  High: 55.2  End line  C: 56.9  Low: 50  Mod: 39.3  High: 44.8 | | | |  |
| Hyder et al. 2007 (5)  *Bangladesh* | 1,125 | | I: 11.9 y  C: 12.0 y  Healthy girls | | Beverage, orange-flavored | 7.5 mg/d, 12 mo | Non-fortified orange-flavored beverage | | Deficient | Baseline  C: 65.23 ± 19.00  I: 64.16 ± 17.52  End line:  C: 85.40 ± 29.74  I: 88.65 ± 31.98* | | NR | | | |  |
| Aaron et al. 2011 (6)  *Nigeria* | 534 | | 5-13 y  Healthy | | Beverage, blend of pre-cooked maize and soy protein isolate | 15 mg/d, 6 mo | Non-fortified beverage | | Not Deficient | C: 3.92 ± 20.42^[[10]](#endnote-10)^  I: 6.54 ± 20.67^10^* | | Baseline:  C: 45.8  I: 43.2  End line:  C: 46.2  I: 36.3* | | | |  |
| Do et al. 2009 (7)  *Viet Nam* | 454 | | 7-8 y  Healthy | | Milk | 5.5 mg/d, 6 mo | Non-fortified milk | | Not Deficient | Baseline  C: 73.86 ± 12.42  I: 76.47 ± 13.73  End line  C: 79.74 ± 13.07  I: 82.35 ± 16.99 | | Baseline  C: 21  I: 35.3  End line  C: 20.7  I: 19.0* | | | |  |
| Bardosono et al. 2009 (8)  *Indonesia* | 245 | | 7-9 y  Healthy | | Milk powder | 2.38 mg/d, 6 mo | Non-fortified milk | | Not Deficient | Baseline  C: 87.58 ± 21.57  I: 88.76 ± 20.13  End line  C: 67.9 ± 24.31  I: 69.08 ± 21.18 | | C: -30.6^10^  I: -24.8^10^ | | | |  |
| Costarelli et al. 2014 (9)  *Italy* | 21 | | ≥ 82 y  Healthy | | Milk, skim | 4 mg/d, 2 mo | Non-fortified milk  (cross over study) | | NR | End line  C: 89.28 ± 33.85  I: 101.24 ± 40.73 | | NR | | | |  |
| Villalpando et al. 2006 (10)  *Mexico* | 115 | | 10-30 mo  Healthy | | Milk powder | 5.28 mg/d, 6 mo | Non-fortified milk | | Not Deficient | End line  C: 85.42 ± 6.54  I: 82.42 ± 6.27 | | NR | | | |  |
| Méndez et al. 2012 (11)  *Mexico* | 131 | | 12-17 y  Healthy girls | | Milk powder | 6.6 mg/d, 6 mo | Pre-intervention usual diet (which included non-fortified milk) | | Not Deficient | Baseline:  C: 113 ± 35.3  I: 108 ± 30.3  End line:  C: 98.5 ± 26.6  I: 116 ± 26.9 | | NR | | | |  |
| Sazawal et al. 2010 (12)  *India* | 524 | | 1-3 y  NR | | Milk powder | 9.6 mg/d, 24 mo | Non-fortified milk | | Deficient | Baseline:  C: 62.60 ± 26.80  I: 60.70 ± 23.30  End line:  C: 63.40 ± 29.40  I: 61.40 ± 26.80 | | NR | | | |  |
| Wibowo et al. 2016 (13)  *Indonesia* | 104 | | 18-35 y  Pregnant women | | Milk powder | 5.25 mg/d, 9 mo | Milk powder fortified  with folic-acid and  iron | | Deficient | 1^st^ trimester  C: 65.75 ± 13.70  I: 63.2 ± 13.9  3^rd^ trimester  C: 48.8 ± 8.6  I: 51.0 ± 8.14* | | NR | | | |  |
| Trinidad et al. 2015 (14)  *Philippines* | 124 | | ≥6 y  Healthy | | Milk powder | NR, 4 mo  Group 1: 1 glass milk/d  Group 2: 2 glasses milk/d | Group 3: water | | Not Deficient | Baseline  Group 1: 66.4 ± 3.3  Group 2: 82.3 ± 4.3  Group 3: 91.1 ± 4.1  End line  Group 1: 99.1 ± 4.1*  Group 2: 87.5 ± 4.7*  Group 3: 85.6 ± 5.6* | | NR | | | |  |
| Sazawal et al. 2013 (15)  *India* | 524 | | 6-9 y  NR | | Yogurt | 3 mg/d, 12 mo | Non-fortified yogurt | | Deficient | Baseline  C: 60.26 ± 23.86  I: 59.54 ± 21.57  End line  C: 68.37 ± 26.21  I: 68.95 ± 28.56 | | NR | | | |  |
| *Cereal grains or Condiments (n=12)* | | | | | | | | | | | | | | | |  |
| López de Romaña et al. 2005 (16)  *Peru* | 31 | | 3-4 y  Anemic children  at high risk of  zinc deficiency | Wheat products: rice, chicken, milk, wheat bread, potato, oatmeal, wheat noodles, and beans | | 30 mg/kg flour:  4.72 mg/d  90 mg/kg flour:  10.04 mg/d  2.3 mo | Non-fortified flour | Not Deficient | | Non-fortified flour:  Baseline: 70.8 ± 8.8  End line: 77.6 ± 12.3  30 mg/kg flour  Baseline: 77.4 ± 13.1  End line: 76.2 ± 8.2  90 mg/kg flour  Baseline: 77.6 ± 16.2  End line: 82.4 ± 9.9 | | | Non-fortified flour:  Baseline: 23.1  End line: 10.0  30 mg/kg flour  Baseline: 18.2  End line: 0  90 mg/kg flour  Baseline: 20  End line: 0 | | |  |
| Nga 2009 et al. (17)  *Viet Nam* | 466 | | 6-8 y  Children with high  prevalence of anemia  and parasite  infestations | Wheat flour, biscuits  Co-intervention:  deworming (400mg  Albendazole) or placebo  pill | | 5.6 mg/d, 4 mo | Non-fortified biscuits | Deficient | | Placebo biscuits + placebo pill  Baseline: 57.52 ± 14.38  End line: 57.52 ± 12.42  Fortified biscuits + placebo pill  Baseline: 55.56 ± 13.07  End line: 61.44 ± 12.42*  Placebo biscuits + deworming  Baseline: 56.21 ± 14.38  End line: 56.86 ± 12.42  Fortified biscuits + deworming  Baseline: 55.56 ± 14.38  End line: 61.44 ± 12.42* | | | Placebo biscuits + placebo pill  Baseline: 52.5  End line: 53.4  Fortified biscuits + placebo pill  Baseline: 59.6  End line: 36**  Placebo biscuits + deworming  Baseline: 52.1  End line: 53  Fortified biscuits + deworming:  Baseline: 59  End line: 39.3* | | |  |
| Hieu et al. 2012 (18)  *Viet Nam* | 290 | | 6-9 y  NR | Wheat flour, biscuits | | 3.5 mg/d, 6 mo | Non-fortified biscuit | Not Deficient | | Baseline  C: 69.93 ± 20.92  I: 66.67 ± 13.73  End line:  C: 70.59 ± 16.99  I: 71.24 ± 16.99 | | | NR | | |  |
| Aaron et al, 2011b (19)  *Senegal* | 130 | | >18 y | Wheat flour, bread  Co-intervention: liquid MNN supp., no zinc | | 7.5 mg/d or 15 mg/d, 1 mo | Bread fortified only with iron and folic acid | Deficient | | 7.5 mg/d: 1.96 ± 8.64^10^  15 mg/d: 2.09 ± 8.73^10^  C: 3.07 ± 8.64^10^ | | | 7.5 mg/d: 80.6  15 mg/d: 65.7  C: 64.1  End line:  7.5 mg/d: 75.8  15 mg/d: 67.7  C: 48.5 | | |  |
| Kiliç et al. 1998 (20)  Saldamli et al. 1996 (21)  *Turkey* | 24 | | 7-11 y | Wheat flour, bread | | 54.4 mg/d, 3 mo | Non-fortified  bread | Deficient | | Baseline  C: 58.9 ± 2.7  I: 60.8 ± 3.6  End line  C: 62.9 ± 3.4*  I: 81.5 ± 9.1* | | | NR | | |  |
| Badii et al. 2011 (22)  *Iran* | 75 | | 32.2 y  NPNL women | Wheat flour: Iranian flatbread  (Taftoon) | | 9.15 mg/d or 16.16 mg/d  1 mo | Non-fortified bread | Deficient | | 9.15 mg/d  Baseline: 65.06 ± 0.95  End line: 86.8 ± 10.78**  16.16 mg/d  Baseline: 66.53 ± 3.17  End line: 78.73 ± 8.1**  Non-fortified  Baseline: 65.53 ± 3.38  End line: 67.73 ± 2.17 | | | | NR | | |
| Haibin et al. 2001 (23) | 67 | | 24.7 ± 2.4^[[11]](#endnote-11)^  Pregnant women | Flour (unspecified), biscuits | | 10 mg/d, 4-5 mo  Ca+Zn+VD or  Ca+Fe+Zn+VD | Fortified biscuits without zinc:  Ca+VD or  Ca+Fe+VD | Not Deficient | | Baseline:  Ca+VD: 88.89 ± 90.20  Ca+Zn+VD: 89.54 ± 61.44  Ca+Fe+VD: 84.97 ± 92.16  Ca+Fe+Zn+VD: 70.59 ± 58.17  End line:  Ca+VD: 74.51 ± 76.47*  Ca+Zn+VD: 109.80 ± 84.97  Ca+Fe+VD: 82.35 ± 41.83  Ca+Fe+Zn+VD: 100.00 ± 55.56* | | | | NR | | |
| Hettiarachchi et al. 2004 (24)  *Sri Lanka* | 49 | | 7-10 y | Rice, flour | | 4.5 mg/d, 1 mo  Fe+Zn+FA or  Fe+Zn+FA+Na_2_EDTA | Fortified rice flour without zinc:  Fe+FA or  Fe+FA+Na_2_EDTA | Not deficient | | Baseline:  Fe+FA: 79.74 ± 17.65  Fe+Zn+FA: 77.12 ± 15.03  Fe+FA+Na_2_EDTA: 77.12 ± 15.69  Fe+Zn+FA+Na_2_EDTA: 79.08 ± 11.11  End line:  Fe+FA: 80.39 ± 18.95  Fe+Zn+FA: 84.97 ± 16.34  Fe+FA+Na_2_EDTA: 79.08-14.38  Fe+Zn+FA+Na_2_EDTA: 83.01 ± 16.99 | | | | NR | | |
| Ibnu et al. 2019 (25)  *Indonesia* | 124 | | 12-15 y  Healthy males | Rice | | NR, 6 mo | Non-fortified rice | Not Deficient | | *Anemic group*  Baseline  C: 89.10 ± 9.16  I: 76.25 ± 11.37  End line  C: 59.06 ± 7.81  I: 54.46 ± 6.76*  *Non-anemic group*  Baseline  C: 84.80 ± 7.97  I: 88.50 ± 13.79  End line  C: 60.47 ± 9.43  I: 59.33 ± 8.41 | NR | | | |  |  |
| Pinkaew et al. 2013  *Thailand* | 182 | | 4-12 y  Zinc deficient | Rice, hot extrusion | | 9 mg/d, 5 mo | Non-fortified rice | Deficient | | Baseline  C: 61.44 ± 3.92  I: 61.44 ± 3.92  End line:  C: 69.28 ± 9.15*  I: 72.55 ± 8.50* | | Baseline  C: 100  I: 100  End line  C: 39*  I: 29* | | | | |
| Winichagoon et al. 2006 (26)  *Thailand* | 555 | | 5.5-13.4 y  Healthy | Seasoning Powder | | 5 mg/d, 7.75 mo | Non-fortified seasoning powder | NR | | End line  C: 71.24 ± 10.92  I: 73.20 ± 10.65* | | Baseline  C: 54.7  I: 53.4  End line  C: 34.7  I: 27.5* | | | | |
| Vinodkumar et al. 2009 (27)  *India* | 371 | | I: 12.21 y  C: 12.27 y | Salt | | 10 mg/d, 9 mo | Iodized salt | Not Deficient | | Baseline  C: 101.22 ± 48.3  I: 92.5 ± 39.6  End line  C: 102.73 ± 88.78  I: 142.5 ± 132* | | NR | | | | |
| *Controlled Effectiveness Studies (n=7)* | | | | | | | | | | | | | | | |  |
| Della Lucia et al. 2016 (28)  *Brazil* | 99 | | 2-6 y  Healthy | Rice, extrusion  (UltraRice) | | 2.1 mg/d, 4 mo | Non-fortified rice | | Not Deficient | Baseline:  C: 95.86 ± 21.90  I: 80.97 ± 11.88  End line:  C: 92.13 ± 15.92  I: 110.21 ± 22.76* | | Baseline:  0% in both groups | | | |  |
| Kuong et al. 2019 (29)  *Cambodia* | 1796 | | 6-16 y  Healthy | Rice, cold & hot extrusion | | UltraRice Original (URO):  3.5 mg/d  UltraRice New (URN):  2.3 mg/d  NutriRice (NR):  4.2 mg/d  7 mo | Non-fortified rice | | Deficient | Baseline:  URO: 51.18 ± 11.13  URN: 50.13 ± 11.31  NR: 53.27 ± 11.40  C: 51.18 ± 11.13  End line:  URO: 55.95 ± 12.03*  URN: 54.05 ± 12.17*  NR: 60.72 ± 12.06*  C: 49.48 ± 11.78* | | Baseline:  URO: 90.1  URN: 92.3  NR: 83.6  C: 89.4  End line:^[[12]](#endnote-12)^  URO: 80.6  URN: 83.4  NR: 65.7  C: 93.3 | | | |  |
| Ara et al. 2019 (30)  *Bangladesh* | 800 | | 15-49 y  Healthy | Rice, extrusion | | 40 mg/kg, 12 mo | Non-fortified rice | | Not Deficient | Pre:  C: 69.28  I: 70.59  Post:  C: 69.93  I:70.59 | | Pre:  C: 31.2  I: 37.2  Post:  C: 37.2  I: 37.6 | | | |  |
| Dutta et al. 2019 (31)  *India* | 1,764 (pre)  1,638 (post) | | 6-14 y | Rice, extrusion | | Rice: 15 mo | Usual mid-day meal | | Not Deficient | NR | | Pre:  I: 24.4  C: NR  Post:  I: 19.0  C: 29.4 | | | |  |
| Huo et al. 2011 (32)  *China* | 611 | | 20-60 y  Healthy | Wheat flour | | 2.18 mg/d, 36 mo | Non-fortified wheat  flour | | Not Deficient | Baseline:  C: 73 ± 25  I: 75 ± 27  End line:  C: 71 ± 19  I: 79 ± 16* | | NR | | | |  |
| Huo et al. 2012 (33)  *China* | 448 | | 20-60 y  Healthy, nonpregnant  women | Wheat flour | | 4.88 mg/d, 36 mo | Non-fortified wheat  flour | | Not Deficient | Baseline  C: 73 ± 17  I: 72 ± 19  End line  C: 75 ± 13  I: 78 ± 11* | | NR | | | |  |
| Ohiokpehai, et al. 2009 (34)  *Kenya* | 135 | | 6-9 y | Maize-soy porridge | | 5 mg/d, 3 mo  *co-intervention: vitamin A supplementation at baseline | No food | | Deficient | Baseline  C: 64.05 ± 7.19^[[13]](#endnote-13)^  I: 48.37 ± 7.84(28),^[[14]](#endnote-14)^  End line  C: 67.32 ± 7.19^14^  I: 71.50 ± 8.50^14,15^* | | Baseline  C: 94.3%  I: 96.0%  End line  C: 61.4%*  I: 74.4%* | | | |  |
| Hambidge et al. 1979 (35)  *United States* | 93 | | 33-90 mo  Healthy | Cereal, ready to eat | | 2.57 mg/d, 9 mo | Non-zinc fortified  cereal | | Not Deficient | C: -8.7 ± 3^10^  I: -2.3 ± 2.3^10^ | | NR | | | |  |
| *Non-Controlled Effectiveness Studies (n=7)* | | | | | | | | | | | | | | | |  |
| Varea et al. 2011 (36)  *Argentina* | 474 | | 1-6 y | Maize + soy flour blend, children 1-2 y received additional complementary foods: precooked wheat cereals and pudding | | 1-2 y: 0.7 mg/d  2-6 y: 1.5 mg/d  12 mo | NA | | Not Deficient | 1-2 y  Pre: 88.7 (85.1-92.4)^[[15]](#endnote-15)^  Post: 94 (89.7-98.5)^15^*  2-6 y  Pre: 85.9 (83.5-88.8)^15^  Post: 95.9 (93.6-98.2)^15^* | | 1-2 y  Pre: 7.5  Post: 11.8  2-6 y  Pre: 7.3  Post: 5 | | | |  |
| Varea et al. 2012 (37)  *Argentina* | 474 | | 15-47 y  Healthy lactating  women | Maize + soy flour blend | | 1.41 mg/d, 12 mo | NA | | Not Deficient | Pre: 84.8 (82.1-87.6)^15^  Post: 85.5 (82.8-88.3)^15^* | | Pre: 8.2  Post: 10.2 | | | |  |
| Stuetz et al. 2012 (38)  *Thailand*  *(Maela refugee camp)* | Pre: 86  Post: 98 | | Pre: 16-41 y  Post: 17-46 y  Breastfeeding women | Wheat flour | | 2.7 mg/d, 3-4 mo | NA | | Deficient | Pre: 61.50 ± 12.0  Post: 66.30 ± 15.40* | | Pre: 63  Post: 49* | | | |  |
| Engle-Stone et al. 2017 (39)  *Cameroon* | Pre:  Women: 279  Children: 272  Post:  Women: 302  Children: 303 | | Women  15-49 y  Healthy  Children  12-59 mo | Wheat flour | | 73.6 ± 43.0 mg/kg | NA | | Not Deficient | Women  Pre: 55.1 ± 0.6^16^  Post: 65.2 ± 1.5^16^*  Children  Pre: 56.6 ± 0.9^16^  Post: 66.7 ± 1.6^16^* | | Women  Pre: 39.4^[[16]](#endnote-16)^  Post: 21.6^16^*  Children  Pre: 46.8^16^  Post: 28.4^16^* | | | |  |
| Sun et al. 2013 (40)  *China* | 192 | | 11-16 y | Rice | | 5.75 mg/d, 10 mo | NA | | Not Deficient | Baseline: 78 ± 25  End line: 92 ± 23 | | Baseline: 35.3  End line: 13.3* | | | |  |
| Méndez et al. 2014 (41)  *Mexico* | 24 | | 12-16 y  Healthy girls | Milk powder | | 6.6 mg/d, 0.9 mo | NA | | Not Deficient | Baseline: 142.4 ± 20.9  End line: 146.2 ± 25.7 | | NR | | | |  |
| Tukvadze & Kverenchkhiladze 2013 (42)  *Georgia* | 36 | | 11-14 y  Healthy | Tea | | NR, 3 mo | NA | | NR | NR | | Baseline:  Boys: 85.7  Girls: 86.3  End line:  Boys: 28.6  Girls: 22.7* | | | |  |

**References**

1. Thankachan P, Rah JH, Thomas T, Selvam S, Amalrajan V, Srinivasan K, et al. Multiple Micronutrient-Fortified Rice Affects Physical Performance and Plasma Vitamin B-12 and Homocysteine Concentrations of Indian School Children. The Journal of Nutrition. 2012 May 1;142(5):846–52.

2. Angeles-Agdeppa I, Magsadia CR, Capanzana MV. Fortified juice drink improved iron and zinc status of schoolchildren. Asia Pacific Journal of Clinical Nutrition. 2011;20(4):535–43.

3. Abrams SA, Mushi A, Hilmers DC, Griffin IJ, Davila P, Allen L. A multinutrient-fortified beverage enhances the nutritional status of children in Botswana. Journal of Nutrition. 2003;133(6):1834–40.

4. Angeles-Agdeppa I, Magsadia CR, Aaron GJ, Lloyd BB, Hilmers DC, Bhutta ZA. A Micronutrient Fortified Beverage Given at Different Dosing Frequencies Had Limited Impact on Anemia and Micronutrient Status in Filipino Schoolchildren. Nutrients [Internet]. 2017;9(9). Available from: ://WOS:000411973200085

5. Ziauddin Hyder SM, L©œnnerdal B, Rahman M, Mehansho H, Mannar V, Khan M, et al. A Multiple-Micronutrient-Fortified Beverage Affects Hemoglobin, Iron, and Vitamin A Status and Growth in Adolescent Girls in Rural Bangladesh. Journal of Nutrition. 2007;137(9):2147–53.

6. Aaron GJ, Kariger P, Aliyu R, Flach M, Iya D, Obadiah M, et al. A Multi-Micronutrient Beverage Enhances the Vitamin A and Zinc Status of Nigerian Primary Schoolchildren. Journal of Nutrition. 2011;141(8):1565–72.

7. Do TKL, Bui TN, Nguyen CK, Le TH, Nguyen TQN, Nguyen TH, et al. Impact of milk consumption on performance and health of primary school children in rural Vietnam. Asia Pacific Journal of Clinical Nutrition. 2009;18(3):326–34.

8. Bardosono S, Dewi LE, Sukmaniah S, Permadhi I, Eka AD, Lestarina L. Effect of a six-month iron-zinc fortified milk supplementation on nutritional status, physical capacity and speed learning process in Indonesian underweight schoolchildren: Randomized, placebo-controlled. Medical Journal of Indonesia. 2009;18(3):193–202.

9. Costarelli L, Giacconi R, Malavolta M, Basso A, Piacenza F, DeMartiis M, et al. Effects of zinc-fortified drinking skim milk (as functional food) on cytokine release and thymic hormone activity in very old persons: A pilot study. Age. 2014;36(3):1421–31.

10. Villalpando S, Shamah T, Rivera JA, Lara Y, Monterrubio E. Fortifying milk with ferrous gluconate and zinc oxide in a public nutrition program reduced the prevalence of anemia in toddlers. Journal of Nutrition. 2006;136(10):2633–7.

11. Méndez RO, Galdamez K, Grijalva MI, Quihui L, Garcia HS, de la Barca AM. Effect of micronutrient-fortified milk on zinc intake and plasma concentration in adolescent girls. J Am Coll Nutr. 2012;31(6):408–14.

12. Sazawal S, Dhingra U, Dhingra P, Hiremath G, Sarkar A, Dutta A, et al. Micronutrient fortified milk improves iron status, anemia and growth among children 1-4 years: A double masked, randomized, controlled trial. PLoS ONE [Internet]. 2010;5(8). Available from: https://www.scopus.com/inward/record.uri?eid=2-s2.0-77957861492&doi=10.1371%2fjournal.pone.0012167&partnerID=40&md5=7c3940d6762d5c82fbccb481dc8cce42

13. Wibowo N, Bardosono S, Irwinda R. Effects of Bifidobacterium animalis lactis HN019 (DR10TM), inulin, and micronutrient fortified milk on faecal DR10TM, immune markers, and maternal micronutrients among Indonesian pregnant women. Asia Pacific Journal of Clinical Nutrition. 2016;25:S102–10.

14. Trinidad TP, Mallillin AC, Sagum RS, de Leon MP, Borlagdan MS, Baquiran AFP. Fortified milk consumption among 6-year old children: changes in biochemical markers of trace minerals and vitamins. Trace Elements and Electrolytes. 2015;32(3):112–8.

15. Sazawal S, Habib A, Dhingra U, Dutta A, Dhingra P, Sarkar A, et al. Impact of micronutrient fortification of yoghurt on micronutrient status markers and growth - a randomized double blind controlled trial among school children in Bangladesh. BMC Public Health. 2013;13:514.

16. López de Romaña D, Peerson JM, Krebs NF, Brown KH, Salazar M, Hambidge KM, et al. Longitudinal measurements of zinc absorption in Peruvian children consuming wheat products fortified with iron only or iron and 1 of 2 amounts of zinc. American Journal of Clinical Nutrition. 2005;81(3):637–47.

17. Nga TT, Wasantwisut E, Furr H, Wieringa FT, Winichagoon P, Dijkhuizen MA, et al. Multi-Micronutrient-Fortified Biscuits Decreased Prevalence of Anemia and Improved Micronutrient Status and Effectiveness of Deworming in Rural Vietnamese School Children. Journal of Nutrition. 2009;139(5):1013–21.

18. Hieu NT, Sandalinas F, De Sesmaisons A, Laillou A, Tam NP, Khan NC, et al. Multi-micronutrient-fortified biscuits decreased the prevalence of anaemia and improved iron status, whereas weekly iron supplementation only improved iron status in Vietnamese school children. British Journal of Nutrition. 2012;108(8):1419–27.

19. Aaron GJ, Ba Lo N, Hess SY, Guiro AT, Wade S, Brown KH. Plasma Zinc Concentration Increases within 2 Weeks in Healthy Senegalese Men Given Liquid Supplemental Zinc, but Not Zinc-Fortified Wheat Bread. The Journal of Nutrition. 2011 Jul 1;141(7):1369–74.

20. Kiliç I, Ozalp I, Coskun T, Tokatli A, Emre S, Saldamli I, et al. The effect of zinc-supplemented bread consumption on school children with asymptomatic zinc deficiency. J Pediatr Gastroenterol Nutr. 1998;26(2):167–71.

21. Saldamli I, Ozalp I, Kilic I, Koksel H, Ozboy O. Zinc-supplemented bread and its utilization in zinc deficiency. Cereal Chemistry. 1996;73(4):424–7.

22. Badii A, Nekouei N, Fazilati M, Shahedi M, Badiei S. Effect of Consuming Zinc-fortified Bread on Serum Zinc and Iron Status of Zinc-deficient Women: A Double Blind, Randomized Clinical Trial. Int J Prev Med. 2012 Mar;3(Suppl 1):S124-130.

23. Haibin A, Yin S, Xu Q. [Effects of supplementing calcium, iron and zinc on the fetus development and growth during pregnancy]. Zhonghua Yu Fang Yi Xue Za Zhi. 2001 Nov;35(6):370–3.

24. Hettiarachchi M, Hilmers DC, Liyanage C, Abrams SA. Na2EDTA Enhances the Absorption of Iron and Zinc from Fortified Rice Flour in Sri Lankan Children. The Journal of Nutrition. 2004 Nov 1;134(11):3031–6.

25. Ibnu IN, Thaha RM, Suriah. Effect of iron and zinc substance giving through fortification rice on stress level of school age children in islamic boarding school annihayahkarawang. Indian Journal of Public Health Research and Development. 2019;10(4):1071–7.

26. Winichagoon P, Bailey KB, Manger MS, Gibson RS, Wasantwisut E, Boonpraderm A, et al. A Multimicronutrient-Fortified Seasoning Powder Enhances the Hemoglobin, Zinc, and Iodine Status of Primary School Children in North East Thailand: A Randomized Controlled Trial of Efficacy. Journal of Nutrition. 2006;136(6):1617–23.

27. Vinodkumar M, Erhardt JG, Rajagopalan S. Impact of a multiple-micronutrient fortified salt on the nutritional status and memory of schoolchildren. International Journal for Vitamin and Nutrition Research. 2009;79(5–6):348–61.

28. Della Lucia CM, Rodrigues KC, Rodrigues VC, Santos LL, Cardoso LM, Martino HS, et al. Diet Quality and Adequacy of Nutrients in Preschool Children: Should Rice Fortified with Micronutrients Be Included in School Meals? Nutrients. 2016;8(5).

29. Kuong K, Tor P, Perignon M, Fiorentino M, Chamnan C, Berger J, et al. Multi-Micronutrient Fortified Rice Improved Serum Zinc and Folate Concentrations of Cambodian School Children. A Double-Blinded Cluster-Randomized Controlled Trial. Nutrients. 2019 Nov 20;11(12).

30. Ara G, Khanam M, Rahman AS, Islam Z, Farhad S, Sanin KI, et al. Effectiveness of micronutrient-fortified rice consumption on anaemia and zinc status among vulnerable women in Bangladesh. PLoS ONE [Internet]. 2019;14(1). Available from: https://www.scopus.com/inward/record.uri?eid=2-s2.0-85059829140&doi=10.1371%2fjournal.pone.0210501&partnerID=40&md5=f0a7361196c7c59ee37c587f6f6665fc

31. Dutta A. Assessment of fortification of Mid-Day Meal Programme in Dhenkanal, Odisha, 2016-2018 Evaluation Report - India [Internet]. World Food Programme; 2020 [cited 2020 Jul 7]. Available from: https://reliefweb.int/report/india/assessment-fortification-mid-day-meal-programme-dhenkanal-odisha-2016-2018-evaluation

32. Huo J, Sun J, Huang J, Li W, Wang L, Selenje L, et al. The effectiveness of fortified flour on micro-nutrient status in rural female adults in China. Asia Pacific Journal of Clinical Nutrition. 2011;20(1):118–24.

33. Huo J, Gary RG, Jian H, Jing S, Lijuan W, Lilian S, et al. Effectiveness of Fortified Flour for Enhancement of Vitamin and Mineral Intakes and Nutrition Status in Northwest Chinese Villages. Food and Nutrition Bulletin. 2012;33(2):161–8.

34. Ohiokpehai O, David DM, Kamau J. Serum zinc levels of school children on a corn-soy blend feeding trial in primary schools in Suba district, Kenya. Journal of Applied Biosciences. 2009 May 8;17:904–12.

35. Hambidge KM, Chavez MN, Brown RM, Walravens PA. ZINC NUTRITIONAL-STATUS OF YOUNG MIDDLE-INCOME CHILDREN AND EFFECTS OF CONSUMING ZINC-FORTIFIED BREAKFAST CEREALS. American Journal of Clinical Nutrition. 1979;32(12):2532–9.

36. Varea A, Malpeli A, Etchegoyen G, Vojkovic M, Disalvo L, Apezteguia M, et al. Short-Term Evaluation of the Impact of a Food Program on the Micronutrient Nutritional Status of Argentinean Children Under the Age of Six. Biological Trace Element Research. 2011;143(3):1337–48.

37. Varea A, Carmuega E, Pereyras S, Etchegoyen G, Gonz©Łlez HF, Vojkovic M, et al. Evaluation of the Impact of a Food Program on the Micronutrient Nutritional Status of Argentinean Lactating Mothers [electronic resource]. Biological Trace Element Research. 2012;150(1–3):103–8.

38. Stuetz W, Carrara VI, McGready R, Lee SJ, Erhardt JG, Breuer J, et al. Micronutrient status in lactating mothers before and after introduction of fortified flour: Cross-sectional surveys in Maela refugee camp. European Journal of Nutrition. 2012;51(4):425–34.

39. Engle-Stone R, Nankap M, Ndjebayi AO, Allen LH, Shahab-Ferdows S, Hampel D, et al. Iron, Zinc, Folate, and Vitamin B-12 Status Increased among Women and Children in Yaounde and Douala, Cameroon, 1 Year after Introducing Fortified Wheat Flour. Journal of Nutrition. 2017;147(7):1426–36.

40. Sun J, Huang J, Huo JS. School food fortification improves nutrition status of students from poor migrant families. Annals of Nutrition and Metabolism. 2013;63:483.

41. Méndez RO, Santiago A, Yepiz-Plascencia G, Peregrino-Uriarte AB, Calderón de la Barca AM, García HS. Zinc fortification decreases ZIP1 gene expression of some adolescent females with appropriate plasma zinc levels. Nutrients. 2014;6(6):2229–39.

42. Tukvadze S, Kverenchkhiladze R. Inclusion of zinc fortified tea into the children’s diet and its hygienicassessment. Georgian medical news. 2013;(217):53–6.

1. *Significantly different from baseline or pre-fortification value, p<0.05

   Abbreviations: C, control group; I, Intervention group; NPNL, non-pregnant, non-lactating; NA, Not available; NR, not reported;

   Sample size included in analysis [↑](#endnote-ref-1)
2. Population characteristics included are age and health status, as reported by authors. Age is a range, unless footnoted otherwise. [↑](#endnote-ref-2)
3. If unit is expressed as mg/kg, the value is referring to fortification level. If unit is expressed as mg/day, the value is referring to dose. In effectiveness studies, where the fortified food was provided to households and fortification level was known, dose was calculated as follows: (X mg of zinc/1000 grams*intake in grams) [↑](#endnote-ref-3)
4. Durations were converted to months using the following methodology: 4 weeks=1 month, 30 days=1 month, 1 year=12 months [↑](#endnote-ref-4)
5. Study classified as deficient if >50% of population was zinc deficient (as defined by authors), or mean/median serum/plasma zinc concentration was below deficiency cut-offs by relevant age group according to IZiNCG reference values. [↑](#endnote-ref-5)
6. All serum/plasma zinc concentration values converted to µg/dL [↑](#endnote-ref-6)
7. Values are mean ± SD unless footnoted otherwise; Baseline/end line used to refer to studies where the same individuals were followed-up; Pre/post used to refer to studies where the pre and post measurements occurred in different individuals sampled from the same population. [↑](#endnote-ref-7)
8. The value was thrown out due to measurement error [↑](#endnote-ref-8)
9. Mean, standard error [↑](#endnote-ref-9)
10. Mean difference [↑](#endnote-ref-10)
11. Mean ± standard deviation [↑](#endnote-ref-11)
12. OR was significant compared to placebo [↑](#endnote-ref-12)
13. Authors labelled the error as 95% CI but only provided a single number. Here (and analysis), we assume that this was actually the standard deviation. [↑](#endnote-ref-13)
14. Values are for the Mbita and Sindo intervention groups combined. [↑](#endnote-ref-14)
15. Geometric mean (95% CI) [↑](#endnote-ref-15)
16. Adjusted for inflammation [↑](#endnote-ref-16)
