# Supplementary material for: Effects of Foods Fortified with Zinc, Alone or Cofortified with Multiple Micronutrients, on Health and Functional Outcomes: A Systematic Review and Meta-Analysis
Source: Adv Nutr. 2021 Jun 24;12(5):1821–37. doi: 10.1093/advances/nmab065 (PMC8483949; doi:10.1093/advances/nmab065)
Supplement: nmab065_Supplemental_Files [file nmab065_supplemental_files.zip › Supplemental Table 7. Anthropometry.docx]

Table S7. Effect of foods fortified with zinc, alone or co-fortified with multiple micronutrients, on child anthropometric outcomes^[[1]](#endnote-1)^

| Reference  *Study location* | | *n*^[[2]](#endnote-2)^ | Population  characteristics^[[3]](#endnote-3)^ | Fortified food, zinc dose/fortification level,  duration^[[4]](#endnote-4),^^[[5]](#endnote-5)^ | Weight (kg)^[[6]](#endnote-6)^ | | Height (cm)^6^ | WHZ^6^ | Wasting  (%) | HAZ^6^ | Stunting  (%) | WAZ^6^ | Under-  weight (%) | MUAC^6^  (cm) | |  |
| --- | --- | --- | --- | --- | --- | --- | --- | --- | --- | --- | --- | --- | --- | --- | --- | --- |
| *Efficacy studies (n=16)^[[7]](#endnote-7)^* | |  |  |  |  | |  |  |  |  |  |  |  |  | |  |
| *Liquid foods (n=9)* | |  |  |  |  | |  |  |  |  |  |  |  |  | |  |
| Abrams et al. 2003 (1)  *Botswana* | | 263 | 5-11 y  Healthy | Beverage, fruit-flavored ǂ  3.75 mg/d, 2 mo | C: 0.77 ± 0.84^11^  I: 1.24 ± 0.85^[[8]](#endnote-8)^ | | NR | NR | NR | NR | NR | C: 0.08 ± 0.23^11^  I: 0.17 ±0.27*^11^ | Baseline:  C: 7.2  I: 7.85  End line:  C: 5.6  I: 6.4 | C: 1.37 ± 9.3^11^  I: 2.15 ± 11.5*^11^ | |  |
| Angeles-Agdeppa et al. 2011 (2)  *Philippines* | | 89 | 6-9 y  Anemic | Beverage, non-carbonated orange-flavored juice ǂ  2.8 mg/d, 3.3 mo | Baseline:  C: 21.7 ± 3.3  I: 21.7 ± 3.2  End line:  C: 23.3 ± 3.5**  I: 23.4 ± 3.6** | | Baseline:  C: 120 ± 5.8  I: 120 ± 7.4  End line:  C: 123 ± 5.9**  I: 123 ± 7.4** | NR | NR | Baseline:  C: -1.21 ± 0.60  I: -1.15 ± 0.86  End line:  C: -1.08 ± 0.61**  I: -1.02 ± 0.86** | NR | Baseline:  C: -1.09 ± 0.73  I: -1.01 ± 0.66  End line:  C: -0.96 ± 0.73**  I: -0.88 ± 0.69** | NR | NR | |  |
| Rameshwar  Sarma et al.  2006 (3)  *India* | | 695 | School grades: 1-4^[[9]](#endnote-9)^ | Beverage, micronutrient- enriched health drink  4.6 mg/d, 12-14 mo | Grade 1  Baseline  C: 19.8^[[10]](#endnote-10)^  I: 19.4^10^  End line  C: 21.9^10^  I: 22.1^10^  Grade 2  Baseline  C:22.2^10^  I: 20.6^10^  End line  C: 25.1^10^  I: 23.7^10^  Grade 3  Baseline  C:23.8^10^  I: 22.0^10^  End line  C: 27.0^10^  I: 25.8^10^  Grade 4  Baseline  C:25.3^10^  I: 25.1^10^  End line  C: 28.3^10^  I: 29.0^10^ | | Grade 1  Baseline  C: 116.9^10^  I: 116.7^10^  End line  C: 123.3^10^  I: 125.1^10^  Grade 2  Baseline  C: 122.8^10^  I: 120.4^10^  End line  C: 129.4^10^  I: 128.4^10^  Grade 3  Baseline  C: 127.0^10^  I: 123.8^10^  End line  C: 132.0^10^  I: 131.5^10^  Grade 4  Baseline  C: 131.8^10^  I: 129.8^10^  End line  C: 136.6^10^  I: 136.9^10^ | NR | NR | Grade 1  Baseline  C: -0.65^10^  I: -0.60^10^  End line  C: -0.66^10^  I: -0.43^10^  Grade 2  Baseline  C: -0.29^10^  I: -0.39^10^  End line  C: -0.42^10^  I: -0.32^10^  Grade 3  Baseline  C: -0.45^10^  I: -0.58^10^  End line  C: -0.67^10^  I: -0.76^10^  Grade 4  Baseline  C: -0.23^10^  I: -0.63^10^  End line  C: -0.51^10^  I: -0.77^10^ | NR | Grade 1  Baseline  C: -0.94^10^  I: -1.02^10^  End line  C: -1.08^10^  I: -1.05^10^  Grade 2  Baseline  C: -0.68^10^  I: -0.91^10^  End line  C: -0.84^10^  I: -0.95^10^  Grade 3  Baseline  C: -0.83^10^  I: -1.05^10^  End line  C: -0.85^10^  I: -0.98^10^  Grade 4  Baseline  C: -0.87^10^  I: -0.94^10^  End line  C: -0.94^10^  I: -1.02^10^ | NR | NR | |  |
| Do et al. 2009 (4)  *Viet Nam* | | 454 | 7-8 y  Healthy | Milk ǂ  5.5 mg/d, 6 mo | Baseline:  C: 18.8 ± 2.1  I: 18.9 ± 2.2  End line:  C: 20.3 ± 2.4  I: 20.5 ± 2.4 | | Baseline:  C: 117.4 ± 4.9  I: 117.3 ± 5.2  End line:  C: 121 ± 5.0  I: 120.9 ± 5.2 | Baseline:  C: -1.29 ± 0.69  I: -1.17 ± 0.65  End line:  C: -1.14 ± 0.68  I: -1.05 ± 0.75 | Baseline:  C: 13.8  I: 9.9  End line:  C: 9.2  I: 8.6 | Baseline:  C: -1.70 ± 0.83  I: -1.64 ± 0.78  End line:  C: -1.52 ± 0.79  I: -1.49 ± 0.81 | Baseline:  C: 40.5  I: 32.4  End line:  C: 30.7  I: 23 | Baseline:  C: -1.95 ± 0.64  I:- 1.85 ± 0.61  End line:  C: -1.76 ± 0.61  I: -1.68 ± 0.64 | Baseline:  C: 49.6  I: 42.4  End line:  C: 36.6  I: 32.4** | NR | |  |
| Petrova et al. 2019 (5)  *Spain* | | 103^[[11]](#endnote-11)^ | 8-14 y | 13.5 mg/d, 5 mo | Baseline:  C: 33.2 ± 8.28  I: 30.56 ± 8.81  End line:  C: 34.24 ± 9.32  I: 29.54 ± 8.65 | | Baseline:  C: 133 ± 7.73  I: 130.92 ± 6.5  End line:  C: 135.67 ± 8.01  I: 133.18 ± 6.47 | NR | NR | NR | NR | NR | NR | NR | |  |
| Bardosono et al. 2009 (6)  *Indonesia* | | 245 | 7-9 y  Healthy | Milk powder ǂ  2.38 mg/d, 6 mo | C: 1.13 ± 0.69^11^  I: 1.31 ± 0.69^11^ | | C: 2.85 ± 0.88^11^  I: 3.03 ± 1^11^ | NR | C: 0^11^  I: 4.1^11^ | C: 1.18 ± 0.39^11^  I: 1.22 ± 0.42^11^ | C: 3.2^11^  I: 7.4^11^ | C: 1.33 ± 0.47^11^  I: 1.47 ± 0.50*^11^ | C: 10.5^11^  I: 18.2^11^ | C: 0.24 ± 0.68^11^  I: 0.39 ± 0.62^11^ | |  |
| Trinidad et al. 2015 (7)  *Philippines* | | 124 | ≥6 y  Healthy | Milk powder ǂ  NR, 4 mo | *1 glass*  Baseline:  18.4 ± 0.5  End line:  19.2 ± 0.6*  *2 glasses*  Baseline:  18.3 ± 0.3  End line:  19.2 ± 0.4*  *Water*  Baseline:  18.0 ± 0.3  End line:  18.5 ± 0.3* | | *1 glass*  Baseline:  111.5 ± 0.8  End line:  114.4 ± 0.7*  *2 glasses*  Baseline:  111.7 ± 0.7  End line:  115.0 ± 0.7*  *Water*  Baseline:  111.4 ± 0.6  End line:  114.5 ± 0.7* | NR | NR | NR | NR | NR | NR | NR | |  |
| Sazawal et al. 2010 (8)  Dhingra et al. 2004 (9)  *India* | | 524 | 1-3 y | Milk powder ǂ  9.6 mg/d, 24 mo | NR | | NR | C: 0.3 ± 0.65^11^  I: 0.42 ± 0.65*^11^ | NR | C: 0.09 ± 0.42^11^  I: 0.28 ± 0.47**^11^ | NR | C:0.18 ± 0.51^11^  I:0.38 ± 0.54**^11^ | NR | NR | |  |
| Sazawal et al. 2013 (10)  *India* | | 524 | 6-9 y | Yogurt ǂ  3 mg/d, 12 mo | NR | | NR | NR | NR | C: -1.17 ± 1.15^11^  I: -1 ± 1.12*^11^ | End line:  C: 23.2^11^  I: 17.9^11^ | C: -1.54 ± 1.21^11^  I: -1.51 ± 1.2^11^ | End line:  C: 20.8^11^  I: 24.1^11^ | NR | |  |
| *Cereal grains and condiments (n=7)* | | | | | | | | | | | | | | |  |  |
| Saldamli et al. 1996 (11)  *Turkey* | | 24 | 7-11 y  Healthy | Wheat flour, bread  54.4 mg/d^[[12]](#endnote-12)^, 3 mo | Baseline:  C: 131.2 ± 10.1  I: 128.5 ±9.7  End line:  C: 133.9 ± 11.1  I: 132.3 ± 10.2 | | Baseline:  C: 26. 8 ± 5.2  I: 27.6 ± 8.7  End line:  C: 27.3 ± 7.2  I: 29.6 ± 9.1 | NR | NR | NR | NR | NR | NR | NR | |  |
| Nga et al. 2011 (12)  *Viet Nam* | | 466 | 6-8 y  Healthy | Wheat flour, biscuits ǂ  5.6 mg/d, 4 mo  * Co-intervention: deworming (400mg Albendazole) or placebo pill | NR | | NR | *Control*  Baseline:  -0.94 ± 0.72  End line:  -0.86 ± 0.72**  *Fortified biscuits* Baseline:  -0.82 ± 0.78  End line:  -0.72 ± 0.76**  *Deworming*  Baseline:  -0.79 ± 0.81  End line:  -0.70 ± 0.80**  *Fortified & deworming*  Baseline:  -0.88 ± 0.78  End line:  -0.78 ± 0.76** | NR | *Control*  Baseline:  -1.41 ± 0.86  End line:  -1.34 ± 0.85**  *Fortified*  *biscuits*  Baseline:  -1.47 ± 0.78  End line:  -1.39 ± 0.79**  *Deworming*  Baseline:  -1.40 ± 0.82  End line:  -1.33 ± 0.81**  *Fortified & deworming*  Baseline:  -1.44 ± 0.86  End line:  -1.36 ± 0.85** | NR | *Control*  Baseline:  -1.56 ± 0.68  End line:  -1.48 ±0.70**  *Fortified*  *biscuits*  Baseline:  -1.52 ± 0.74  End line:  -1.42 ±0.71**  *Deworming*  Baseline:  -1.47 ± 0.72  End line:  -1.37 ±0.73**  *Fortified & deworming*  Baseline:  -1.55 ± 0.68  End line:  -1.44 ± 0.62** | NR | *Control*  Baseline:  15.0 ± 1.1  End line:  15.3 ± 1.2  *Fortified*  *biscuits*  Baseline:  15.1 ± 1.1  End line:  15.4 ± 1.1  *Deworming*  Baseline:  15.2 ± 1.2  End line:  15.6 ± 1.2  *Fortified & deworming*  Baseline:  15.0 ± 1.1  End line:  15.5 ± 1.1 | | |
| López de Romaña et al. 2005 (13)  *Peru* | | 31 | 3-4 y | Wheat flour, biscuits and noodles ǂ  0 mg/d, 3 mg/d, or 9 mg/d, 2.3 mo | *0 mg/100g:*  Baseline:  13.3 ± 1.9  End line:  14.8 ± 1.4  *3 mg/100g:*  Baseline:  13.8 ± 2.5  End line:  15.2 ± 2.3  *9 mg/100g:*  Baseline:  13.5 ± 1.7  End line:  14.5 ± 1.4 | | *0 mg/100g:*  Baseline:  90 ± 3.7  End line:  93.5 ± 3.7  *3 mg/100g:*  Base line:  91.2 ± 5.9  End line:  94.7 ± 5.5  *9 mg/100g:*  Base line:  89 ± 4  End line:  91.7 ± 4.6 | *0 mg/100g:*  Baseline:  -0.14 ± 1.07  End line:  0.56 ± 0.66  *3 mg/100g:*  Baseline:  0.1 ± 1.44  End line:  0.58 ± 1.17  *9 mg/100g:*  Baseline:  0.37 ± 1.1  End line:  0.17 ± 0.49 | NR | *0 mg/100g:*  Baseline:  -2.23 ± 0.9  End line:  -1.69 ± 1.09  *3 mg/100g:*  Baseline:  -2.77 ± 0.68  End line:  -2.43 ± 0.59  *9 mg/100g:*  Baseline:  -3.03 ± 0.58  End line:  -2.56 ± 0.7 | NR | *0 mg/100g:*  Baseline:  -1.45 ± 0.92  End line:  -1.04 ± -0.99  *3 mg/100g:*  Baseline:  -1.54 ± 1.12  End line:  -1.04 ± 0.92  *9 mg/100g:*  Baseline:  -1.48 ± 0.87  End line:  -0.9 ± 0.61 | NR | NR | | |
| Muthayya et al. 2009 (14)  *India* | | 550 | 6-10 y  Healthy | Wheat biscuit & milk powder ǂ  Co-fortification with n-3 fatty  acids  10.5 mg/d or 1.7 mg/d, 12 mo | *High MMN*  Baseline:  21.5 ± 3.6  End line:  25.2 ± 5.1  *Low MMN*  Baseline  21.6 ± 4  End line  25.4 ± 5.6 | | *High MMN*  Baseline:  123.1 ± 7.7  End line:  129.3 ± 7.8  *Low MMN*  Baseline  123 ± 7.8  End line  129 ± 7.8 | NR | NR | *High MMN*  Baseline:  -1.29 ± 0.83  End line:  -1.16 ± 0.82  *Low MMN*  Baseline  -1.33 ± 0.95  End line  -1.19 ± 0.93 | *High MMN*  Baseline:  22  End line:  14  *Low MMN*  Baseline  27  End line  18 | *High MMN*  Baseline:  -1.58 ± 0.68  End line:  -1.29 ± 0.77  *Low MMN*  Baseline  -1.61 ± 0.8  End line  -1.31 ± 0.88 | *High MMN*  Baseline:  30  End line:  17  *Low MMN*  Baseline  35.5  End line  20 | *High MMN*  Baseline:  17.1 ± 1.4  End line:  17.7 ± 2.0  *Low MMN*  Baseline  17.0 ± 1.7  End line  17.7 ± 2.1 | | |
| Manger et al. 2008 (15)  *Thailand* | | 555 | 5.5-13.4 y  Healthy | Seasoning powder ǂ  5 mg/d, 7.75 mo | Baseline:  C: 24.9 ± 6.95  I: 25 ± 7.24  End line:  C: 27.1 ± 7.72  I: 27 ± 8.03 | | Baseline:  C: 127.3 ± 10.07  I: 127.5 ± 10.44  End line:  C: 131.1 ± 10.31  I: 131.3 ± 10.77 | Baseline:  C: -0.78 ± 0.90  I: -0.74 ± 0.93  End line:  C: -0.74 ± 0.98  I: -0.78 ± 0.86 | Baseline:  C: 4.8  I: 4.0  End line:  C: 7.6  I: 4.5 | Baseline:  C: -1.05 ± 0.87  I: -1.07 ± 0.83  End line:  C: -0.98 ± 0.85  I: -1.05 ± 0.87 | Baseline:  C: 14.1  I: 11.6  End line:  C: 11.3  I: 11.2 | Baseline:  C: -1.18 ± 0.92  I: -1.20 ± 0.88  End line:  C: -1.08 ± 0.92  I: -1.18 ± 0.92 | Baseline:  C: 14.5  I: 11.3  End line:  C: 12.1  I: 10.3 | Baseline:  C: 18.2 ± 2.52  I:18.2 ± 2.53  End line:  C:18.8 ± 2.72  I: 18.7 ± 2.63 | | |
| Chen et al. 2008 (16)  *China* | | 226 | 2-6 y  Healthy | Seasoning powder ǂ  Vitamin A only (VA)  Vitamin A + iron (VAFe)  MMN  12 mg/d, 6 mo | NR | | NR | VA: 0.28**^[[13]](#endnote-13)^  VAFe: -0.31**^13^  MMN: 0.12**^13^ | NR | VA: 0.17**^13^  VAFe: 0.08**^13^  MMN: 0.48**^13^ | NR | VA:0.40**^13^  VA+Fe: -0.21**^13^  MMN: 0.31**^13^ | NR | NR | | |
| Thankachan et al. 2013 (17)  *India* | | 243 | 6-12 y  Healthy | Beverage ǂ  1.2 mg/d, 2 mo | Baseline:  C: 25 ± 6  I: 24.7 ± 6.4  End line:  C: 25.6 ± 6.5  I: 25.2 ± 6.8 | | Baseline:  C: 128 ± 1  I: 127 ± 9  End line:  C: 129 ± 1  I: 128 ± 1 | NR | NR | Baseline:  C: -1.06 ± 1.15  I:-1.19 ± 0.93  End line:  C: -1.09 ± 1.15  I: -1.19 ± 0.94 | Baseline:  C: 21.8  I: 18.9  End line:  C: 21.1  I: 18.3 | Baseline:  C: -1.19 ± 1.11  I: -1.32 ± 0.95  End line:  C: -1.18 ± 1.16  I: -1.36 ± 0.96 | Baseline:  C: 22.7  I: 29.1  End line:  C: 21.4  I: 28.9 | Baseline:  C: 18.2 ± 2.2  I: 18.2 ± 2.4  End line:  C: 18.4 ± 2.2  I: 18.2 ± 2.5 | | |
| *Non-controlled effectiveness studies (n=2)* | | | | | | | | | | | | | | | |  |
| Varea et al. 2011 (18)  *Argentina* | 472-474 | | 1-6 y  Healthy | Maize flour, soy-enriched ǂ  30 mg/kg, 12 mo  Average intake:  1-2 y: 6.1 mg/d  2-6 y: 6.2 mg/d | | NR | NR | NR | 1-2 y:  Pre: 1.9  Post:1.9  2-6 y:  Pre: 1.7  Post: 0.8 | NR | 1-2 y:  Pre: 12.8  Post: 6.3  2-6 y:  Pre: 8.7  Post: 10.8 | NR | 1-2 y:  Pre: 2.8  Post:1.3  2-6 y:  Pre: 2.3  Post: 3.6 | NR | |  |
| Engle-Stone et al. 2017 (19)  *Cameroon* | 255-300 | | 12-59 mo  Healthy | Wheat flour ǂ  73.6 ± 43.0 mg/kg  Expected intake: 7.5 mg/d | | NR | NR | Pre: 0.36 ± 0.05  Post: 0.42 ± 0.07 | Pre: 1  Post: 1.4 | Pre: -0.68 ± 0.07  Post: -0.63 ± 0.07 | Pre: 13  Post:15.6 | Pre: -0.12 ± 0.05  Post: -0.06 ± 0.06 | Pre: 1.4  End line: 5.7* | NM | |  |

**References**

1. Abrams SA, Mushi A, Hilmers DC, Griffin IJ, Davila P, Allen L. A multinutrient-fortified beverage enhances the nutritional status of children in Botswana. Journal of Nutrition. 2003;133(6):1834–40.

2. Angeles-Agdeppa I, Magsadia CR, Capanzana MV. Fortified juice drink improved iron and zinc status of schoolchildren. Asia Pacific Journal of Clinical Nutrition. 2011;20(4):535–43.

3. Rameshwar Sarma KV, Udaykumar P, Balakrishna N, Vijayaraghavan K, Sivakumar B. Effect of micronutrient supplementation on health and nutritional status of schoolchildren: Growth and morbidity. Nutrition. 2006;22(1 SUPPL.):S8–14.

4. Do TKL, Bui TN, Nguyen CK, Le TH, Nguyen TQN, Nguyen TH, et al. Impact of milk consumption on performance and health of primary school children in rural Vietnam. Asia Pacific Journal of Clinical Nutrition. 2009;18(3):326–34.

5. Petrova D, Bernabeu Litrán MA, García-Mármol E, Rodríguez-Rodríguez M, Cueto-Martín B, López-Huertas E, et al. Еffects of fortified milk on cognitive abilities in school-aged children: results from a randomized-controlled trial. European Journal of Nutrition. 2019;58(5):1863–72.

6. Bardosono S, Dewi LE, Sukmaniah S, Permadhi I, Eka AD, Lestarina L. Effect of a six-month iron-zinc fortified milk supplementation on nutritional status, physical capacity and speed learning process in Indonesian underweight schoolchildren: Randomized, placebo-controlled. Medical Journal of Indonesia. 2009;18(3):193–202.

7. Trinidad TP, Mallillin AC, Sagum RS, de Leon MP, Borlagdan MS, Baquiran AFP. Fortified milk consumption among 6-year old children: changes in biochemical markers of trace minerals and vitamins. Trace Elements and Electrolytes. 2015;32(3):112–8.

8. Sazawal S, Dhingra U, Dhingra P, Hiremath G, Sarkar A, Dutta A, et al. Micronutrient fortified milk improves iron status, anemia and growth among children 1-4 years: A double masked, randomized, controlled trial. PLoS ONE [Internet]. 2010;5(8). Available from: https://www.scopus.com/inward/record.uri?eid=2-s2.0-77957861492&doi=10.1371%2fjournal.pone.0012167&partnerID=40&md5=7c3940d6762d5c82fbccb481dc8cce42

9. Dhingra P, Menon VP, Sazawal S, Dhingra U, Marwah D, Sarkar A, et al. Effect of fortification of milk with zinc and iron along with vitamins C, E, A and selenium on growth, iron status and development in preschool children - A community based double-masked randomized trial [Internet]. 2004. 53 p. Available from: ://WOS:000227354700008

10. Sazawal S, Habib A, Dhingra U, Dutta A, Dhingra P, Sarkar A, et al. Impact of micronutrient fortification of yoghurt on micronutrient status markers and growth - a randomized double blind controlled trial among school children in Bangladesh. BMC Public Health. 2013;13:514.

11. Saldamli I, Ozalp I, Kilic I, Koksel H, Ozboy O. Zinc-supplemented bread and its utilization in zinc deficiency. Cereal Chemistry. 1996;73(4):424–7.

12. Nga TT, Winichagoon P, Dijkhuizen MA, Khan NC, Wasantwisut E, Wieringa FT. Decreased Parasite Load and Improved Cognitive Outcomes Caused by Deworming and Consumption of Multi-Micronutrient Fortified Biscuits in Rural Vietnamese Schoolchildren. American Journal of Tropical Medicine and Hygiene. 2011;85(2):333–40.

13. López de Romaña D, Peerson JM, Krebs NF, Brown KH, Salazar M, Hambidge KM, et al. Longitudinal measurements of zinc absorption in Peruvian children consuming wheat products fortified with iron only or iron and 1 of 2 amounts of zinc. American Journal of Clinical Nutrition. 2005;81(3):637–47.

14. Muthayya S, Eilander A, Transler C, Thomas T, van der Knaap HCM, Srinivasan K, et al. Effect of fortification with multiple micronutrients and n-3 fatty acids on growth and cognitive performance in Indian schoolchildren: the CHAMPION (Children’s Health and Mental Performance Influenced by Optimal Nutrition) Study. American Journal of Clinical Nutrition. 2009;89(6):1766–75.

15. Manger MS, McKenzie JE, Winichagoon P, Gray A, Chavasit V, Pongcharoen T, et al. A micronutrient-fortified seasoning powder reduces morbidity and improves short-term cognitive function, but has no effect on anthropometric measures in primary school children in northeast Thailand: A randomized controlled trial. American Journal of Clinical Nutrition. 2008;87(6):1715–22.

16. Chen K, Li TY, Chen L, Qu P, Liu YX. Effects of vitamin A, vitamin A plus iron and multiple micronutrient-fortified seasoning powder on preschool children in a suburb of Chongqing, China. Journal of Nutritional Science and Vitaminology. 2008;54(6):440–7.

17. Thankachan P, Selvam S, Surendran D, Chellan S, Pauline M, Abrams SA, et al. Efficacy of a multi micronutrient-fortified drink in improving iron and micronutrient status among schoolchildren with low iron stores in India: A randomised, double-masked placebo-controlled trial. European Journal of Clinical Nutrition. 2013;67(1):36–41.

18. Varea A, Malpeli A, Etchegoyen G, Vojkovic M, Disalvo L, Apezteguia M, et al. Short-Term Evaluation of the Impact of a Food Program on the Micronutrient Nutritional Status of Argentinean Children Under the Age of Six. Biological Trace Element Research. 2011;143(3):1337–48.

19. Engle-Stone R, Nankap M, Ndjebayi AO, Allen LH, Shahab-Ferdows S, Hampel D, et al. Increases in indicators of iron, zinc, folate and vitamin B12 status in urban Cameroon following wheat flour fortification. FASEB Journal Conference: Experimental Biology [Internet]. 2017;31(1 Supplement 1). Available from: http://www.fasebj.org/content/31/1_Supplement/436.8.abstract?sid=7df75b15-2204-4fa2-9c05-1cfe1ef47719 http://ovidsp.ovid.com/ovidweb.cgi?T=JS&CSC=Y&NEWS=N&PAGE=fulltext&D=emed18&AN=616960029 https://4223523.odslr.com/resolver/full?sid=OVID:embase&id=pmid:&id=doi:&issn=1530-6860&isbn=&volume=31&issue=1+Supplement+1&spage=&pages=&date=2017&title=FASEB+Journal&atitle=Increases+in+indicators+of+iron%2C+zinc%2C+folate+and+vitamin+B12+status+in+urban+Cameroon+following+wheat+flour+fortification&aulast=Engle-Stone&pid=%3Cauthor%3EEngle-Stone+R.%3BNankap+M.%3BNdjebayi+A.O.%3BAllen+L.H.%3BShahab-Ferdows+S.%3BHampel+D.%3BKillilea+D.W.%3BGimou+M.-M.%3BHoughton+L.A.%3BFriedman+A.%3BTarini+A.%3BStamm+R.%3BBrown+K.H.%3C%2Fauthor%3E%3CAN%3E616960029%3C%2FAN%3E%3CDT%3EConference+Abstract%3C%2FDT%3E

1. Abbreviations: C, control group; HAZ, Height-for-age Z-score; I, intervention group; MMN, multiple micronutrients; MUAC, Mid-Upper Arm Circumference; NM, not measured; NR, not reported; WAZ, Weight-for-age-Z-score; WHZ, Weight-for-height Z-score;

   ǂ the food was fortified with multiple micronutrients

   **P*<0.05

   ***P* <0.01

   Anthropometric data was extracted for children ages 0-10. The World Health Organization’s definition of a child is as follows: “A child is a person 19 years or younger unless national law defines a person to be an adult at an earlier age. However, in these guidelines when a person falls into the 10 to 19 age category they are referred to as an adolescent” [↑](#endnote-ref-1)
2. Sample size included in analysis [↑](#endnote-ref-2)
3. Population characteristics included are age and health status, as reported by authors. Age is a range, unless footnoted otherwise. [↑](#endnote-ref-3)
4. If unit is expressed as mg/kg, the value is referring to fortification level. If unit is expressed as mg/day, the value is referring to dose. Where fortification level was reported, dose is calculated based on reported/expected intake of the food. [↑](#endnote-ref-4)
5. Durations were converted to months using the following methodology: 4 weeks=1 month, 30 days=1 month, 1 year=12 months [↑](#endnote-ref-5)
6. Values are mean ± SD unless footnoted otherwise; Baseline/end line used to refer to studies where the same individuals were followed-up; Pre/post used to refer to studies where the pre and post measurements occurred in different individuals sampled from the same population. [↑](#endnote-ref-6)
7. Dhingra 2004 and Sazawal 2010 presented the same anthropometric data, therefore, this study was only counted once [↑](#endnote-ref-7)
8. Reported as change value [↑](#endnote-ref-8)
9. School grades 1-9 were included in the study, however, for the purposes of child anthropometry we only include grades which have an average age less than 10 years [↑](#endnote-ref-9)
10. Mean [↑](#endnote-ref-10)
11. Mean difference [↑](#endnote-ref-11)
12. Calculated by review authors; authors stated a fortification level of 3 grams/1kg and an intended daily dose of 2 mg/kg of body weight. Dose/day was calculated using the average of the treatment and control groups’ weight at baseline (27.6 kg for treatment, 26.8 kg for control) [↑](#endnote-ref-12)
13. Median change [↑](#endnote-ref-13)
