# Supplementary material for: Effects of Foods Fortified with Zinc, Alone or Cofortified with Multiple Micronutrients, on Health and Functional Outcomes: A Systematic Review and Meta-Analysis
Source: Adv Nutr. 2021 Jun 24;12(5):1821–37. doi: 10.1093/advances/nmab065 (PMC8483949; doi:10.1093/advances/nmab065)
Supplement: nmab065_Supplemental_Files [file nmab065_supplemental_files.zip › Supplemental Table 8. Cognition.docx]

**Table S8. Effect of foods fortified with zinc, alone or co-fortified with multiple micronutrients,** **on cognition^[[1]](#endnote-1)^**

| Reference^[[2]](#endnote-2)^  *Study location* | *n* | Population^[[3]](#endnote-3)^ | Zinc fortified food | Zinc dose, duration^[[4]](#endnote-4)^ | Cognitive test(s)^[[5]](#endnote-5)^ | | | | |
| --- | --- | --- | --- | --- | --- | --- | --- | --- | --- |
| *Efficacy Studies (n=11)* | | | | | | | | | |
| Vazir et al. 2006 (1)  *India* | 608 | 6-18 y | Beverage ǂ | 2.3 mg/d,  14 mo | *Memory*  Baseline:  C: 97.9^[[6]](#endnote-6)^  I: 100^6^  End line  C: 98^6^  I: 99.8^6^ | *Intelligence (IQ)*  Baseline:  C: 99.2^6^  I: 101.3^6^  End line  C: 107.7^6^  I: 108.8^6^ | *Knox Cube test*  Baseline:  C: 99.1^6^  I: 101.2^6^  End line  C: 98.3^6^  I: 102.2**^6^ | *Scholastic achievement*  Quarter:  C: 64.2^6^  I: 66.3^6^  Final:  C: 59.8^6^  I: 60.4^6^ | *Letter cancellation*  Baseline:  C: 98.9^6^  I: 99.9^6^  End line  C: 100.4^6^  I: 98.3^6^ |
| Bardosono et al. 2009 (2)  *Indonesia* | 245 | 7-9 y  Healthy | Milk powder ǂ | 2.38 mg/d,  6 mo | *Coding change*:  C: 8.31 ± 9.60  I: 12.74 ± 11.76 | *Digit-span Forward change*  C: 0.88 ± 2.69  I: 1.07 ± 3.15 | *Digit-span backward change*  C: 0.69 ± 2.30  I: 1.10 ± 2.13 | *Visual search*  C: 7.67 ± 12.11  I: 7.43 ± 7.01 |  |
| Costarelli et al. 2014 (3)  *Italy* | 21 | ≥ 82 y  Healthy | Milk powder | 4 mg/d,  2 mo | *AMTS*  Baseline: 8.33 ± 0.24  End line: 8.19 ± 0.48 |  |  |  |  |
| Fiorentino et al. 2018 (4)  *Cambodia* | 1,796 | 6-16 y  Healthy | Rice ǂ:  UtraRice Original (URO)  UltraRice New (URN)  NutriRice (NR) | URO:  3.5 mg/d  URN:  2.3 mg/d  NR:  4.2 mg/d  7 mo |  |  | *WISC III Picture Completion*  Baseline:  URO: 7.9 ± 1.9  URN: 7.5 ± 1.8  NR: 7.8 ± 1.8  C: 7.6 ± 1.9  End line:  URO: 11.0 ± 1.9  URN: 10.9 ± 1.9  NR: 10.4 ± 1.9  C: 10.7 ± 1.9 | *WISC III Block Design*  Baseline:  URO: 13.6 ± 0.4  URN: 14.1 ± 0.4  NR: 13.6 ± 0.4  C: 13.1 ± 0.4  End line:  URO: 22.0 ± 0.5*  URN: 20.5 ± 0.5  NR: 20.4 ± 0.5  C: 20.5 ± 0.5 | *RCPM*  Baseline:  URO: 17.5 ± 0.2  URN: 17.1 ± 0.2  NR: 16.4 ± 0.2  C:16.6 ± 0.2  End line:  URO: 21.0 ± 0.2  URN: 20.1 ± 0.2  NR: 19.9 ± 0.2  C: 20.1 ± 0.2 |
| Manger et al. 2008 (5)  *Thailand* | 555 | 5.5-13.4 y  Healthy | Seasoning powder ǂ | 5 mg/d,  7.75 mo |  | *WISC III Digit-span Forward*  C: 9.1 ± 1.94  I: 9.3 ± 2.25 | *WISC III Digit-span Backward*  C: 3.3 ± 1.53  I: 3.2 ± 1.56 | *Visual Recall*  C: 9.4 ± 2.5  I: 10.0 ± 2.30** |  |
| Do et al. 2009 (6)  *Vietnam* | 454 | 7-8 y  Healthy | Milk ǂ | 5.5 mg/d,  6 mo | NR | | | | |
| Nga et al. (7) 2011  *Vietnam* | 466 | 6-8 y  Healthy | Wheat flour biscuits ǂ  Co-intervention:  deworming (400mg  Albendazole, Alb) or placebo  Pill (PP) | 5.6 mg/d,  4 mo | *WISC III Coding*  C biscuits + PP  Baseline: 31.9±10.0  End line: 39.6±8.0  I + PP  Baseline: 30.8±8.4  End line: 39.5±7.0  C biscuits + Alb  Baseline: 32.2±9.4  End line: 39.6±7.8  I + Alb  Baseline: 30.8±8.6  End line: 39.5±8.5 | *WISC III Digit-span Forward*  PP biscuits + PP  Baseline: 2.9 ± 0.9  End line: 3.1 ± 0.8  I + PP  Baseline: 2.9 ± 1.0  End line: 3.1 ± 0.9  C biscuits + Alb  Baseline: 2.8 ± 1.0  End line: 3.1 ± 0.8  I + Alb  Baseline: 2.9 ± 0.8  End line: 3.0 ± 0.9 | *WISC III Digit-span Backward*  C biscuits + PP  Baseline: 7.1 ± 1.4  End line: 7.1 ± 1.4  I + PP  Baseline: 6.0 ± 1.2  End line: 7.5 ± 1.1  C biscuits + Alb  Baseline: 6.9 ± 1.5  End line: 7.1 ± 1.3  I + Alb  Baseline: 7.1 ± 1.2  End line: 7.4 ± 1.1 | *WISC III Block design*  C biscuits + PP  Baseline: 11.8 ± 8.5  End line: 16.6 ± 9.0  I + PP  Baseline: 12.5 ± 8.7  End line: 16.5 ± 8.6  C biscuits + Alb  Baseline: 11.1 ± 8.4  End line: 16.9 ± 9.6  I + Alb  Baseline: 12.8 ± 7.4  End line: 17.0 ± 9.1 | *RCPM*  C biscuits + PP  Baseline: 16.4 ±5 .6  End line: 19.2 ± 5.8  I + PP  Baseline: 16.5 ± 5.0  End line: 20.1 ± 4.9  C biscuits + Alb  Baseline: 16.4 ± 5.3  End line: 19.2 ± 5.4  I + Alb  Baseline: 16.5 ± 4.9  End line: 19.4 ± 5.0 |
| Muthayya et al. 2009 (8)  *India* | 548 | 6-10 y  Healthy | Wheat flour biscuit & milk powder ǂ  Co-fortification  with n-3 fatty  acids | High MMN  High n-3:  10.5 mg/d  High MMN  Low n-3:  10.5 mg/d  Low MMN  High n-3:  1.7 mg/d  Low MMN  Low n-3:  1.7 mg/d  12 mo | *Mental Processing Index*  High MMN/High n-3:  Baseline: -0.01 ± 0.67  End line: 0.62 ± 0.32  High MMN/Low n-3:  Baseline: 0.00 ± 0.66  End line: 0.63 ± 0.32  Low MMN/High n-3:  Baseline: 0.01 ± 0.63  End line: 0.65 ± 0.30  Low MMN/Low n-3:  Baseline: 0.02 ± 0.64  End line: 0.64 ± 0.31 | *KABC-II Short-term Memory*  High MMN/High n-3:  Baseline: 0.08 ± 1.04  End line: 0.42 ± 0.60  High MMN/Low n-3:  Baseline: -0.02 ± 0.85  End line: 0.43 ± 0.54  Low MMN/High n-3:  Baseline: -0.02 ± 0.82  End line: 0.37 ± 0.66  Low MMN/Low n-3:  Baseline: -0.03 ± 0.87  End line: 0.38 ± 0.65 | *KABC-II Fluid Reasoning*  High MMN/High n-3:  Baseline: -0.06 ± 0.71  End line: 0.72 ± 0.48  High MMN/Low n-3:  Baseline: 0.02 ± 0.82  End line: 0.70 ± 0.45  Low MMN/High n-3:  Baseline: 0.02 ± 0.81  End line: 0.84 ± 0.51  Low MMN/Low n-3:  Baseline: 0.04 ± 0.81  End line: 0.82 ± 0.47 | *KABC-II Retrieval Ability*  High MMN/High n-3:  Baseline: -0.04 ± 0.79  End line: 0.55 ± 0.61  High MMN/Low n-3:  Baseline: 0.04 ± 0.80  End line: 0.59 ± 0.60  Low MMN/High n-3:  Baseline: 0.01 ± 0.73  End line: 0.60 ± 0.51  Low MMN/Low n-3:  Baseline: 0.00 ± 0.74  End line: 0.58 ± 0.56 | *KABC-II Cognitive Speediness*  High MMN/High n-3:  Baseline: -0.01 ± 0.88  End line: 0.80 ± 0.61  High MMN/Low n-3:  Baseline: -0.05 ± 0.89  End line: 0.81 ± 0.57  Low MMN/High n-3:  Baseline: 0.02 ± 0.89  End line: 0.79 ± 0.56  Low MMN/Low n-3:  Baseline: 0.05 ± 0.87  End line: 0.76 ± 0.59 |
| Vinodkumar et al. 2009 (9)  *India* | 371 | I: 12.21 y  C: 12.27 y | Salt ǂ | 10 mg/d,  9 mo | *Memory test*  Baseline:  C: 12.94 ± 0.564  I: 13.7 ± 0.588  End line:  C: 14.16 ± 0.518*  I: 16.22 ± 0.414** |  |  |  |  |
| Petrova et al. 2019 (10)  *Spain* | 103 | 8-14 y  Healthy | Milk ǂ | 13.5 mg/d,  5 mo | *WISC IV-Coding*  Baseline:  C: 8.49 ± 0.36  I: 9.37 ± 0.42  End line:  C: 10.12 ± 0.3  I: 9.46 ± 0.29 | *WISC IV Digit-span (combined)*  Baseline:  C: 2.78 ± 0.06  I: 2.87 ± 0.08  End line:  C: 2.96 ± 0.04  I: 3.1 ± 0.4* | | *WISC IV Symbol/ Animal Search*  Baseline:  C: 9.33 ± 0.33  I: 9.69 ± 0.44  End line:  C: 9.75 ± 0.38  I: 9.88 ± 0.38 | *WISC IV Letter Number Sequencing*  Baseline:  C: 2.71 ± 0.1  I: 2.81 ± 0.04  End line:  C: 2.72 ± 0.07  I: 2.83 ± 0.07 |
| Aaron et al. 2011a (11)  *Nigeria* | 534 | 10 ± 2.2^[[7]](#endnote-7)^ y  Healthy | Beverage, precooked maize and soy protein isolate ǂ | 15 mg/d,  6 mo | “Cognitive assessments were also carried out; however, there were no effects of the intervention on cognitive abilities (not shown).” | | | | |

**References**

1. Vazir S, Nagalla B, Thangiah V, Kamasamudram V, Bhattiprolu S. Effect of micronutrient supplement on health and nutritional status of schoolchildren: mental function. Nutrition. 2006 Jan;22(1 Suppl):S26-32.

2. Bardosono S, Dewi LE, Sukmaniah S, Permadhi I, Eka AD, Lestarina L. Effect of a six-month iron-zinc fortified milk supplementation on nutritional status, physical capacity and speed learning process in Indonesian underweight schoolchildren: Randomized, placebo-controlled. Medical Journal of Indonesia. 2009;18(3):193–202.

3. Costarelli L, Giacconi R, Malavolta M, Basso A, Piacenza F, DeMartiis M, et al. Effects of zinc-fortified drinking skim milk (as functional food) on cytokine release and thymic hormone activity in very old persons: A pilot study. Age. 2014;36(3):1421–31.

4. Fiorentino M, Perignon M, Kuong K, de Groot R, Parker M, Burja K, et al. Effect of multi-micronutrient-fortified rice on cognitive performance depends on premix composition and cognitive function tested: results of an effectiveness study in Cambodian schoolchildren. Public Health Nutrition. 2018;21(4):816–27.

5. Manger MS, McKenzie JE, Winichagoon P, Gray A, Chavasit V, Pongcharoen T, et al. A micronutrient-fortified seasoning powder reduces morbidity and improves short-term cognitive function, but has no effect on anthropometric measures in primary school children in northeast Thailand: A randomized controlled trial. American Journal of Clinical Nutrition. 2008;87(6):1715–22.

6. Do TKL, Bui TN, Nguyen CK, Le TH, Nguyen TQN, Nguyen TH, et al. Impact of milk consumption on performance and health of primary school children in rural Vietnam. Asia Pacific Journal of Clinical Nutrition. 2009;18(3):326–34.

7. Nga TT, Winichagoon P, Dijkhuizen MA, Khan NC, Wasantwisut E, Wieringa FT. Decreased Parasite Load and Improved Cognitive Outcomes Caused by Deworming and Consumption of Multi-Micronutrient Fortified Biscuits in Rural Vietnamese Schoolchildren. American Journal of Tropical Medicine and Hygiene. 2011;85(2):333–40.

8. Muthayya S, Eilander A, Transler C, Thomas T, van der Knaap HCM, Srinivasan K, et al. Effect of fortification with multiple micronutrients and n-3 fatty acids on growth and cognitive performance in Indian schoolchildren: the CHAMPION (Children’s Health and Mental Performance Influenced by Optimal Nutrition) Study. American Journal of Clinical Nutrition. 2009;89(6):1766–75.

9. Vinodkumar M, Erhardt JG, Rajagopalan S. Impact of a multiple-micronutrient fortified salt on the nutritional status and memory of schoolchildren. International Journal for Vitamin and Nutrition Research. 2009;79(5–6):348–61.

10. Petrova D, Bernabeu Litrán MA, García-Mármol E, Rodríguez-Rodríguez M, Cueto-Martín B, López-Huertas E, et al. Еffects of fortified milk on cognitive abilities in school-aged children: results from a randomized-controlled trial. European Journal of Nutrition. 2019;58(5):1863–72.

11. Aaron GJ, Kariger P, Aliyu R, Flach M, Iya D, Obadiah M, et al. A Multi-Micronutrient Beverage Enhances the Vitamin A and Zinc Status of Nigerian Primary Schoolchildren. Journal of Nutrition. 2011;141(8):1565–72.

1. Abbreviations (in alphabetical order): AMTS, abbreviated mental test score; C, control group; I, intervention group; KABC, Kaufman Assessment Battery for Children; MMN, micronutrient; NR, NutriRice; RCPM, Raven’s Colored Progressive Matrices; URN, UltraRice New; URO, UltraRice Original; WISC, Wechsler Intelligence Scale for Children
   **P*<0.05
   ** *P* <0.01

   ǂ the food was fortified with multiple micronutrients

   Papers organized in table in order of dose (ascending) [↑](#endnote-ref-1)
2. Reference: Author, year of publication; Country of study location; UNICEF region [↑](#endnote-ref-2)
3. Population characteristics included are age and health status. Age is a range, unless footnoted otherwise. [↑](#endnote-ref-3)
4. Durations were converted to months using the following methodology: 4 weeks=1 month, 30 days=1 month, 1 year=12 months [↑](#endnote-ref-4)
5. Mean ± standard deviation unless footnoted otherwise [↑](#endnote-ref-5)
6. Mean [↑](#endnote-ref-6)
7. Mean ± standard deviation [↑](#endnote-ref-7)
