# Supplementary material for: Effects of Foods Fortified with Zinc, Alone or Cofortified with Multiple Micronutrients, on Health and Functional Outcomes: A Systematic Review and Meta-Analysis
Source: Adv Nutr. 2021 Jun 24;12(5):1821–37. doi: 10.1093/advances/nmab065 (PMC8483949; doi:10.1093/advances/nmab065)
Supplement: nmab065_Supplemental_Files [file nmab065_supplemental_files.zip › Supplemental Table 9. Morbidity.docx]

**Table S9. Effect of foods fortified with zinc, alone or co-fortified with multiple micronutrients,** **on morbidity outcomes (n=14)**

| Reference  *Study location* | *n*^[[1]](#endnote-1)^ | | Population  characteristics^[[2]](#endnote-2)^ | Zinc fortified food, control^[[3]](#endnote-3)^ | | Zinc dose/fortification level, duration^[[4]](#endnote-4),^^[[5]](#endnote-5)^ | | Respiratory Related | | Severe illness  /hospitalization | Diarrhea related | | Other | |
| --- | --- | --- | --- | --- | --- | --- | --- | --- | --- | --- | --- | --- | --- | --- |
| Liquid foods (*n=6*) | | | | | | | | | | | | | | |
| Aaron et al. 2011a (1) | 534 | | 10 ± 2.2^[[6]](#endnote-6)^ y  Healthy | Beverage, precooked maize and soy protein isolate | | 15 mg/d, 6 mo | | NS differences in self-reported prevalence of URTI (not shown) | | NM | NS differences in self-reported prevalence of diarrhea (not shown) | | NS differences in self-reported prevalence of malaria (not shown)  *Prevalence of vomiting at end of intervention (%):*  C: 14  I: 21 | |
| Angeles-Agdeppa et al.  2011 (2)  *Philippines* | 89 | | 6-9 y  Anemic | Beverage, orange flavored  juice | | 2.8 mg/d, 3.3 mo | | *Cough and colds during study (%)*  C: 58.7  I: 58.1 | | NM | Episodes of diarrhea similar in both groups (not shown). | | Episodes of fever similar in both groups (not shown). | |
| Rameshwar Sarma et al.  2006 (3)  *India* | 869 | | C: 10.2 ± 2.36 y  I: 10.1 ± 2.46 y | Beverage | | 4.6 mg/d, 12-14 mo | | *Cough and cold*  *(Episodes/child/y)*  C: 5.4  I: 5.3  *Respiratory (duration, mean d)*  C: 5.0 ± 7.89  I: 12.1 ± 12.47 | | NM | *Acute diarrhea*  *(Episodes/child/y)*  C: 1.2  I: 1  Gastroenteritis *(duration, mean d)*  C: 2.2 ± 5.09  I: 5.0 ± 7.89 | | *Angular Stomatitis (%/child/yr)*  Pre: 2.5  Post: 0.3  *Cheilosis (%/child/yr)*  Pre: 3.4  Post: 1.4  *Glossitis (%/child/yr)*  Pre: 2  Post: 0.3  *Bleeding gums (%/child/yr)*  Pre: 2  Post: 1.1  *Caries (%/child/yr)*  Pre: 10.1  Post: 5.9  *Mottled enamel (%/child/yr)*  Pre: 8.5  Post: 0  *Fever (episodes/child/y)*  C: 3.5  I: 3.3  *Fever (duration, mean d)*  C: 5.3 ± 1.96^[[7]](#endnote-7)^  I: 3.3 ± 1.56  *Ear infection (events/child/y)*  C: 0.6  I: 0.6 | |
| Costarelli et al. 2014 (7)  *Italy* | 21 | | ≥ 82 y  Healthy | Milk, skim  (cross-over study) | | 4 mg/d, 2 mo | | NM | | *Mortality (%)*  C: 52  I: 10  *Hospitalization, flu (%)*  C: 22  I: 10  *Hospitalization, falls (%)*  C: 9  I: 10 | NM | | NM | |
| Dhingra et al 2004 (8)  Marwah et al. 2004 (9)  Sazawal et al. 2007 (10)  *India* | 633 | | 1-3 y | Milk powder | | 9.6 mg/d, 12 mo | | *LRTI*  *Rate ratio (95% CI)*  0.74 (0.57, 0.97)* | | *Severe illness (d)*  *Rate ratio (95% CI)^[[8]](#endnote-8)^*  0.85 (0.76, 0.95)** | *Diarrhea episodes*  *Rate ratio (95% CI)*  0.82 (0.73, 0.93)**  *Dysentery*  *Rate ratio (95% CI)*  0.91 (0.67, 1.22) | | *Measles, Rate ratio (95% CI)*  0.12 (0.02, 0.99)*  High fever, *Rate ratio (95% CI)*  0.93 (0.88-0.98) | |
| Thomas et al. 2012 (12)  *India* | 546 | | 6-10 y  Healthy | I: High MMN fortified wheat biscuit &  milk powder  C: Low MMN  Co-fortification  with n-3 fatty  acids | | Dose:  High MMN/High n-3:  10.5 mg/d  High MMN/Low n-3:  10.5 mg/d  Low MMN/High n-3:  1.7 mg/d  Low MMN/Low n-3:  1.7 mg/d  12 mo | | *URTI*  High vs. low MMN:  *Rate ratio (95% CI)*  1.05 (0.94, 1.18)  High vs low n-3:  *Rate ratio (95% CI)*  0.88 (0.79, 0.97)*  *LRTI*  High vs. low MMN:  *Rate ratio (95% CI)*  1.05 (0.91, 1.20)  High vs low n-3:  *Rate ratio (95% CI)*  0.97 (0.85, 1.11) | | NM | *Gastrointestinal complaints*  High vs. low MMN:  *Rate ratio (95% CI)*  0.93 (0.83, 1.06)  High vs. low n-3:  *Rate ratio (95% CI)*  0.89 (0.79, 1.01) | | NM | |
| *Cereal grains and condiments (n=8)* | | | | | | | | | | | | | | |
| Manger et al. 2008 (4)  *Thailand* | 555 | 5.5-13.4 y  Healthy | | | Seasoning  powder | | 5 mg/d, 7.75 mo | | *Respiratory related*  *Rate ratio (95% CI):*  Episodes: 0.83 (0.73, 0.94)** | NM | *Diarrhea related*  *Rate ratio (95% CI):*  Episodes: 0.90 (0.59, 1.39) | *Fever, Rate ratio (95% CI)*  Episodes: 0.92 (0.74, 1.13)  *Skin rash, Rate ratio (95% CI)*  Episodes: 1.09 (0.47, 2.51)  *Other^[[9]](#endnote-9)^, Rate ratio (95% CI)*  Episodes: 1.02 (0.70, 1.48) | |  |
| Chen et al. 2011 (5)  *China* | 226 | 2-6 y  Healthy | | | I: Seasoning  powder  C: VAFe  fortified seasoning  powder | | 12 mg/d, 6 mo | | *Respiratory related*  *Rate ratio (95% CI):*  0.82 (0.77, 0.96) | NM | *Diarrhea related*  *Rate ratio (95% CI):*  0.78 (0.61, 0.92) | *Fever, Rate ratio (95% CI)*  Episodes: 0.80 (0.69, 0.97)  *Skin rash, Rate ratio (95% CI)*  Episodes: 0.97 (0.82, 1.26)  *Other^[[10]](#endnote-10)^, Rate ratio (95% CI)*  Episodes: 1.04 (0.88, 1.17) | |  |
| Vinodkumar et al. 2009 (6)  *India* | 371 | I:12.21 y  C: 12.27 y | | | I: MMN Salt  C: Iodized salt | | 10 mg/d, 9 mo | | NM | NM | NM | *Angular stomatitis*  *Risk ratio (95% CI)*  0.26 (0.12-0.56)**^[[11]](#endnote-11)^ | |  |
| Saldamli et al. 1996 (11)  *Turkey* | 24 | 8.71±1.22 y  Healthy | | | Wheat flour, bread | | 2 mg/kg of body weight/d, 3 mo | | NR | NM | NR | *Total number of infections (diarrhea, respiratory, pyoderma)*  C: 2.3 ± 1.6  I: 1 ± 0.9 | |  |
| Nieman et al. 2011 (13)  *United States* | 65 | 7-13 y  Heathy | | | Cereal, extruded puffed corn cereal  Co-intervention:  Pneumococcal  vaccine | | 0 mg/d,  12.5mg/d, or  25 mg/d,  2 mo | | *URTI (duration, mean d)*  Low: 8.8 ± 10.9  Medium: 10.4 ± 6.5  High: 7.4 ± 7.2  *Prevalence, >1 URTI episode during study period:*  Low: 83  Medium: 90  High: 74 | NM | NM | NM | |  |
| De Gier et al. 2016 (14)  *Cambodia* | 1,257 | NR  Healthy | | | Rice, 3 kinds brands using extrusion technology | | UltraRice Original (URO):  3.5 mg/d  UltraRice New (URN):  2.3 mg/d  NutriRice (NR):  4.2 mg/d  6 mo | | NM | NM | NM | *Hookworm infection (%)*  All de-wormed at baseline  3-mo:  Placebo: 12.1  URO: 16.4  URN: 18.8  NR: 14.9  7-mo:  Placebo: 11.9  URO: 24.6*  URN: 21.8*  NR: 16 | |  |
| Ara et al. 2019 (15)  *Bangladesh* | 800 | 15-49 y  Healthy | | | Rice | | 40 mg/kg; 30 kg of rice/mo provided per household for 1 y | | NM | NM | *Episodes (last 24 hours)*  Baseline:  C: 65±14.94  I: 56±12.87  End line:  C: 41±10.3  I: 31±7.6* | *Fever above 36-37*˚C  Baseline:  C:171(39.31)  I: 186 (42.76)  End line:  C: 135(33.3)  I: 105 (26.5)* | |  |
| Engle-Stone et al. 2017 (16)  *Cameroon* | Pre:  WRA: 279  PSAC: 272  Post:  WRA: 302  PSAC: 303 | WRA  15-49 y  Healthy  PSAC  12-59 mo | | | Wheat flour  (pre/post fortification) | | 73.6 ± 43.0 mg/kg | | NM | NM | NM | *Malaria (%)*  WRA  Pre: 7  Post: 5  PSAC  Pre: 13  Post: 8 | |  |

**References**

1. Aaron GJ, Kariger P, Aliyu R, Flach M, Iya D, Obadiah M, Baker SK. A Multi-Micronutrient Beverage Enhances the Vitamin A and Zinc Status of Nigerian Primary Schoolchildren. Journal of Nutrition. 2011;141:1565–72.

2. Angeles-Agdeppa I, Magsadia CR, Capanzana MV. Fortified juice drink improved iron and zinc status of schoolchildren. Asia Pacific Journal of Clinical Nutrition. 2011;20:535–43.

3. Rameshwar Sarma KV, Udaykumar P, Balakrishna N, Vijayaraghavan K, Sivakumar B. Effect of micronutrient supplementation on health and nutritional status of schoolchildren: Growth and morbidity. Nutrition. 2006;22:S8–14.

4. Manger MS, McKenzie JE, Winichagoon P, Gray A, Chavasit V, Pongcharoen T, Gowachirapant S, Ryan B, Wasantwisut E, Gibson RS. A micronutrient-fortified seasoning powder reduces morbidity and improves short-term cognitive function, but has no effect on anthropometric measures in primary school children in northeast Thailand: A randomized controlled trial. American Journal of Clinical Nutrition. 2008;87:1715–22.

5. Chen K, Wei X, Qu P, Liu Y, Zhang X, Li T, Chen L. Effect of vitamin A, vitamin A plus iron and multiple micronutrient-fortified seasoning powder on infectious morbidity of preschool children [electronic resource]. Nutrition. [New York]: Elsevier Science Inc; 2011;27:428–34.

6. Vinodkumar M, Erhardt JG, Rajagopalan S. Impact of a multiple-micronutrient fortified salt on the nutritional status and memory of schoolchildren. International Journal for Vitamin and Nutrition Research. 2009;79:348–61.

7. Costarelli L, Giacconi R, Malavolta M, Basso A, Piacenza F, DeMartiis M, Giannandrea E, Renieri C, Busco F, Galeazzi R, et al. Effects of zinc-fortified drinking skim milk (as functional food) on cytokine release and thymic hormone activity in very old persons: A pilot study. Age. Kluwer Academic Publishers; 2014;36:1421–31.

8. Dhingra P, Menon VP, Sazawal S, Dhingra U, Marwah D, Sarkar A, Verma P, Juyal R, Sood M, Black M, et al. Effect of fortification of milk with zinc and iron along with vitamins C, E, A and selenium on growth, iron status and development in preschool children - A community based double-masked randomized trial [Internet]. 2004. 53 p. Available from: ://WOS:000227354700008

9. Marwah D, Sazawal S, Dhingra U, Verma P, Deb S, Dhingra P, Sarkar A, Menon VP, Black RE, Medimond. Efficacy of micronutrient fortification of milk in prevention of childhood morbidity in children 1-3 years of age - A community based double masked randomized trial [Internet]. 2004. 367 p. Available from: ://WOS:000227354700062

10. Sazawal S, Dhingra U, Dhingra P, Hiremath G, Kumar J, Sarkar A, Menon VP, Black RE. Effects of fortified milk on morbidity in young children in north India: community based, randomised, double masked placebo controlled trial. Bmj. 2007;334:140.

11. Saldamli I, Ozalp I, Kilic I, Koksel H, Ozboy O. Zinc-supplemented bread and its utilization in zinc deficiency. Cereal Chemistry. 1996;73:424–7.

12. Thomas T, Eilander A, Muthayya S, McKay S, Thankachan P, Theis W, Gandhe A, Osendarp SJM, Kurpad AV. The effect of a 1-year multiple micronutrient or n-3 fatty acid fortified food intervention on morbidity in Indian school children. European Journal of Clinical Nutrition. 2012;66:452–8.

13. Nieman DC, Henson DA, Sha W. Ingestion of micronutrient fortified breakfast cereal has no influence on immune function in healthy children: A randomized controlled trial. Nutrition Journal [Internet]. 2011;10. Available from: http://www.embase.com/search/results?subaction=viewrecord&from=export&id=L51384261

14. De Gier B, Ponce MC, Perignon M, Fiorentino M, Khov K, Chamnan C, De Boer MR, Parker ME, Burja K, Dijkhuizen MA, et al. Micronutrient-fortified rice can increase hookworm infection risk: A cluster randomized trial. PLoS ONE [Internet]. Public Library of Science; 2016;11. Available from: https://www.scopus.com/inward/record.uri?eid=2-s2.0-84954091365&doi=10.1371%2fjournal.pone.0145351&partnerID=40&md5=82b0f1bd54675139a3da585d1a273698

15. Ara G, Khanam M, Rahman AS, Islam Z, Farhad S, Sanin KI, Khan SS, Rahman MM, Majoor H, Ahmed T. Effectiveness of micronutrient-fortified rice consumption on anaemia and zinc status among vulnerable women in Bangladesh. PLoS ONE [Internet]. Public Library of Science; 2019;14. Available from: https://www.scopus.com/inward/record.uri?eid=2-s2.0-85059829140&doi=10.1371%2fjournal.pone.0210501&partnerID=40&md5=f0a7361196c7c59ee37c587f6f6665fc

16. Engle-Stone R, Nankap M, Ndjebayi AO, Allen LH, Shahab-Ferdows S, Hampel D, Killilea DW, Gimou MM, Houghton LA, Friedman A, et al. Iron, Zinc, Folate, and Vitamin B-12 Status Increased among Women and Children in Yaounde and Douala, Cameroon, 1 Year after Introducing Fortified Wheat Flour. Journal of Nutrition. 2017;147:1426–36.

1. Abbreviations: C, control; I, intervention; LRTI, lower respiratory tract infection; MMN, multiple micronutrient; NA, not applicable; NM, not measured; NR, not reported; NS, not significant; NR, NutriRice; PSAC, pre-school aged children; URN, UltraRice New; URO, UltraRice Original; URTI, upper respiratory tract infection; WRA, women of reproductive age

   **P*<0.05

   ***P* <0.01

   Sample size included in analysis [↑](#endnote-ref-1)
2. Population characteristics included are age and health status. Age is a range, unless footnoted otherwise. [↑](#endnote-ref-2)
3. Except where specified, the control was the same food but non-fortified (with any micronutrients) [↑](#endnote-ref-3)
4. If unit is expressed as mg/kg, the value is referring to fortification level and it was not possible to calculate dose if intake was not provided. If unit is expressed as mg/day, the value is referring to dose [↑](#endnote-ref-4)
5. Durations were converted to months using the following methodology: 4 weeks=1 month, 30 days=1 month, 1 year=12 months [↑](#endnote-ref-5)
6. Mean ± standard deviation [↑](#endnote-ref-6)
7. Error not specified [↑](#endnote-ref-7)
8. Study authors labeled this estimate as an OR. However, the review authors believe it is a rate ratio, as it is calculated based on number of days followed-up. [↑](#endnote-ref-8)
9. Define other [↑](#endnote-ref-9)
10. Define other [↑](#endnote-ref-10)
11. Calculated by review authors [↑](#endnote-ref-11)
