# Supplementary material for: Effects of Foods Fortified with Zinc, Alone or Cofortified with Multiple Micronutrients, on Health and Functional Outcomes: A Systematic Review and Meta-Analysis
Source: Adv Nutr. 2021 Jun 24;12(5):1821–37. doi: 10.1093/advances/nmab065 (PMC8483949; doi:10.1093/advances/nmab065)
Supplement: nmab065_Supplemental_Files [file nmab065_supplemental_files.zip › Supplemental Tables 2-5.Risk of bias scores.docx]

Table S2: Risk of Bias quality scores for randomized, controlled studies assessing foods fortified with zinc, alone or co-fortified with multiple micronutrients*

| *Reference* | *Quality score* | *If "poor", state reason* |
| --- | --- | --- |
| Aaron 2011a (1) | Good |  |
| Aaron 2011b (2) | Good |  |
| Angeles-Agdeppa 2017 (3) | Good |  |
| Dhingra 2004 (4) Marwah 2004 (5) Sazawal 2007 (6) Sazawal 2010 (7) | Good |  |
| Hieu 2012 (8) | Good |  |
| Muthayya 2009 (9) Thomas 2012 (10) | Good |  |
| Nga 2009 (11) Nga 2011 (12) | Good |  |
| Petrova 2019 (13) | Good |  |
| Pinkaew 2013 (14) | Good |  |
| RameshwarSarma 2006 (15)  Sivakumar 2006 (16)  Vazir 2006 (17) | Good |  |
| Sazawal 2013 (18) | Good |  |
| Thankachan 2013 (19) | Good |  |
| Villalpando 2006 (20) | Good |  |
| Manger 2008 (21) Winichagoon 2006 (22) | Good |  |
| Abrams 2003 (23) | Fair |  |
| Bardosono 2009 (24) | Fair |  |
| Chen 2008 (25) Chen 2011 (26) | Fair |  |
| Kiliç 1998 (27) Saldamli 1996 (28) | Fair |  |
| Hettiarachchi 2004 (29) | Fair |  |
| Nieman 2011 (30) | Fair |  |
| Vinodkumar 2009 (31) | Fair |  |
| Hyder 2007 (32) | Fair |  |
| Angeles-Agdeppa 2011 (33) | Poor | Baseline zinc across groups was different; differential drop out; fingerprick blood |
| Badii 2012 (34) | Poor | Insufficient reported details on blinding, baseline characteristics, and adequate sample size necessary |
| Costarelli 2014 (35) | Poor | Baseline statistics not reported, not randomized |
| Do 2009 (36) | Poor | Insufficiently reported details on blinding |
| Haibin 2001 (37) | Poor | Insufficient reported details on blinding, adherence, serum/plasma zinc collection, high dropout rate (overall and between groups) |
| Ibnu 2019 (38) | Poor | Insufficient reported details on serum/plasma zinc collection, high dropout rate |
| Méndez 2012 (39) | Poor | Insufficient reported details on blinding, high dropout rate |
| Sari 2014 (40) | Poor | Insufficient reported details on blinding, baseline characteristics, and dropout rate (overall and between groups); difference in adherence across groups |
| Trinidad 2015 (41) | Poor | Not blinded; insufficient reported details on baseline characteristics |
| Wibowo 2016 (42) | Poor | High dropout, lack randomization/blinding details |
| *Tool: National Heart, Lung, and Blood Institute (NHLBI) Quality Assessment of Controlled Intervention Studies https://www.nhlbi.nih.gov/health-topics/study-quality-assessment-tools | | |

Table S3: Risk of Bias quality scores for controlled effectiveness assessing foods fortified with zinc, alone or co-fortified with multiple micronutrients*

| *Reference* | *Quality score* | *If "poor", state reason* |
| --- | --- | --- |
| De Gier 2016 (43) Fiorentino 2018 (44) Kuong 2019 (45) | Good |  |
| Ara 2019 (46) | Fair |  |
| Della Lucia 2016 (47) DellaLucia 2017 (48) | Fair |  |
| Dutta 2019 (49) | Fair |  |
| Hambidge 1979 (50) | Fair |  |
| Huo 2011 (51) | Fair |  |
| Huo 2012 (52) | Fair |  |
| Jiang 2011 (53) | Poor | Insufficiently reported details on study population, recruitment, sample size estimation, blinding, loss to follow-up |
| Ohiokepehai (54) | Poor | Insufficiently or unclear reported details on study design, recruitment, sample size estimation, blinding, loss to follow-up |
| *Tool: National Heart, Lung, and Blood Institute (NHLBI) Quality Assessment of Observational Cohort and Cross-Sectional Studies  <https://www.nhlbi.nih.gov/health-topics/study-quality-assessment-tools> | | |

Table S4: Risk of Bias quality scores for non-controlled effectiveness assessing foods fortified with zinc, alone or co-fortified with multiple micronutrients*

| *Reference* | *Quality score* | *If "poor", state reason* |
| --- | --- | --- |
| Engle-Stone 2017 (55) | Good |  |
| Stuetz 2012 (56) | Fair |  |
| Sun 2013 (57) | Fair |  |
| Varea 2011 (58) | Fair |  |
| Varea 2012 (59) | Fair |  |
| Méndez 2014 (60) | Fair |  |
| Tukvadze 2013 (61) | Poor | Insufficiently reported details on sample size estimation, fortification intervention, blinding, statistical testing, serum/plasma zinc assessment methodology |
| *Tool: National Heart, Lung, and Blood Institute (NHLBI) Quality Assessment Tool for Before-After (Pre-Post) Studies With No Control Group  <https://www.nhlbi.nih.gov/health-topics/study-quality-assessment-tools> | | |

Table S5: Risk of Bias quality scores for zinc absorption studies assessing foods fortified with zinc, alone or co-fortified with multiple micronutrients*

| *Reference* | *Quality score* | *If "poor", state reason* |
| --- | --- | --- |
| Hansen 2001 (62) | Good |  |
| López de Romaña 2003 (63) | Fair |  |
| Méndez 2015 (64) | Fair |  |
| Rosado 2012 (65) | Fair |  |
| Ruz 2005 (66) | Fair |  |
| Sandström 1980 (67) | Fair |  |
| *Tool: Modified version of the National Heart, Lung, and Blood Institute (NHLBI) Quality Assessment of Controlled Intervention Studies <https://www.nhlbi.nih.gov/health-topics/study-quality-assessment-tools> | | |

1. Aaron GJ, Kariger P, Aliyu R, Flach M, Iya D, Obadiah M, et al. A Multi-Micronutrient Beverage Enhances the Vitamin A and Zinc Status of Nigerian Primary Schoolchildren. Journal of Nutrition. 2011;141(8):1565–72.

2. Aaron GJ, Ba Lo N, Hess SY, Guiro AT, Wade S, Brown KH. Plasma Zinc Concentration Increases within 2 Weeks in Healthy Senegalese Men Given Liquid Supplemental Zinc, but Not Zinc-Fortified Wheat Bread. The Journal of Nutrition. 2011 Jul 1;141(7):1369–74.

3. Angeles-Agdeppa I, Magsadia CR, Aaron GJ, Lloyd BB, Hilmers DC, Bhutta ZA. A Micronutrient Fortified Beverage Given at Different Dosing Frequencies Had Limited Impact on Anemia and Micronutrient Status in Filipino Schoolchildren. Nutrients [Internet]. 2017;9(9). Available from: ://WOS:000411973200085

4. Dhingra P, Menon VP, Sazawal S, Dhingra U, Marwah D, Sarkar A, et al. Effect of fortification of milk with zinc and iron along with vitamins C, E, A and selenium on growth, iron status and development in preschool children - A community based double-masked randomized trial [Internet]. 2004. 53 p. Available from: ://WOS:000227354700008

5. Marwah D, Sazawal S, Dhingra U, Verma P, Deb S, Dhingra P, et al. Efficacy of micronutrient fortification of milk in prevention of childhood morbidity in children 1-3 years of age - A community based double masked randomized trial [Internet]. 2004. 367 p. Available from: ://WOS:000227354700062

6. Sazawal S, Dhingra U, Dhingra P, Hiremath G, Kumar J, Sarkar A, et al. Effects of fortified milk on morbidity in young children in north India: community based, randomised, double masked placebo controlled trial. Bmj. 2007;334(7585):140.

7. Sazawal S, Dhingra U, Dhingra P, Hiremath G, Sarkar A, Dutta A, et al. Micronutrient fortified milk improves iron status, anemia and growth among children 1-4 years: A double masked, randomized, controlled trial. PLoS ONE [Internet]. 2010;5(8). Available from: https://www.scopus.com/inward/record.uri?eid=2-s2.0-77957861492&doi=10.1371%2fjournal.pone.0012167&partnerID=40&md5=7c3940d6762d5c82fbccb481dc8cce42

8. Hieu NT, Sandalinas F, De Sesmaisons A, Laillou A, Tam NP, Khan NC, et al. Multi-micronutrient-fortified biscuits decreased the prevalence of anaemia and improved iron status, whereas weekly iron supplementation only improved iron status in Vietnamese school children. British Journal of Nutrition. 2012;108(8):1419–27.

9. Muthayya S, Eilander A, Transler C, Thomas T, van der Knaap HCM, Srinivasan K, et al. Effect of fortification with multiple micronutrients and n-3 fatty acids on growth and cognitive performance in Indian schoolchildren: the CHAMPION (Children’s Health and Mental Performance Influenced by Optimal Nutrition) Study. American Journal of Clinical Nutrition. 2009;89(6):1766–75.

10. Thomas T, Eilander A, Muthayya S, McKay S, Thankachan P, Theis W, et al. The effect of a 1-year multiple micronutrient or n-3 fatty acid fortified food intervention on morbidity in Indian school children. European Journal of Clinical Nutrition. 2012;66(4):452–8.

11. Nga TT, Wasantwisut E, Furr H, Wieringa FT, Winichagoon P, Dijkhuizen MA, et al. Multi-Micronutrient-Fortified Biscuits Decreased Prevalence of Anemia and Improved Micronutrient Status and Effectiveness of Deworming in Rural Vietnamese School Children. Journal of Nutrition. 2009;139(5):1013–21.

12. Nga TT, Winichagoon P, Dijkhuizen MA, Khan NC, Wasantwisut E, Wieringa FT. Decreased Parasite Load and Improved Cognitive Outcomes Caused by Deworming and Consumption of Multi-Micronutrient Fortified Biscuits in Rural Vietnamese Schoolchildren. American Journal of Tropical Medicine and Hygiene. 2011;85(2):333–40.

13. Petrova D, Bernabeu Litrán MA, García-Mármol E, Rodríguez-Rodríguez M, Cueto-Martín B, López-Huertas E, et al. Еffects of fortified milk on cognitive abilities in school-aged children: results from a randomized-controlled trial. European Journal of Nutrition. 2019;58(5):1863–72.

14. Pinkaew S, Winichagoon P, Hurrell RF, Wegmuller R. Extruded Rice Grains Fortified with Zinc, Iron, and Vitamin A Increase Zinc Status of Thai School Children When Incorporated into a School Lunch Program. Journal of Nutrition. 2013;143(3):362–8.

15. Rameshwar Sarma KV, Udaykumar P, Balakrishna N, Vijayaraghavan K, Sivakumar B. Effect of micronutrient supplementation on health and nutritional status of schoolchildren: Growth and morbidity. Nutrition. 2006;22(1 SUPPL.):S8–14.

16. Sivakumar B, Nair KM, Sreeramulu D, Suryanarayana P, Ravinder P, Shatrugna V, et al. Effect of micronutrient supplement on health and nutritional status of schoolchildren: biochemical status. Nutrition. 2006 Jan;22(1 Suppl):S15-25.

17. Vazir S, Nagalla B, Thangiah V, Kamasamudram V, Bhattiprolu S. Effect of micronutrient supplement on health and nutritional status of schoolchildren: mental function. Nutrition. 2006 Jan;22(1 Suppl):S26-32.

18. Sazawal S, Habib A, Dhingra U, Dutta A, Dhingra P, Sarkar A, et al. Impact of micronutrient fortification of yoghurt on micronutrient status markers and growth - a randomized double blind controlled trial among school children in Bangladesh. BMC Public Health. 2013;13:514.

19. Thankachan P, Selvam S, Surendran D, Chellan S, Pauline M, Abrams SA, et al. Efficacy of a multi micronutrient-fortified drink in improving iron and micronutrient status among schoolchildren with low iron stores in India: A randomised, double-masked placebo-controlled trial. European Journal of Clinical Nutrition. 2013;67(1):36–41.

20. Villalpando S, Shamah T, Rivera JA, Lara Y, Monterrubio E. Fortifying milk with ferrous gluconate and zinc oxide in a public nutrition program reduced the prevalence of anemia in toddlers. Journal of Nutrition. 2006;136(10):2633–7.

21. Manger MS, McKenzie JE, Winichagoon P, Gray A, Chavasit V, Pongcharoen T, et al. A micronutrient-fortified seasoning powder reduces morbidity and improves short-term cognitive function, but has no effect on anthropometric measures in primary school children in northeast Thailand: A randomized controlled trial. American Journal of Clinical Nutrition. 2008;87(6):1715–22.

22. Winichagoon P, Bailey KB, Manger MS, Gibson RS, Wasantwisut E, Boonpraderm A, et al. A Multimicronutrient-Fortified Seasoning Powder Enhances the Hemoglobin, Zinc, and Iodine Status of Primary School Children in North East Thailand: A Randomized Controlled Trial of Efficacy. Journal of Nutrition. 2006;136(6):1617–23.

23. Abrams SA, Mushi A, Hilmers DC, Griffin IJ, Davila P, Allen L. A multinutrient-fortified beverage enhances the nutritional status of children in Botswana. Journal of Nutrition. 2003;133(6):1834–40.

24. Bardosono S, Dewi LE, Sukmaniah S, Permadhi I, Eka AD, Lestarina L. Effect of a six-month iron-zinc fortified milk supplementation on nutritional status, physical capacity and speed learning process in Indonesian underweight schoolchildren: Randomized, placebo-controlled. Medical Journal of Indonesia. 2009;18(3):193–202.

25. Chen K, Li TY, Chen L, Qu P, Liu YX. Effects of vitamin A, vitamin A plus iron and multiple micronutrient-fortified seasoning powder on preschool children in a suburb of Chongqing, China. Journal of Nutritional Science and Vitaminology. 2008;54(6):440–7.

26. Chen K, Wei X, Qu P, Liu Y, Zhang X, Li T, et al. Effect of vitamin A, vitamin A plus iron and multiple micronutrient-fortified seasoning powder on infectious morbidity of preschool children [electronic resource]. Nutrition. 2011;27(4):428–34.

27. Kiliç I, Ozalp I, Coskun T, Tokatli A, Emre S, Saldamli I, et al. The effect of zinc-supplemented bread consumption on school children with asymptomatic zinc deficiency. J Pediatr Gastroenterol Nutr. 1998;26(2):167–71.

28. Saldamli I, Ozalp I, Kilic I, Koksel H, Ozboy O. Zinc-supplemented bread and its utilization in zinc deficiency. Cereal Chemistry. 1996;73(4):424–7.

29. Hettiarachchi M, Hilmers DC, Liyanage C, Abrams SA. Na2EDTA Enhances the Absorption of Iron and Zinc from Fortified Rice Flour in Sri Lankan Children. The Journal of Nutrition. 2004 Nov 1;134(11):3031–6.

30. Nieman DC, Henson DA, Sha W. Ingestion of micronutrient fortified breakfast cereal has no influence on immune function in healthy children: A randomized controlled trial. Nutrition Journal [Internet]. 2011;10(1). Available from: http://www.embase.com/search/results?subaction=viewrecord&from=export&id=L51384261

31. Vinodkumar M, Erhardt JG, Rajagopalan S. Impact of a multiple-micronutrient fortified salt on the nutritional status and memory of schoolchildren. International Journal for Vitamin and Nutrition Research. 2009;79(5–6):348–61.

32. Ziauddin Hyder SM, L©œnnerdal B, Rahman M, Mehansho H, Mannar V, Khan M, et al. A Multiple-Micronutrient-Fortified Beverage Affects Hemoglobin, Iron, and Vitamin A Status and Growth in Adolescent Girls in Rural Bangladesh. Journal of Nutrition. 2007;137(9):2147–53.

33. Angeles-Agdeppa I, Magsadia CR, Capanzana MV. Fortified juice drink improved iron and zinc status of schoolchildren. Asia Pacific Journal of Clinical Nutrition. 2011;20(4):535–43.

34. Badii A, Nekouei N, Fazilati M, Shahedi M, Badiei S. Effect of Consuming Zinc-fortified Bread on Serum Zinc and Iron Status of Zinc-deficient Women: A Double Blind, Randomized Clinical Trial. Int J Prev Med. 2012 Mar;3(Suppl 1):S124-130.

35. Costarelli L, Giacconi R, Malavolta M, Basso A, Piacenza F, DeMartiis M, et al. Effects of zinc-fortified drinking skim milk (as functional food) on cytokine release and thymic hormone activity in very old persons: A pilot study. Age. 2014;36(3):1421–31.

36. Do TKL, Bui TN, Nguyen CK, Le TH, Nguyen TQN, Nguyen TH, et al. Impact of milk consumption on performance and health of primary school children in rural Vietnam. Asia Pacific Journal of Clinical Nutrition. 2009;18(3):326–34.

37. Haibin A, Yin S, Xu Q. [Effects of supplementing calcium, iron and zinc on the fetus development and growth during pregnancy]. Zhonghua Yu Fang Yi Xue Za Zhi. 2001 Nov;35(6):370–3.

38. Ibnu IN, Thaha RM, Suriah. Effect of iron and zinc substance giving through fortification rice on stress level of school age children in islamic boarding school annihayahkarawang. Indian Journal of Public Health Research and Development. 2019;10(4):1071–7.

39. Méndez RO, Galdamez K, Grijalva MI, Quihui L, Garcia HS, de la Barca AM. Effect of micronutrient-fortified milk on zinc intake and plasma concentration in adolescent girls. J Am Coll Nutr. 2012;31(6):408–14.

40. Sari DK, Marliyati SA, Kustiyah L, Khomsan A. Role of biscuits enriched with albumin protein from snakehead fish, zinc and iron on immune response of under five children. Pakistan Journal of Nutrition. 2014;13(1):28–32.

41. Trinidad TP, Mallillin AC, Sagum RS, de Leon MP, Borlagdan MS, Baquiran AFP. Fortified milk consumption among 6-year old children: changes in biochemical markers of trace minerals and vitamins. Trace Elements and Electrolytes. 2015;32(3):112–8.

42. Wibowo N, Bardosono S, Irwinda R. Effects of Bifidobacterium animalis lactis HN019 (DR10TM), inulin, and micronutrient fortified milk on faecal DR10TM, immune markers, and maternal micronutrients among Indonesian pregnant women. Asia Pacific Journal of Clinical Nutrition. 2016;25:S102–10.

43. De Gier B, Ponce MC, Perignon M, Fiorentino M, Khov K, Chamnan C, et al. Micronutrient-fortified rice can increase hookworm infection risk: A cluster randomized trial. PLoS ONE [Internet]. 2016;11(1). Available from: https://www.scopus.com/inward/record.uri?eid=2-s2.0-84954091365&doi=10.1371%2fjournal.pone.0145351&partnerID=40&md5=82b0f1bd54675139a3da585d1a273698

44. Fiorentino M, Perignon M, Kuong K, de Groot R, Parker M, Burja K, et al. Effect of multi-micronutrient-fortified rice on cognitive performance depends on premix composition and cognitive function tested: results of an effectiveness study in Cambodian schoolchildren. Public Health Nutrition. 2018;21(4):816–27.

45. Kuong K, Tor P, Perignon M, Fiorentino M, Chamnan C, Berger J, et al. Multi-Micronutrient Fortified Rice Improved Serum Zinc and Folate Concentrations of Cambodian School Children. A Double-Blinded Cluster-Randomized Controlled Trial. Nutrients. 2019 Nov 20;11(12).

46. Ara G, Khanam M, Rahman AS, Islam Z, Farhad S, Sanin KI, et al. Effectiveness of micronutrient-fortified rice consumption on anaemia and zinc status among vulnerable women in Bangladesh. PLoS ONE [Internet]. 2019;14(1). Available from: https://www.scopus.com/inward/record.uri?eid=2-s2.0-85059829140&doi=10.1371%2fjournal.pone.0210501&partnerID=40&md5=f0a7361196c7c59ee37c587f6f6665fc

47. Della Lucia CM, Rodrigues KC, Rodrigues VC, Santos LL, Cardoso LM, Martino HS, et al. Diet Quality and Adequacy of Nutrients in Preschool Children: Should Rice Fortified with Micronutrients Be Included in School Meals? Nutrients. 2016;8(5).

48. Della Lucia CM, Santos LLM, Da Silva BP, Anunciação PC, Alfenas RCG, Franceschini SCC, et al. Impact of rice fortified with iron, zinc, thiamine and folic acid on laboratory measurements of nutritional status of preschool children. Ciencia e Saude Coletiva. 2017;22(2):583–92.

49. Dutta A. Assessment of fortification of Mid-Day Meal Programme in Dhenkanal, Odisha, 2016-2018 Evaluation Report - India [Internet]. World Food Programme; 2020 [cited 2020 Jul 7]. Available from: https://reliefweb.int/report/india/assessment-fortification-mid-day-meal-programme-dhenkanal-odisha-2016-2018-evaluation

50. Hambidge KM, Chavez MN, Brown RM, Walravens PA. ZINC NUTRITIONAL-STATUS OF YOUNG MIDDLE-INCOME CHILDREN AND EFFECTS OF CONSUMING ZINC-FORTIFIED BREAKFAST CEREALS. American Journal of Clinical Nutrition. 1979;32(12):2532–9.

51. Huo J, Sun J, Huang J, Li W, Wang L, Selenje L, et al. The effectiveness of fortified flour on micro-nutrient status in rural female adults in China. Asia Pacific Journal of Clinical Nutrition. 2011;20(1):118–24.

52. Huo J, Gary RG, Jian H, Jing S, Lijuan W, Lilian S, et al. Effectiveness of Fortified Flour for Enhancement of Vitamin and Mineral Intakes and Nutrition Status in Northwest Chinese Villages. Food and Nutrition Bulletin. 2012;33(2):161–8.

53. Jiang Z, Liang Q, Wang Y, Zheng X, Pei L, Zhang T, et al. Metabonomic study on women of reproductive age treated with nutritional intervention: Screening potential biomarkers related to neural tube defects occurrence. Biomedical Chromatography. 2011;25(7):767–74.

54. Ohiokpehai O, David DM, Kamau J. Serum zinc levels of school children on a corn-soy blend feeding trial in primary schools in Suba district, Kenya. Journal of Applied Biosciences. 2009 May 8;17:904–12.

55. Engle-Stone R, Nankap M, Ndjebayi AO, Allen LH, Shahab-Ferdows S, Hampel D, et al. Iron, Zinc, Folate, and Vitamin B-12 Status Increased among Women and Children in Yaounde and Douala, Cameroon, 1 Year after Introducing Fortified Wheat Flour. Journal of Nutrition. 2017;147(7):1426–36.

56. Stuetz W, Carrara VI, McGready R, Lee SJ, Erhardt JG, Breuer J, et al. Micronutrient status in lactating mothers before and after introduction of fortified flour: Cross-sectional surveys in Maela refugee camp. European Journal of Nutrition. 2012;51(4):425–34.

57. Sun J, Huang J, Huo JS. School food fortification improves nutrition status of students from poor migrant families. Annals of Nutrition and Metabolism. 2013;63:483.

58. Varea A, Malpeli A, Etchegoyen G, Vojkovic M, Disalvo L, Apezteguia M, et al. Short-Term Evaluation of the Impact of a Food Program on the Micronutrient Nutritional Status of Argentinean Children Under the Age of Six. Biological Trace Element Research. 2011;143(3):1337–48.

59. Varea A, Carmuega E, Pereyras S, Etchegoyen G, Gonz©Łlez HF, Vojkovic M, et al. Evaluation of the Impact of a Food Program on the Micronutrient Nutritional Status of Argentinean Lactating Mothers [electronic resource]. Biological Trace Element Research. 2012;150(1–3):103–8.

60. Méndez RO, Santiago A, Yepiz-Plascencia G, Peregrino-Uriarte AB, Calderón de la Barca AM, García HS. Zinc fortification decreases ZIP1 gene expression of some adolescent females with appropriate plasma zinc levels. Nutrients. 2014;6(6):2229–39.

61. Tukvadze S, Kverenchkhiladze R. Inclusion of zinc fortified tea into the children’s diet and its hygienicassessment. Georgian medical news. 2013;(217):53–6.

62. Hansen M, Samman S, Madsen LT, Jensen M, Sorensen SS, Sandstrom B. Folic acid enrichment of bread does not appear to affect zinc absorption in young women. American Journal of Clinical Nutrition. 2001;74(1):125–9.

63. López de Romaña DL, Lonnerdal B, Brown KH. Absorption of zinc from wheat products fortified with iron and either zinc sulfate or zinc oxide. American Journal of Clinical Nutrition. 2003;78(2):279–83.

64. Méndez RO, Hambidge M, Baker M, Salgado SA, Ruiz J, Garcia HS, et al. Zinc Absorption from Fortified Milk Powder in Adolescent Girls. Biol Trace Elem Res. 2015;168(1):61–6.

65. Rosado JL, Diaz M, Munoz E, Westcott JL, Gonzalez KE, Krebs NF, et al. Bioavailability of zinc oxide added to corn tortilla is similar to that of zinc sulfate and is not affected by simultaneous addition of iron. Food and Nutrition Bulletin. 2012;33(4):261–6.

66. Ruz M, Codoceo J, Inostroza J, Rebolledo A, Krebs NF, Westcott JE, et al. Zinc absorption from a micronutrient-fortified dried cow’s milk used in the Chilean National Complementary Food Program. Nutrition Research. 2005;25(12):1043–8.

67. Sandstrom B, Bjorn-Rasmussen E, Cederblad A, Arvidsson B. Zinc absorption from composite meals. I. The significance of wheat extraction rate, zinc, calcium, and protein content in meals based on bread. American Journal of Clinical Nutrition. 1980;33(4):739–45.
