# Supplementary figures and images for: Effects of Foods Fortified with Zinc, Alone or Cofortified with Multiple Micronutrients, on Health and Functional Outcomes: A Systematic Review and Meta-Analysis
Source: Adv Nutr. 2021 Jun 24;12(5):1821–37. doi: 10.1093/advances/nmab065 (PMC8483949; doi:10.1093/advances/nmab065)

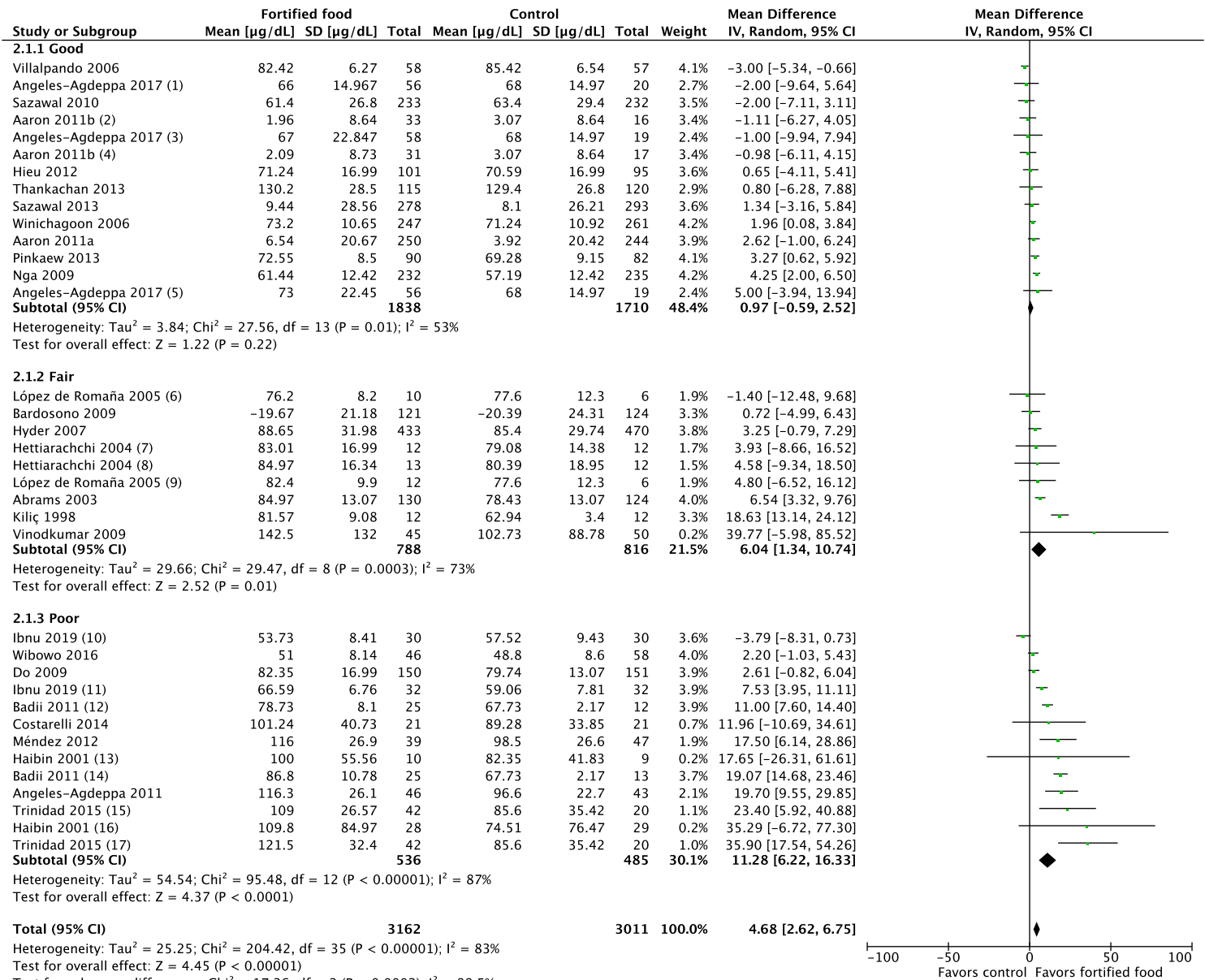

Supplement: nmab065_Supplemental_Files [file nmab065_supplemental_files.zip › Supplemental figure 1.pdf]

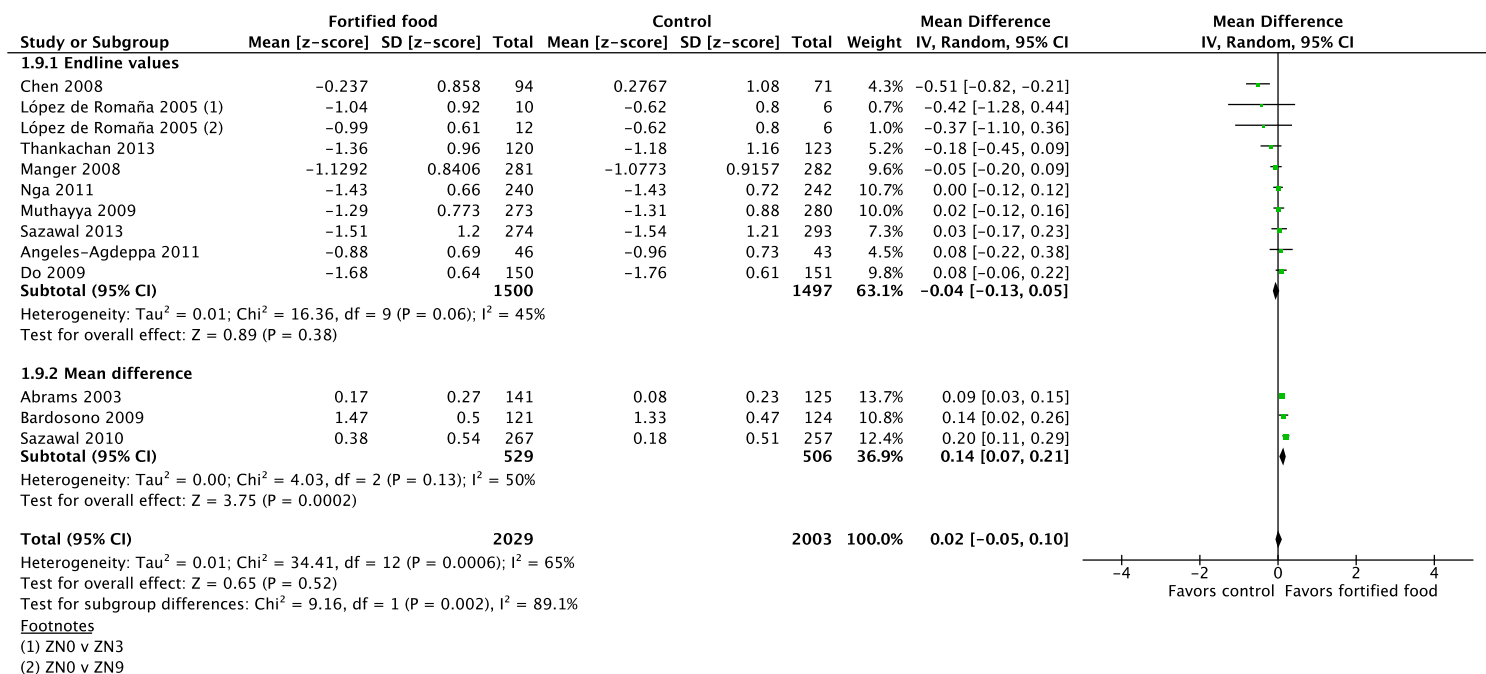

Supplement: nmab065_Supplemental_Files [file nmab065_supplemental_files.zip › Supplemental figure 10.pdf]

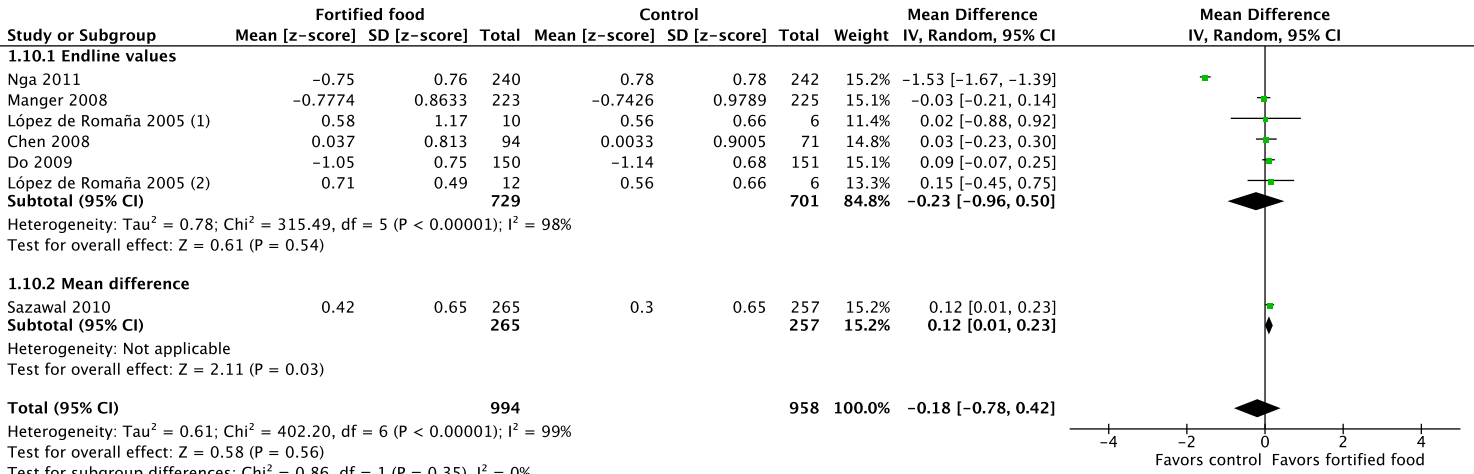

Supplement: nmab065_Supplemental_Files [file nmab065_supplemental_files.zip › Supplemental figure 11.pdf]

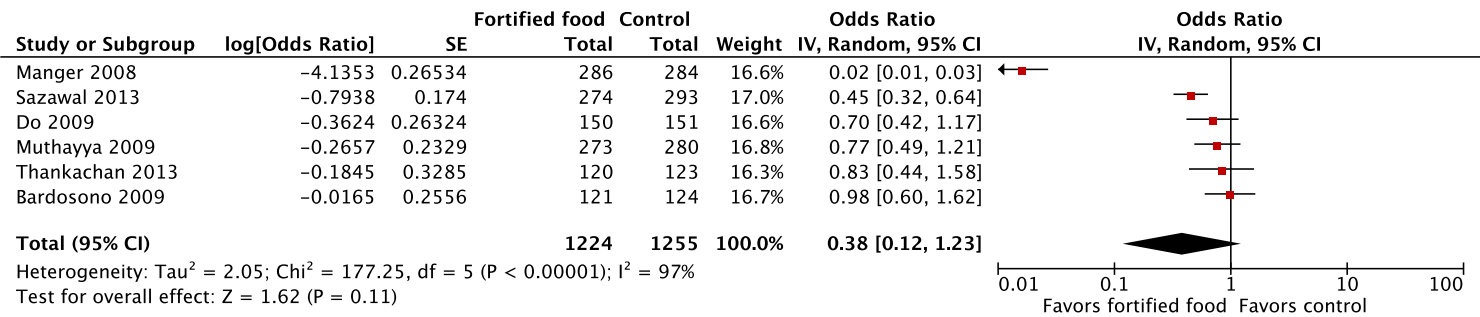

Supplement: nmab065_Supplemental_Files [file nmab065_supplemental_files.zip › Supplemental figure 12.pdf]

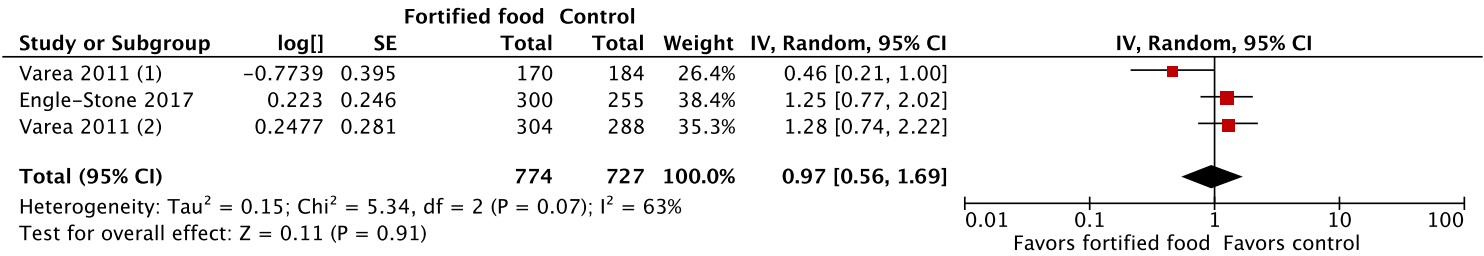

Footnotes  
(1) 1-2 years  
(2) 2-6 years

Supplement: nmab065_Supplemental_Files [file nmab065_supplemental_files.zip › Supplemental figure 13.pdf]

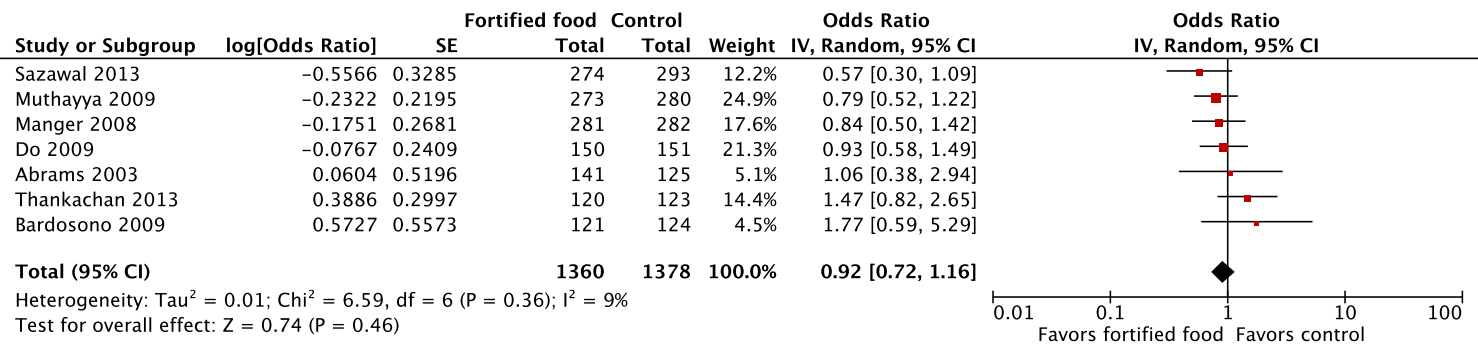

Supplement: nmab065_Supplemental_Files [file nmab065_supplemental_files.zip › Supplemental figure 14.pdf]

Supplemental figure 15.effectiveness underweight prevalence

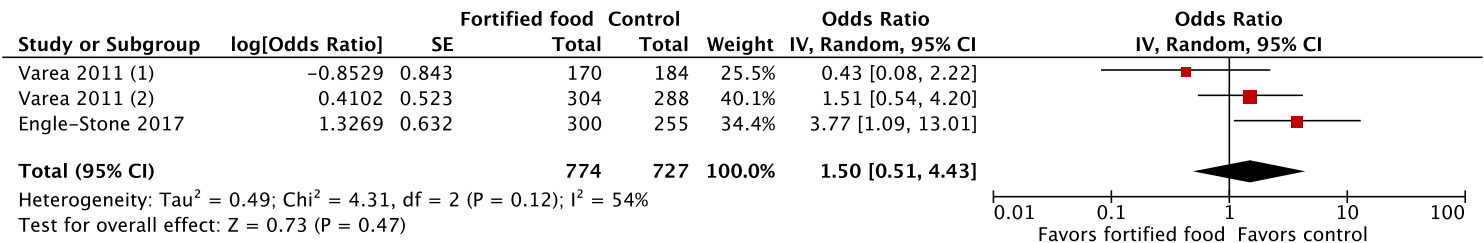

Footnotes

- (1) 1-2 years
- (2) 2-6 years

Supplement: nmab065_Supplemental_Files [file nmab065_supplemental_files.zip › Supplemental figure 15.pdf]

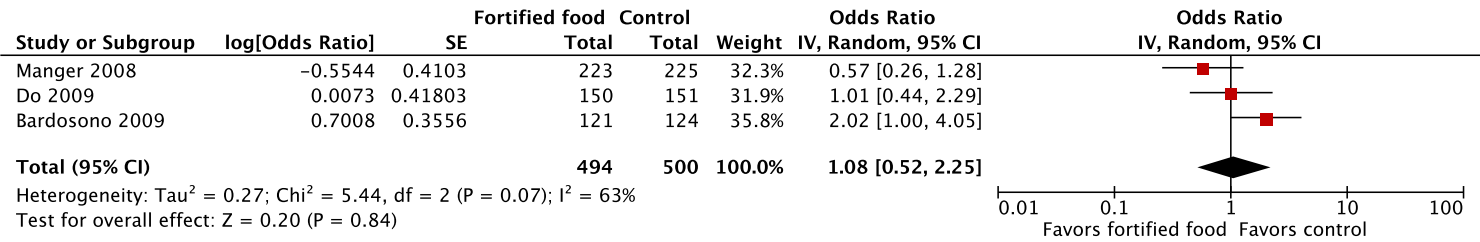

Supplement: nmab065_Supplemental_Files [file nmab065_supplemental_files.zip › Supplemental figure 16.pdf]

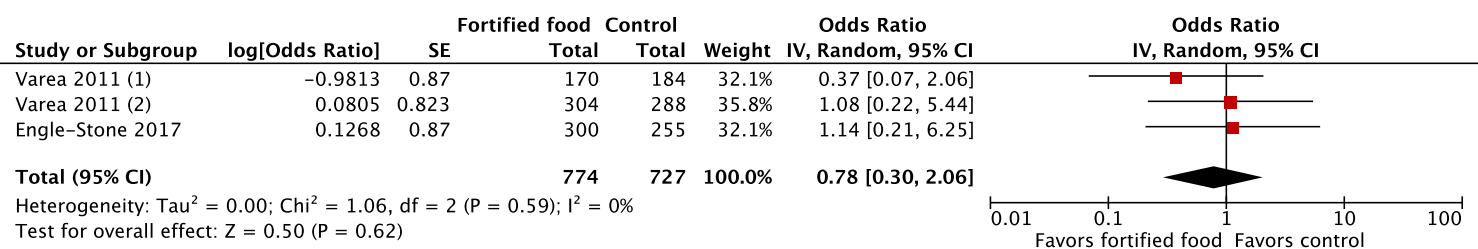

**Footnotes**  
(1) 2-6 years  
(2) 1-2 years

Supplement: nmab065_Supplemental_Files [file nmab065_supplemental_files.zip › Supplemental figure 17.pdf]

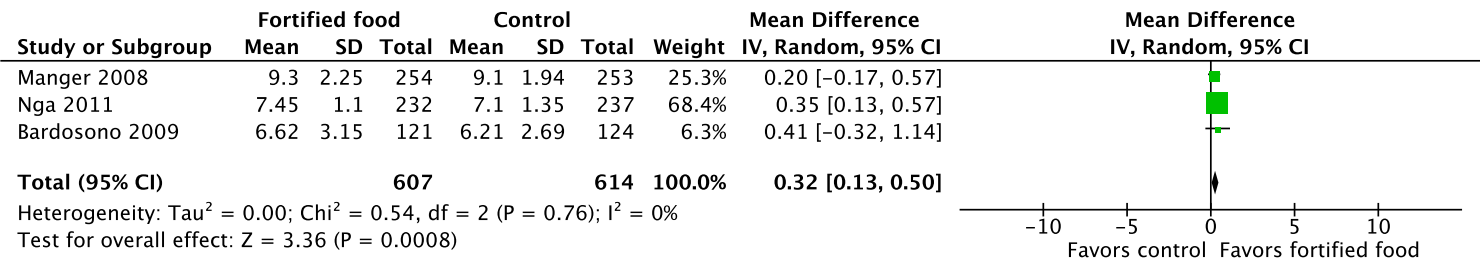

Supplement: nmab065_Supplemental_Files [file nmab065_supplemental_files.zip › Supplemental figure 19.pdf]

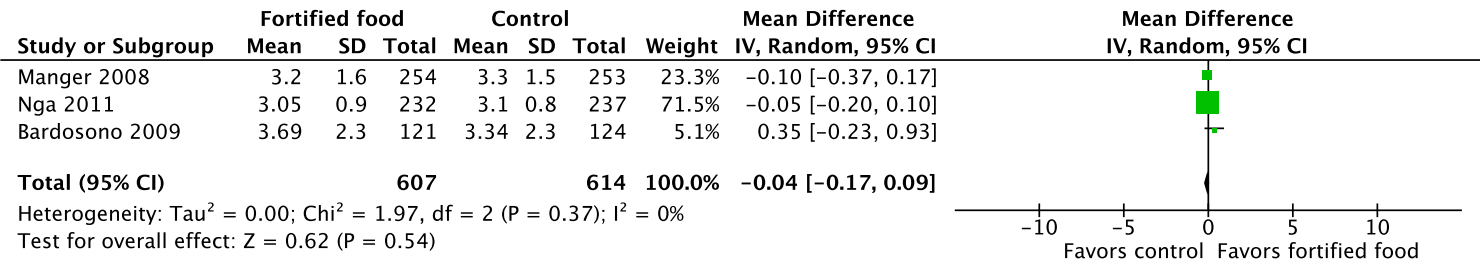

Supplement: nmab065_Supplemental_Files [file nmab065_supplemental_files.zip › Supplemental figure 20.pdf]

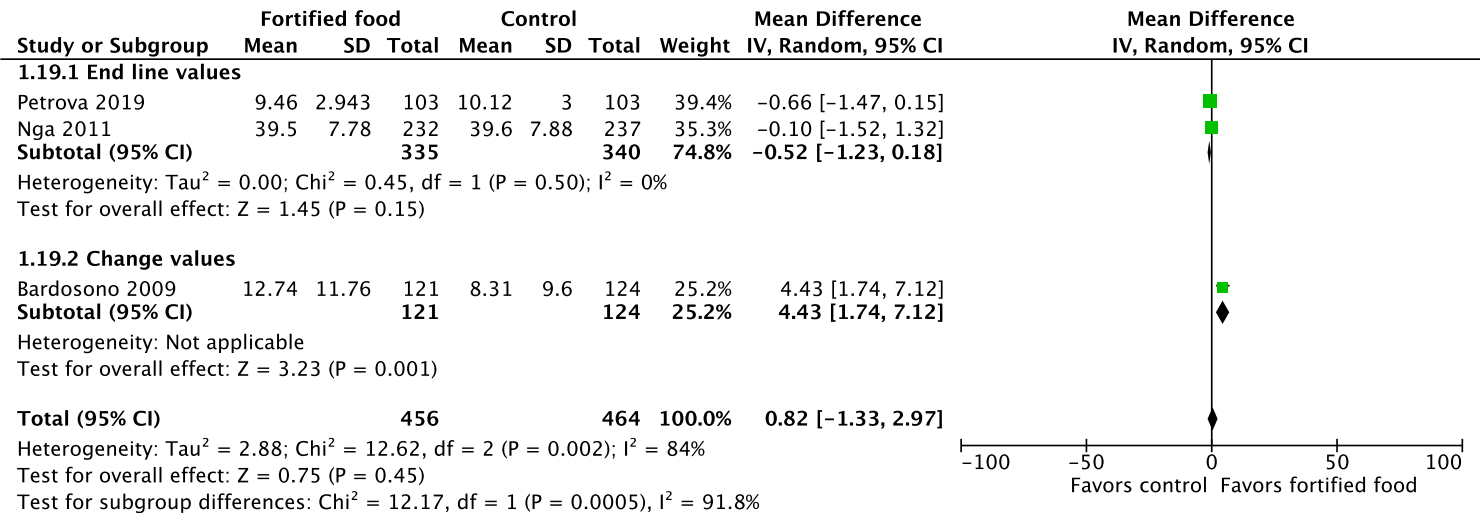

Supplement: nmab065_Supplemental_Files [file nmab065_supplemental_files.zip › Supplemental figure 21.pdf]

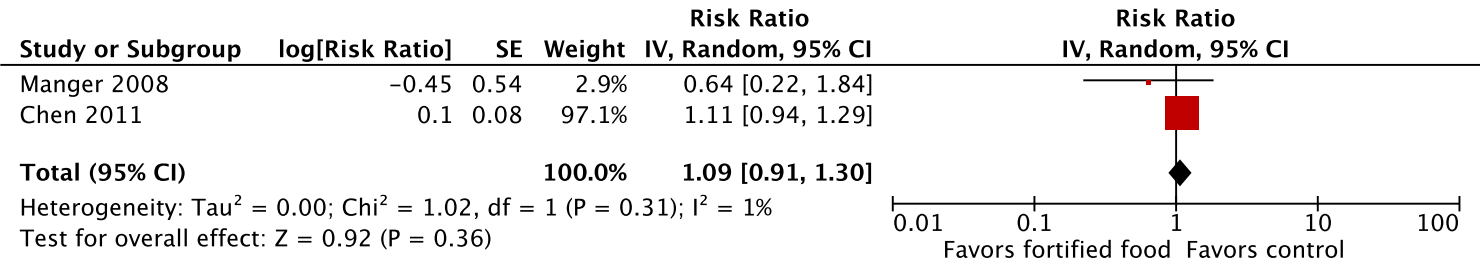

Supplement: nmab065_Supplemental_Files [file nmab065_supplemental_files.zip › Supplemental figure 22.pdf]

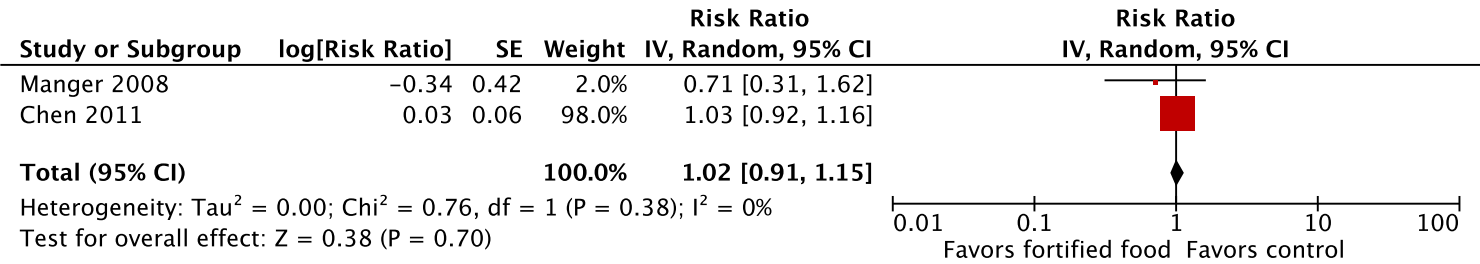

Supplement: nmab065_Supplemental_Files [file nmab065_supplemental_files.zip › Supplemental figure 23.pdf]

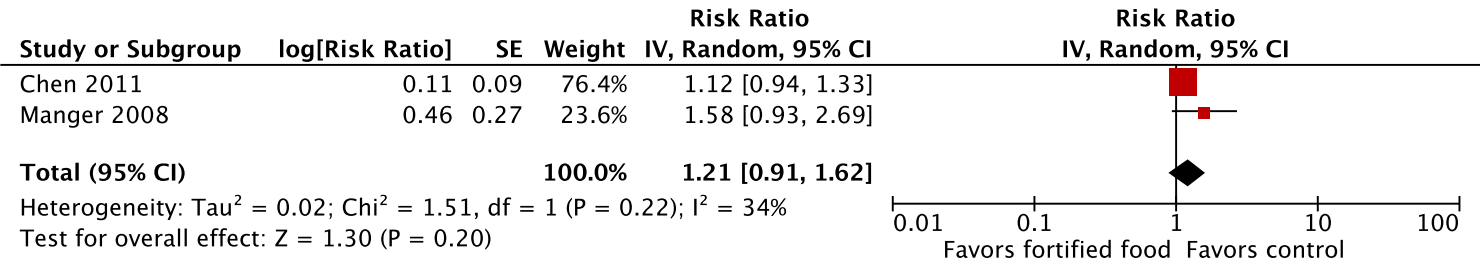

Supplement: nmab065_Supplemental_Files [file nmab065_supplemental_files.zip › Supplemental figure 24.pdf]

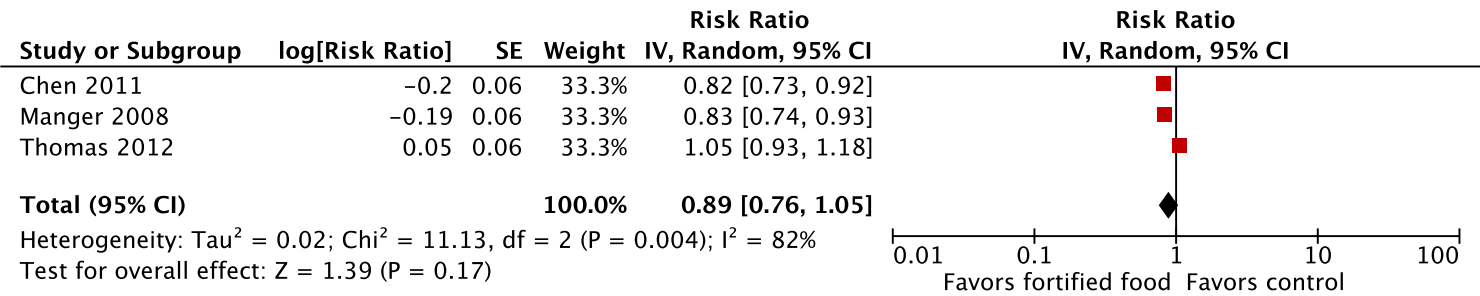

Supplement: nmab065_Supplemental_Files [file nmab065_supplemental_files.zip › Supplemental figure 25.pdf]

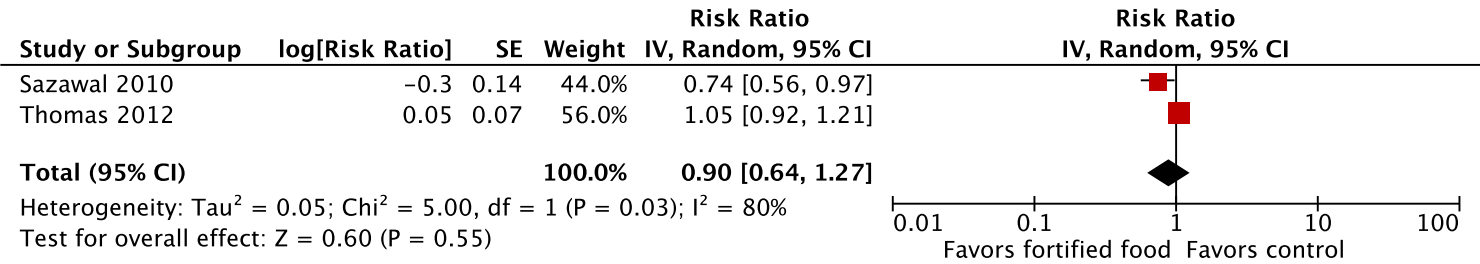

Supplement: nmab065_Supplemental_Files [file nmab065_supplemental_files.zip › Supplemental figure 26.pdf]

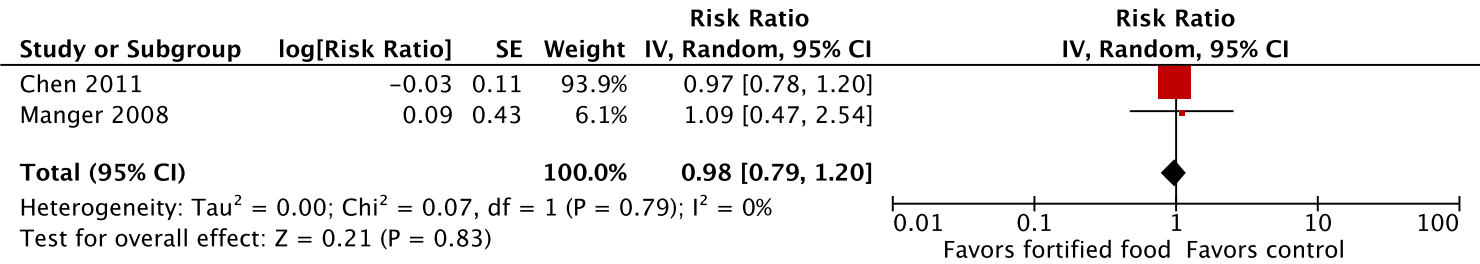

Supplement: nmab065_Supplemental_Files [file nmab065_supplemental_files.zip › Supplemental figure 27.pdf]

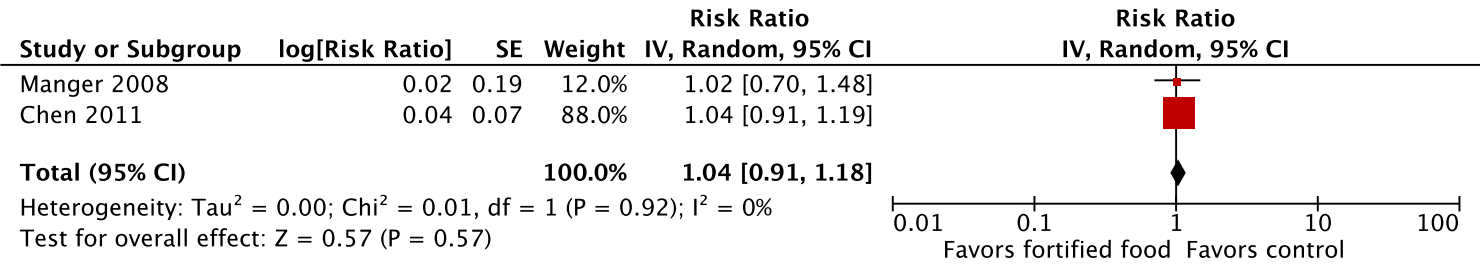

Supplement: nmab065_Supplemental_Files [file nmab065_supplemental_files.zip › Supplemental figure 28.pdf]

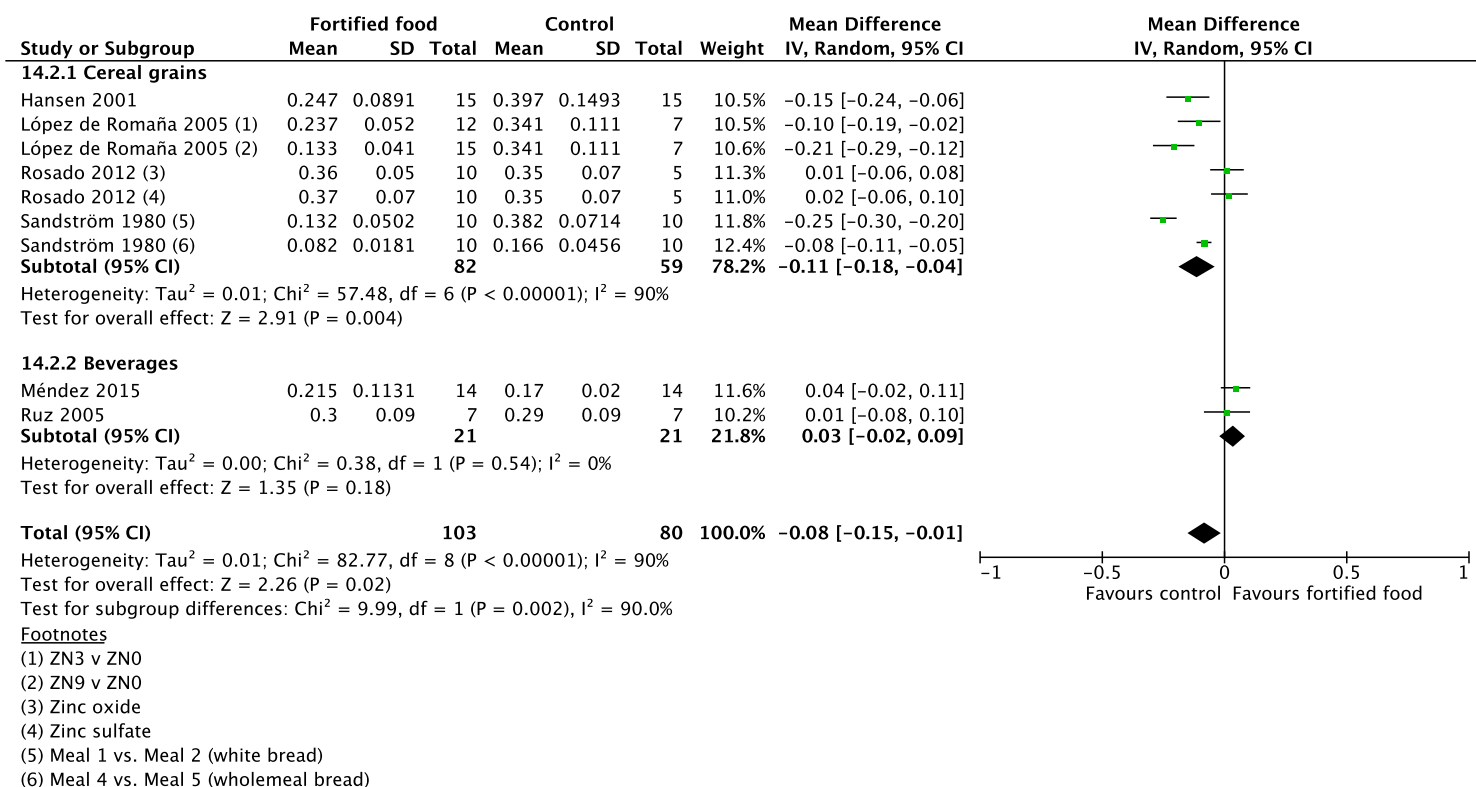

Supplement: nmab065_Supplemental_Files [file nmab065_supplemental_files.zip › Supplemental figure 31.pdf]

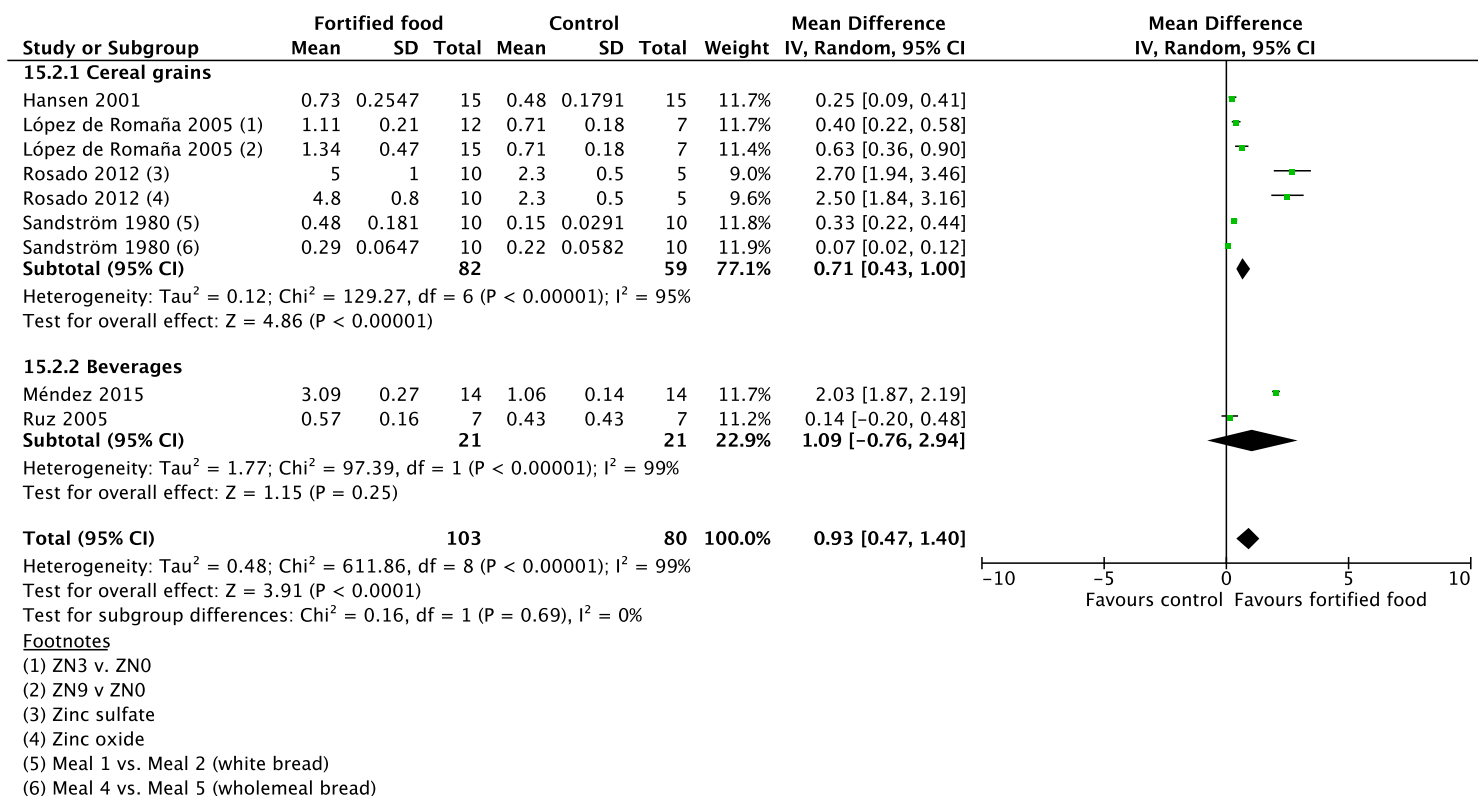

Supplement: nmab065_Supplemental_Files [file nmab065_supplemental_files.zip › Supplemental figure 34.pdf]

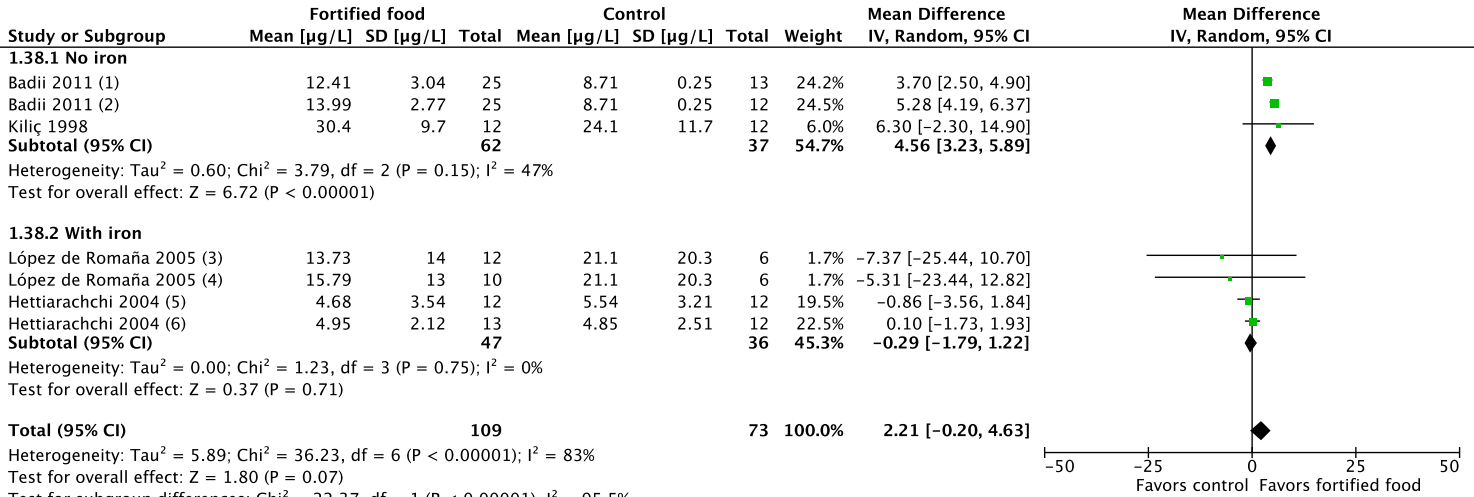

Footnotes  
(1) Control v. 10 mg/d  
(2) Control v. 5 mg/d  
(3) ZN0 v ZN9  
(4) ZN0 v ZN3  
(5) Na2EDTA+FeSO4+FA  
(6) FeSO4+FA

Supplement: nmab065_Supplemental_Files [file nmab065_supplemental_files.zip › Supplemental figure 35.pdf]

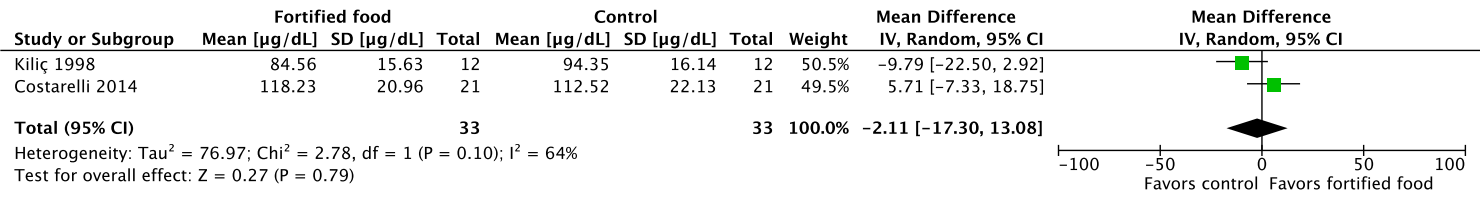

Supplement: nmab065_Supplemental_Files [file nmab065_supplemental_files.zip › Supplemental figure 36.pdf]

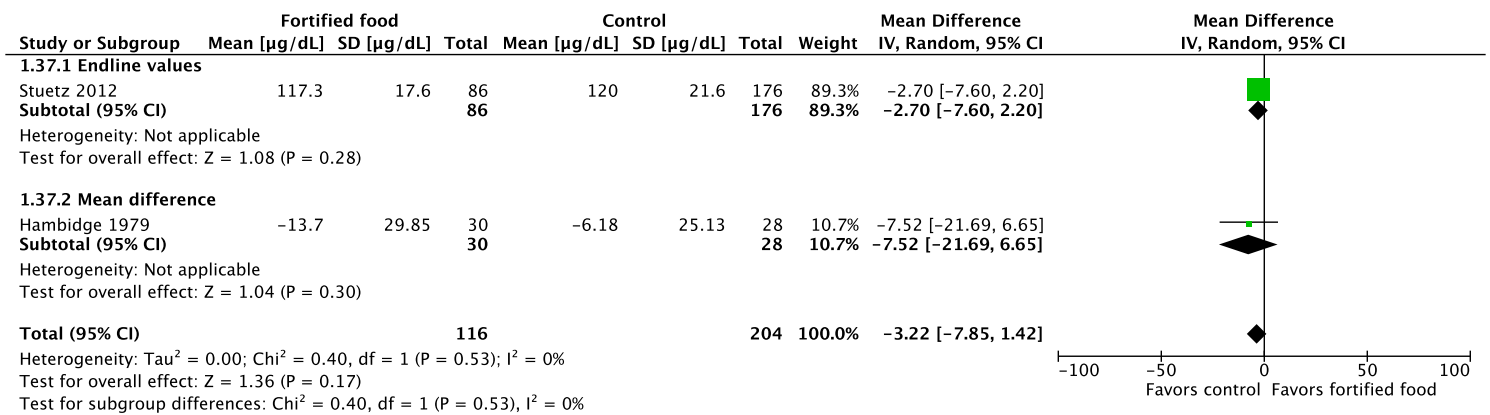

Supplement: nmab065_Supplemental_Files [file nmab065_supplemental_files.zip › Supplemental figure 37.pdf]

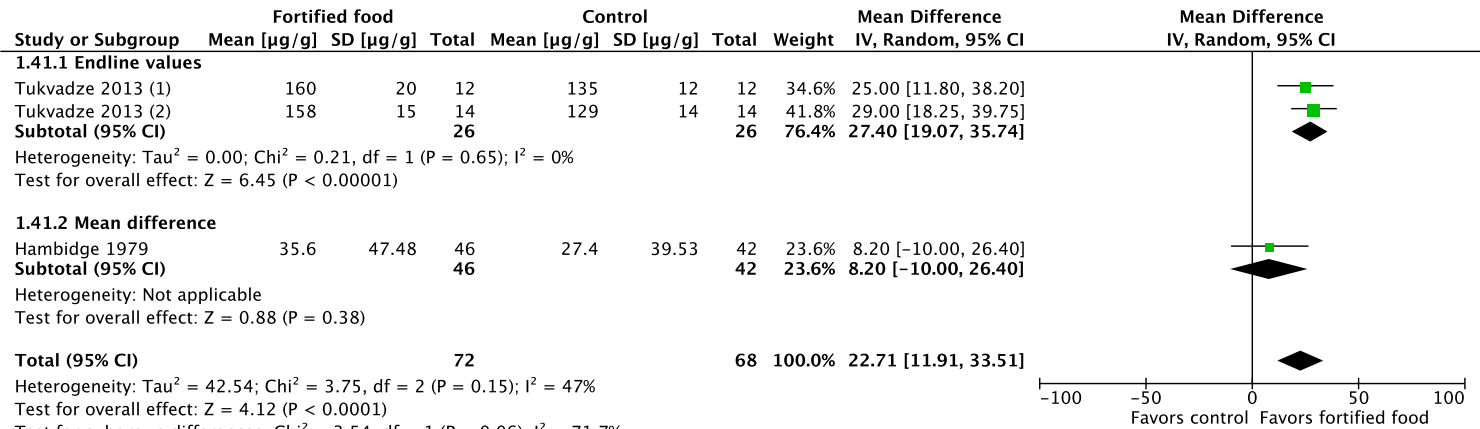

Footnotes

- (1) Boys
- (2) Girls

Supplement: nmab065_Supplemental_Files [file nmab065_supplemental_files.zip › Supplemental figure 38.pdf]

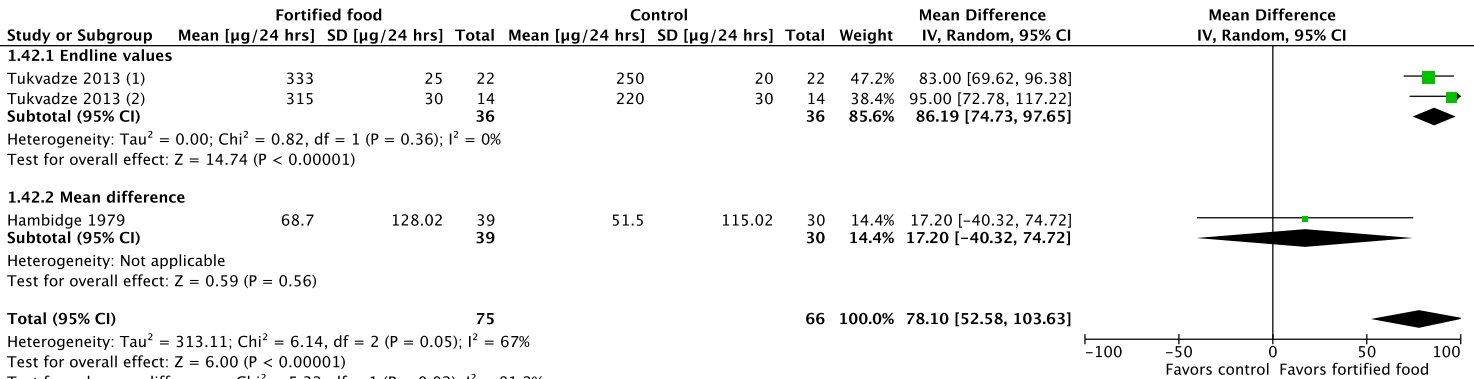

Footnotes

- (1) Girls
- (2) Boys

Supplement: nmab065_Supplemental_Files [file nmab065_supplemental_files.zip › Supplemental figure 39.pdf]

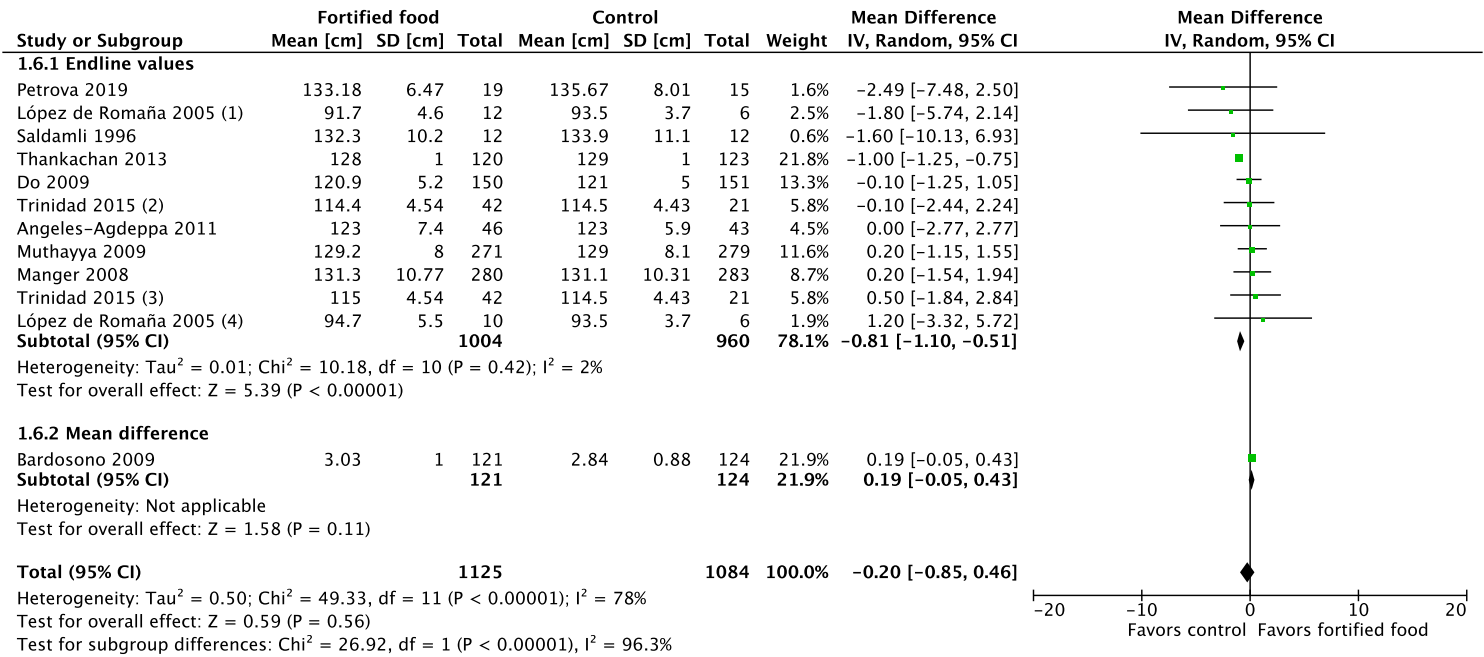

Footnotes

- (1) ZN0 v ZN9
- (2) 1 glass of fortified milk v. water
- (3) 2 glasses of fortified milk v. water
- (4) ZN0 v ZN3

Supplement: nmab065_Supplemental_Files [file nmab065_supplemental_files.zip › Supplemental figure 7.pdf]

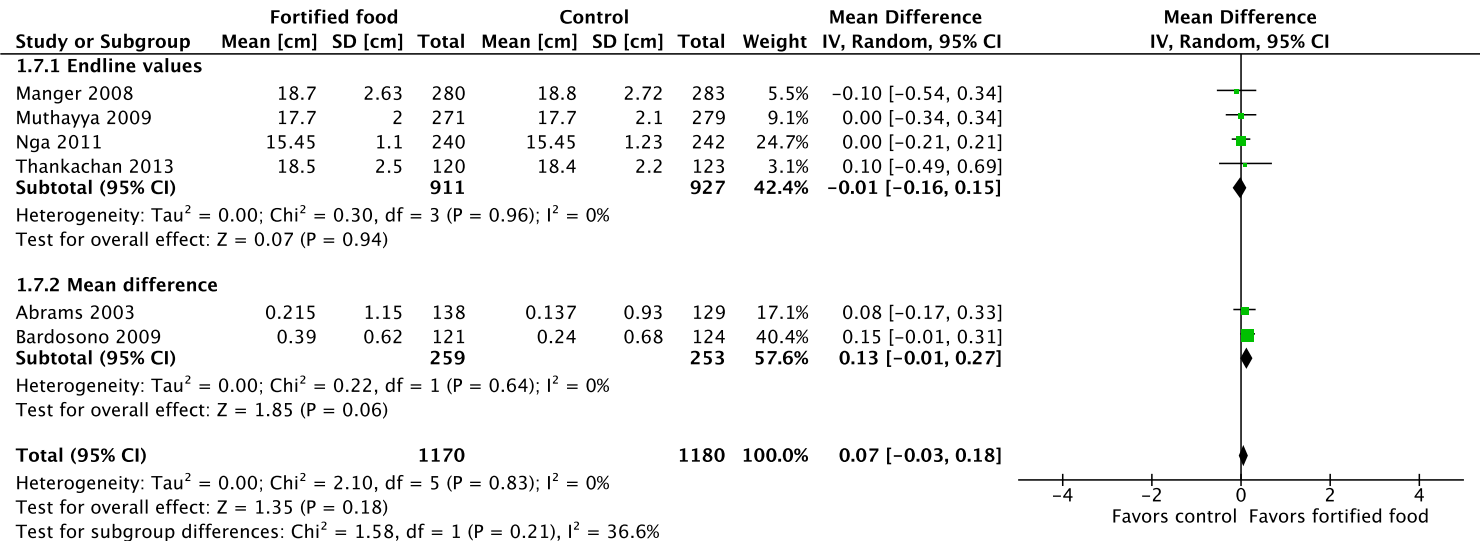

Supplement: nmab065_Supplemental_Files [file nmab065_supplemental_files.zip › Supplemental figure 8.pdf]

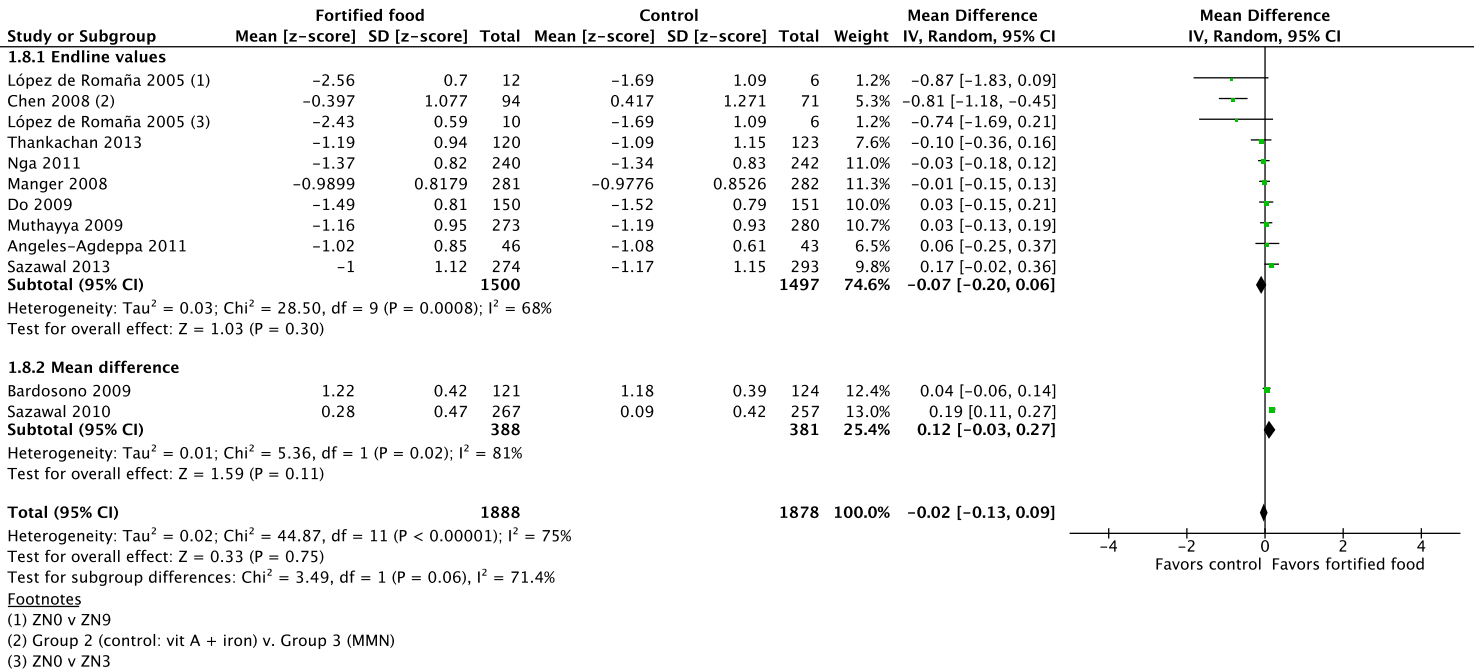

Supplement: nmab065_Supplemental_Files [file nmab065_supplemental_files.zip › Supplemental figure 9.pdf]
